# Leveraging senescence-oxidative stress co-relation to predict prognosis and drug sensitivity in breast invasive carcinoma

**DOI:** 10.3389/fendo.2023.1179050

**Published:** 2023-08-04

**Authors:** Yinghui Ye, Yulou Luo, Tong Guo, Chenguang Zhang, Yutian Sun, Anping Xu, Ling Ji, Jianghua Ou, Shang Ying Wu

**Affiliations:** ^1^ Department of Laboratory Medicine, Peking University Shenzhen Hospital, Shenzhen, China; ^2^ Department of Breast Surgery, Affiliated Tumor Hospital of Xinjiang Medical University, Urumqi, China; ^3^ Department of Medical Oncology, Sichuan Cancer Hospital and Institute, Sichuan Cancer Center, School of Medicine, University of Electronic Science and Technology of China, Chengdu, China

**Keywords:** breast cancer, senescence, oxidative stress, prognosis, tumor mutation burden, immune infiltration, single-cell analysis

## Abstract

**Introduction:**

Female breast cancer has risen to be the most common malignancy worldwide, causing a huge disease burden for both patients and society. Both senescence and oxidative stress attach importance to cancer development and progression. However, the prognostic roles of senescence and oxidative stress remain obscure in breast cancer. In this present study, we attempted to establish a predictive model based on senescence-oxidative stress co-relation genes (SOSCRGs) and evaluate its clinical utility in multiple dimensions.

**Methods:**

SOSCRGs were identified via correlation analysis. Transcriptome data and clinical information of patients with breast invasive carcinoma (BRCA) were accessed from The Cancer Genome Atlas (TCGA) and GSE96058. SVM algorithm was employed to process subtype classification of patients with BRCA based on SOSCRGs. LASSO regression analysis was utilized to establish the predictive model based on SOSCRGs. Analyses of the predictive model with regards to efficacy evaluation, subgroup analysis, clinical association, immune infiltration, functional strength, mutation feature, and drug sensitivity were organized. Single-cell analysis was applied to decipher the expression pattern of key SOSCRGs in the tumor microenvironment. Additionally, qPCR was conducted to check the expression levels of key SOSCRGs in five different breast cancer cell lines.

**Results:**

A total of 246 SOSCRGs were identified. Two breast cancer subtypes were determined based on SOSCRGs and subtype 1 showed an active immune landscape. A SOSCRGs-based predictive model was subsequently developed and the risk score was clarified as independent prognostic predictors in breast cancer. A novel nomogram was constructed and exhibited favorable predictive capability. We further ascertained that the infiltration levels of immune cells and expressions of immune checkpoints were significantly influenced by the risk score. The two risk groups were characterized by distinct functional strengths. Sugar metabolism and glycolysis were significantly upregulated in the high risk group. The low risk group was deciphered to harbor PIK3CA mutation-driven tumorigenesis, while TP53 mutation was dominant in the high risk group. The analysis further revealed a significantly positive correlation between risk score and TMB. Patients in the low risk group may also sensitively respond to several drug agents. Single-cell analysis dissected that ERRFI1, ETS1, NDRG1, and ZMAT3 were expressed in the tumor microenvironment. Moreover, the expression levels of the seven SOSCRGs in five different breast cancer cell lines were quantified and compared by qPCR respectively.

**Conclusion:**

Multidimensional evaluations verified the clinical utility of the SOSCRGs-based predictive model to predict prognosis, aid clinical decision, and risk stratification for patients with breast cancer.

## 1 Introduction

The number of patients diagnosed with breast cancer is rising at an alarming rate, and nearly 2.3 million new cases are reported annually (1). Thus the disease burden of breast cancer is rapidly accumulating and evolving to be a global public-health topic. Among the diverse histological types, breast invasive carcinoma (BRCA) accounts for over 70% of total breast cancer cases, including lobular invasive carcinoma and ductal invasive carcinoma (2). Currently, multidisciplinary treatments including chemo/radiotherapy, immunotherapy, targeted therapy, and traditional surgical resection provide renewal for the clinical management of breast cancer (3). With the aid of collaborative treatments, the mortality of breast cancer is now less than it was at previous times (4). However, the whole prognosis of breast cancer remains unfavorable due to tumor heterogeneity, and some patients have even died in the early stage (5, 6). Therefore, seeking potential novel biomarkers may contribute to improving the prognosis of patients with breast cancer, and in the meanwhile, robust predictive signature renders the clinical treatment more personalized based on the risk stratification of patients.

Reactive oxygen species (ROS) refer to a group of radicals that are generated as byproducts during normal cell metabolism, including O_2_·, H_2_O_2_, hydroxyl radical (OH·), etc., which are highly aggressive and harmful (7). To offset the cellular damage by ROS, antioxidants are employed as major power to prevent ROS-induced cellular damage by means of forming a dynamic balance with ROS (8). Once the balance is cracked, free radicals spray around and cause structural destruction, thereby inducing oxidative stress. One of the most lethal subsequences is irreversible DNA damage, which significantly affects genome mutation and instability, as well as epigenetic dysregulation (9). Consecutive DNA damage and a weakened genome repair system may lead to the mutation of pivotal oncogenes and tumor suppressor genes and finally trigger carcinogenesis (10). Moreover, previous studies have verified the potential and value of preventing ROS-related carcinogenesis by enhancing antioxidative defense, which demonstrates that ROS is important to cancer initiation (11–13).

Aging is another intriguing topic in life science. It is worth mentioning that Carlos et al. (14) summarized the top twelve hallmarks of aging. Several hallmarks of aging, such as genomic instability, epigenetic alterations, loss of proteostasis, and mitochondrial dysfunction, are significantly linked with oxidative stress, as a cause and/or subsequence. Current opinion lies that oxidative stress results in aberrant activation of pivotal signaling pathways, genome mutation and instability, and accumulation of impaired proteins thereby inducing subsequent outcomes like cancer and senescence (15). Furthermore, the morbidity of cancer is higher in older populations. This indicates the potential link between oxidative stress, senescence, and cancer, and that oxidative stress contributes to both senescence and cancer while aging originally correlates with cancer. According to this, several studies have evaluated the role of senescence or oxidative stress in predicting the prognosis of patients with breast cancer (16–18). Nevertheless, in breast cancer, the prognostic role of oxidative stress-senescence co-relation remains obscure and awaits further clarification.

Increasing evidence suggests that oxidative stress, senescence, and cancer are closely linked (15). In the present study, we aimed to detect the prognostic value of oxidative stress-senescence co-relation in BRCA by constructing a prognostic model. Comprehensive analyses including subtype identification, immune infiltration, immune checkpoint expression, functional characterization, mutation landscape, drug sensitivity, and single-cell analysis were orchestrated. The results of the present study may contribute to clinical risk stratification and personalized therapy, thereby improving the prognosis of patients with BRCA.

## 2 Materials and methods

### 2.1 Data collection and identification of genes characterized by senescence-oxidative stress co-relation

Transcriptome data and clinical information of patients with BRCA were downloaded from the TCGA database (http://cancergenome.nih.gov/) and the GSE96058 dataset was from the GEO database (https://www.ncbi.nlm.nih.gov/geo). The large cohort and high throughput sequencing method used in GSE96058 were taken as the reason for selecting it. The single-cell profile GSM5354529 was also accessed from the GEO database. Male BRCA samples and samples without survival information were excluded. In total, 965 BRCA samples from TCGA and 3409 BRCA samples from GSE96058 were finally enrolled. Then, 280 senescence-related genes (SRGs) were acquired from CellAge (http://genomics.senescence.info/cells) and 1401 oxidative stress-related genes (OSRGs) were obtained from a previous study (19). Pearson correlation analysis was conducted between each SRG and each OSRG. SRGs with *r* > 0.3 and *P* < 0.05 were considered to be significantly relevant to oxidative stress. The correlation network between SRGs and OSRGs are displayed in **Supplementary Figure S1**. We obtained 246 genes characterized by senescence-oxidative stress co-relation, which were named senescence-oxidative stress co-relation genes (SOSCRGs). SOSCRGs were further subject to Gene Ontology (GO) and Kyoto Encyclopedia of Genes and Genomes (KEGG) functional enrichment analyses based on clusterProfiler and org.Hs.eg.db R packages.

### 2.2 Determination of distinct subtypes in BRCA

We employed a non-negative matrix factorization (NMF) algorithm to divide the TCGA-BRCA cohort into different subtypes based on SOSCRGs. The optimal number of subtypes was determined according to cophenetic, dispersion, evar, residuals, rss, silhouette, and sparseness. Survival differences in the progression free survival (PFS) and overall survival (OS) between different subtypes were compared with survminer and survival R packages. Single sample gene set enrichment analyses (ssGSEA) were applied to quantify the level of immune activities and immune cell infiltration. The differences in immune activities and immune cell infiltration between different subtypes were also investigated. Next, we employed the ESTIMATE algorithm to calculate four indexes (TumorPurity, ESTIMATEScore, ImmuneScore, and StromalScore) and compared the level of the four indexes between different subtypes.

### 2.3 Construction and validation of the prognostic model

We randomly divided the TCGA-BRCA cohort into the training cohort (70%) and the internal validation cohort (30%). The transcriptome data of GSE96058 were supported by high throughput sequencing, which is compatible with TCGA. Thus GSE96058 was used as the external validation cohort. We subsequentially processed univariate Cox regression analysis and LASSO regression analysis of the SOSCRGs to further screen meaningful prognostic factors for BRCA in the training cohort. Multivariate Cox regression analysis in the glmnet R package was used to construct the prognostic model that contained seven SOSCRGs. The risk score was calculated as: Risk score = β_1_ * Gene_1_Exp + β_2_ * Gene_2_Exp + ··· + β_n_ * Gene_n_Exp. The low risk group and the high risk group were evenly divided according to the median risk score value. Survival difference between the low risk group and the high risk group was compared. We also evaluated the predictive capability of the prognostic model by depicting receiver operating characteristic (ROC) curves with the timeROC R package. The analyses described above were performed in internal and external validation cohorts respectively.

### 2.4 Subgroup analysis and establishment of prognostic nomogram

To testify to the applicability of the prognostic model in a broader way, we applied the prognostic model in ten different clinical subgroups. Univariate and multivariate Cox regression analyses were processed to decipher independent prognostic predictors from risk score and other clinical factors in the TCGA-BRCA cohort. Next, we established a prognostic nomogram based on risk score and other clinical factors to further amplify the prognostic value of SOSCRGs. The predictive accuracy of the nomogram was verified by calibration curves.

### 2.5 Analyses of immune infiltration and immune checkpoint expression

We quantified the infiltration levels of diverse immune cells by both CIBERSORT and ssGSEA algorithms, thereby comparing the difference in immune cell infiltration and immune activities between the low risk group and the high risk group. The correlations between the seven SOSCRGs and infiltration levels of immune cells were analyzed by Pearson correlation analysis. Moreover, we investigated the expression pattern of immune checkpoints between the two risk groups to ascertain the potential value of the prognostic model in immunotherapy.

### 2.6 Functional enrichment analysis and drug sensitivity analysis

The differentially expressed genes (DEGs) between the low risk group and the high risk group were deciphered. Gene Set Enrichment Analysis (GSEA) was performed based on the DEGs to check out the functional characterizations in the two risk groups. The pRRophetic R package was used to process wide drug screening based on the GDSC database (https://www.sanger.ac.uk/tool/gdsc-genomics-drug-sensitivity-cancer) to ascertain the drugs that the two risk groups may sensitively respond to.

### 2.7 Mutation landscapes of the two risk groups

The mutation landscapes of the low risk group and high risk group were obtained via the maftools R package respectively. The top twenty most frequently altered genes in the two risk groups were displayed respectively. The difference in tumor mutation burden (TMB) between the low risk and high risk groups were then examined. The low TMB group and high TMB group were also divided according to the median cut-off value of TMB. The survival differences between patients in the low-TMB group and high-TMB group with or without a combination of risk groups were further uncovered. Furthermore, Pearson correlation analysis was conducted to determine the correlation between risk score and TMB.

### 2.8 Single-cell analysis

We took the single-cell profile GSM5354529 for single-cell analysis. Approximately 8402 high quality cells were filtered from GSM5354529. The filter conditions were as follows: subset = nFeature_RNA > 100 & nFeature_RNA < 5000 & percent.mt < 25 & nCount_RNA > 100. The expression pattern of the seven SOSCRGs in the tumor microenvironment were then determined by the Seurat R package.

### 2.9 Cell culture

Five human breast cancer cell lines MCF-7, BT-20, MDA-MB-231, HCC1806, and HCC1937 were purchased from Wuhan Procell Life Science and Technology Co., Ltd. (Wuhan, China). Each cell line was cultured in its dedicated medium (Wuhan Procell Life Science and Technology Co. Ltd., Wuhan, China). Cells were cultured in RPMI-1640 (Gibco-BRL), supplemented with 10% fetal bovine serum (Bioserum), 100 U/mL penicillin G, and 100 μg/mL streptomycin.

### 2.10 Quantitative PCR

Total RNA was extracted from each cell line using TRIzol Reagent (Cat. No. P118-05, GenStar, Beijing, China) according to the manufacturer’s instructions. The total RNA was amplified by qPCR using SYBR Green Master Mix (Cat#: C0006, TOPSCIENCE, Shanghai, China) according to the manufacturer’s instructions, and the mRNA levels of CRGs and CRLncs were detected. The primer pairs of the seven SOSCRGs were synthesized by Accurate Biology (Changsha, China) and are listed in the **Supplementary Materials**.

### 2.11 Statistical analysis

Bioinformatic analyses were all conducted by R 4.0.3. The comparison of the K-M survival curve was achieved by Cox regression analysis. The differences in expression level between groups were compared by the Wilcoxon rank sum test. Pearson correlation was taken for correlation analysis. |*r*| > 0.1 was considered to be relevant and *P* < 0.05 was deemed as statistically significant. “*” indicates *P* < 0.05, “**” indicates *P* < 0.01 and “***” indicates *P* < 0.001 throughout this study.

## 3 Results

### 3.1 Two distinct subtypes were identified in BRCA based on senescence-oxidative stress co-relation

**Figure 1:**
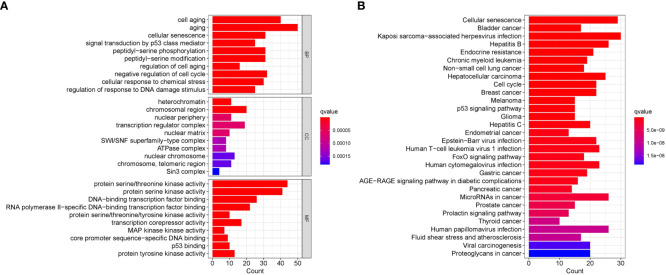
Functional enrichment analysis of the identified SOSCRGs. **(A)** GO functional enrichment analysis of SOSCRGs. **(B)** KEGG functional enrichment analysis of SOSCRGs.

**Figure 2:**
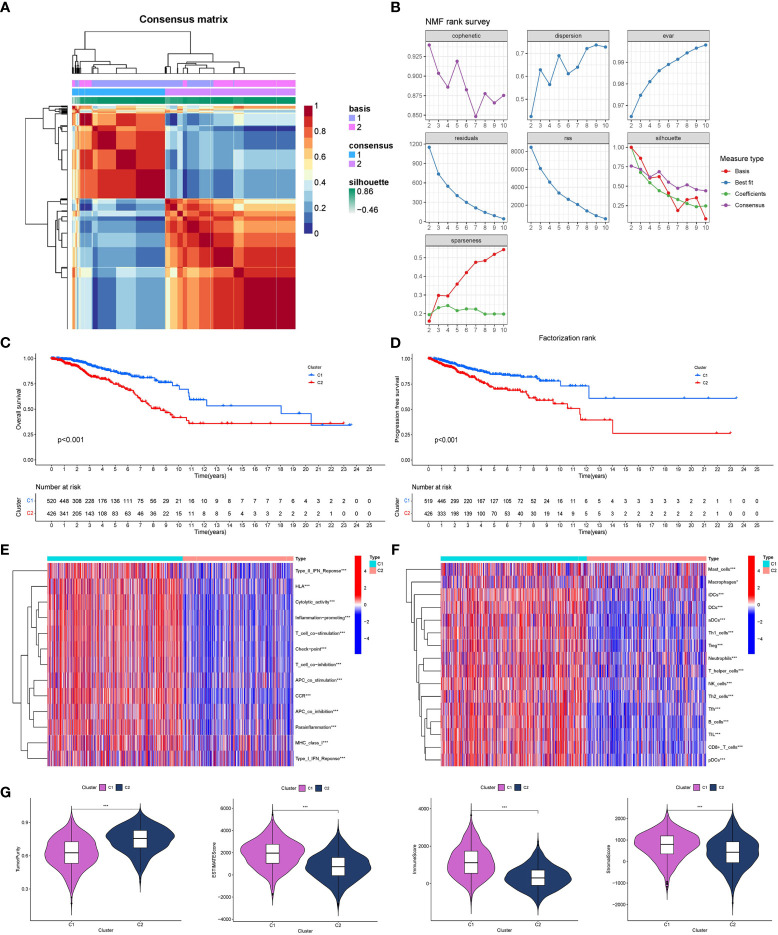
Determination of breast cancer subtypes based on SOSCRGs. **(A)** Consensus matrix heatmap by SVM algorithm. **(B)** Relationship between cophenetic, dispersion, evar, residuals, rss, silhouette, and sparseness coefficients with respect to the number of subtypes. Survival difference in OS **(C)** and PFS **(D)** between subtype 1 and subtype 2. Differences in immune activities **(E)** and immune cell infiltration **(F)** between subtype 1 and subtype 2. **(G)** Differences in four tumor microenvironment indexes between subtype 1 and subtype 2. “*” indicates *P* < 0.05, “**” indicates *P* < 0.01 and “***” indicates *P* < 0.001.

GO/KEGG functional enrichment analysis strengthened the idea that the SOSCRGs are closely relevant to cell aging, DNA binding, DNA damage, protease activity, and breast cancer, which verified that the identified SOSCRGs were senescence-oxidative stress double functional in BRCA (**Figure 1**). Supporting vector machine (SVM) learning consensus matrix heatmap was depicted and the optimal number of subtypes was identified as 2 (k = 2) according to the cophenetic, dispersion, and silhouette curves (**Figures 2A, B**). Kaplan-Meier survival analysis revealed that subtype 2 harbored worse overall survival (OS) and progression free survival (PFS) compared with subtype 1 (**Figures 2C, D**). Subsequently, we dissected the tumor microenvironment characteristics of the two subtypes. We found that both the immune activities and immune cell infiltration levels of subtype 2 were poorer than subtype 1 (**Figures 2E, F**). Further investigation showed that, compared with subtype 1, subtype 2 had higher tumor purity as well as lower ESTIMATE score, immune score, and stromal score (**Figure 2G**).

### 3.2 A seven-SOSCRGs prognostic model was constructed and validated

**Figure 3:**
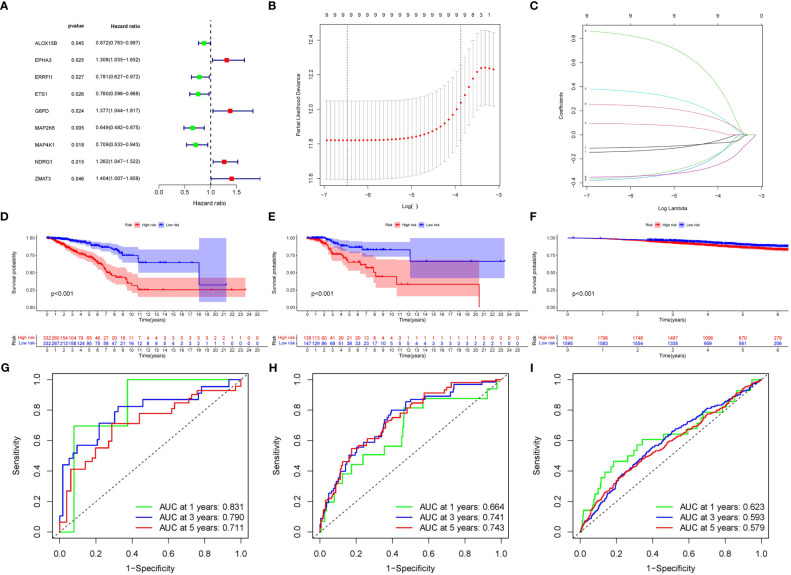
Construction and validation of the SOSCRGs-based prognostic model in breast cancer. **(A)** Univariate Cox regression of the SOSCRGs. **(B, C)** LASSO regression analysis of the SOSCRGs. **(D-F)** Survival differences between the low risk group and the high risk group in the training cohort, internal validation cohort, and external validation cohort. **(G-I)** ROC curves at 1-, 3-, and 5 years in the training cohort, internal validation cohort, and external validation cohort.

**Figure 4:**
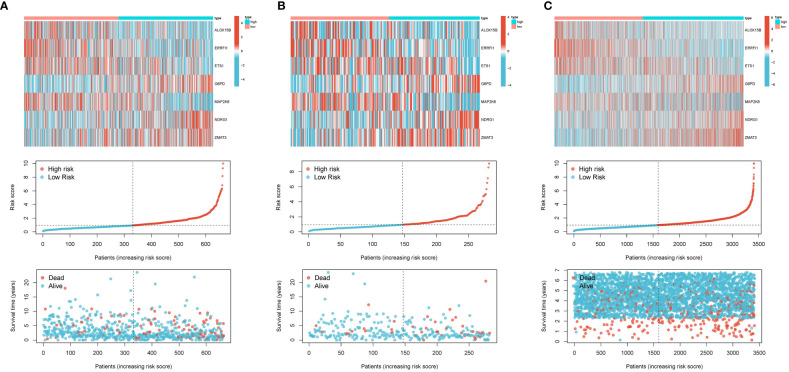
Expression pattern of the seven SOSCRGs between different risk groups and distribution of survival time with risk score. **(A)** Training cohort. **(B)** Internal validation cohort. **(C)** External validation cohort.

A total of nine SOSCRGs were found to be prognostic in BRCA by univariate Cox regression analysis in the training cohort (**Figure 3A**). None of the nine prognostic SOSCRGs were excluded by subsequent LASSO regression analysis (**Figures 3B, C**). Next, seven SOSCRGs were screened by multivariate Cox regression analysis to construct the prognostic model with the exclusion of two insignificant SOSCRGs. Risk score = (-0.149) * ALOX15B + (-0.376) * ERRFI1 + (-0.439) * ETS1 + 0.385 * G6PD + (-0.380) * MAP2K6 + 0.266 * NDRG1 + 0.966 * ZMAT3. The survival difference between the low risk group and the high risk group in the training cohort, internal validation cohort, and external validation cohort were significantly differentiated respectively (**Figures 3D–F**). The area under the curve from the ROC curve was used to evaluate the predictive efficacy of the model. The AUCs at 1-, 3-, and 5 years were 0.831, 0.79, and 0.711 in the training cohort (**Figure 3G**). The AUCs at 1-, 3-, and 5 years were 0.664, 0.741, and 0.743 in the internal validation cohort (**Figure 3H**). The AUCs at 1-, 3-, and 5 years were 0.623, 0.593, and 0.579 in the external validation cohort (**Figure 3I**). We also compared the predictive efficacy of the risk score and other clinicopathological features via ROC curves in the training cohort, internal validation cohort, and external validation cohort respectively (**Supplementary Figure S2**). The expression pattern of the seven SOSCRGs between different risk groups and distribution of survival time with risk score in the training cohort, internal validation cohort, and external validation cohort were displayed respectively (**Figures 4A–C**). These results indicated the robust predictive efficacy of the prognostic model.

### 3.3 The prognostic model was generally applicable and a predictive nomogram was established

**Figure 5:**
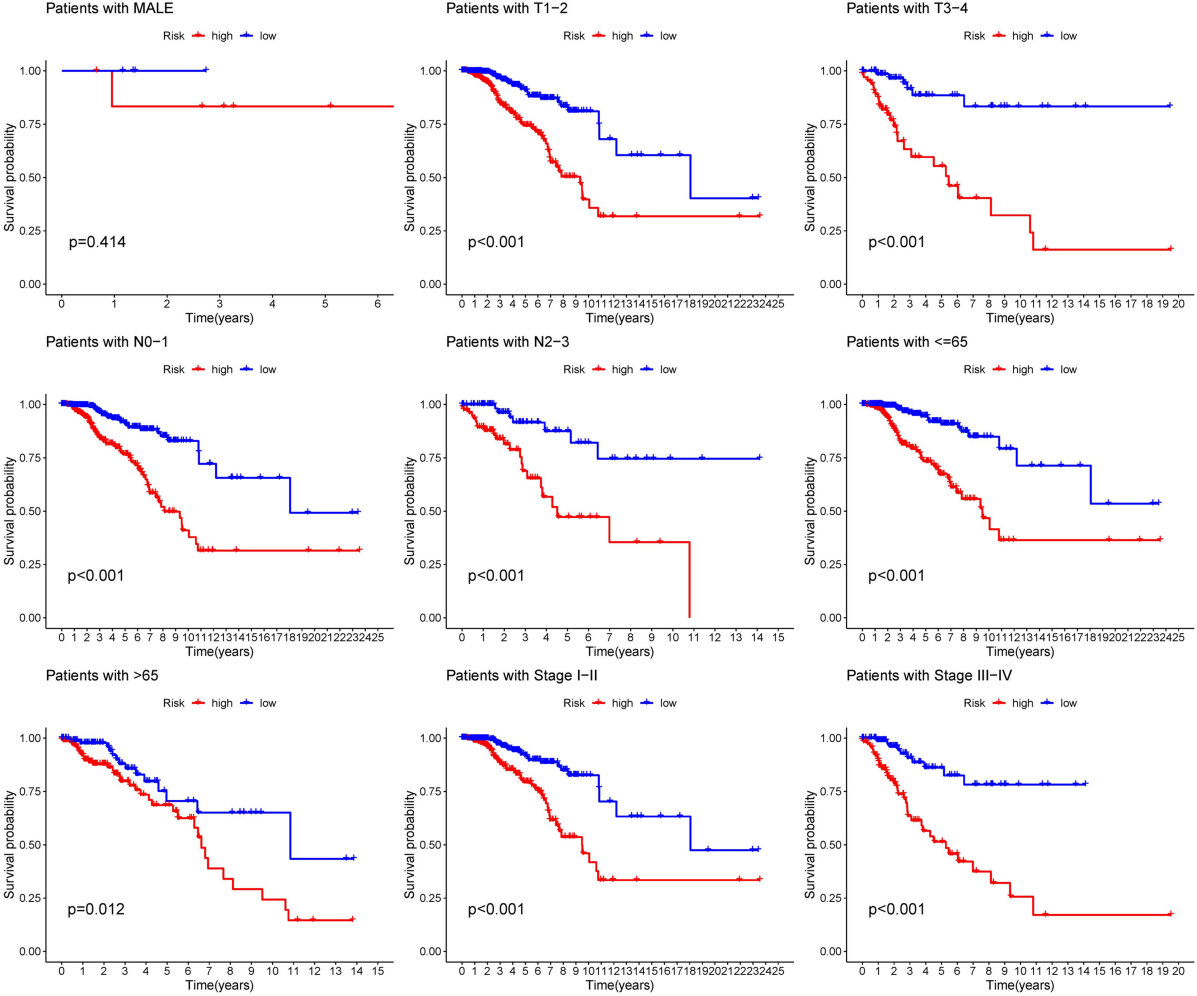
Subgroup analysis of the prognostic model in nine clinical subgroups.

**Figure 6:**
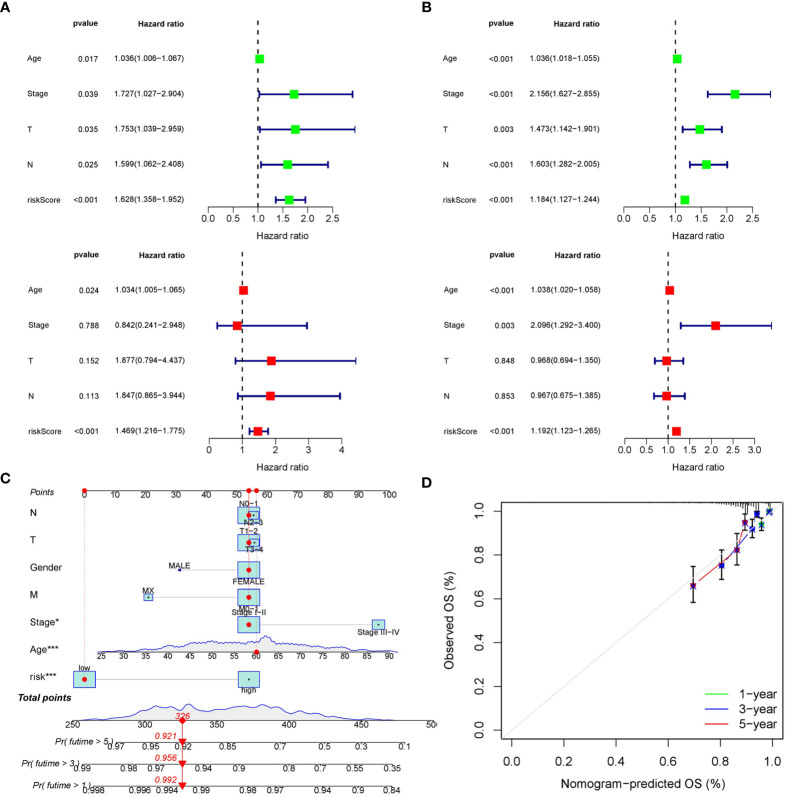
Construction of the predictive nomogram. **(A)** Univariate and multivariate Cox regression analyses in the training cohort. **(B)** Univariate and multivariate Cox regression analyses in the internal validation cohort. **(C)** Predictive nomogram based on the risk score and other clinicopathological features. **(D)** Calibration curves at 1-, 3-, and 5 years.

The distribution of risk score and clinicopathological features between the low risk group and the high risk group were displayed (**Supplementary Figure S3**). The results from subgroup analysis verified that the prognostic model can generally be applied regardless of multiple clinicopathological characteristics apart from patients that are male (**Figure 5**). Univariate and multivariate Cox regression analyses determined the risk score as an independent prognostic predictor for BRCA in both the training cohort and internal validation cohort, indicating the strong prognostic power of the predictive model (**Figures 6A, B**). Moreover, we established a predictive model to further explore the prognostic value of the risk score and other clinicopathological features (**Figure 6C**). Calibration curves showed that the predictive lines were close to the ideal line at 1-, 3-, and 5 years (**Figure 6D**).

### 3.4 Low risk group harbored more abundant immune infiltration levels and higher immune checkpoints expressions

**Figure 7:**
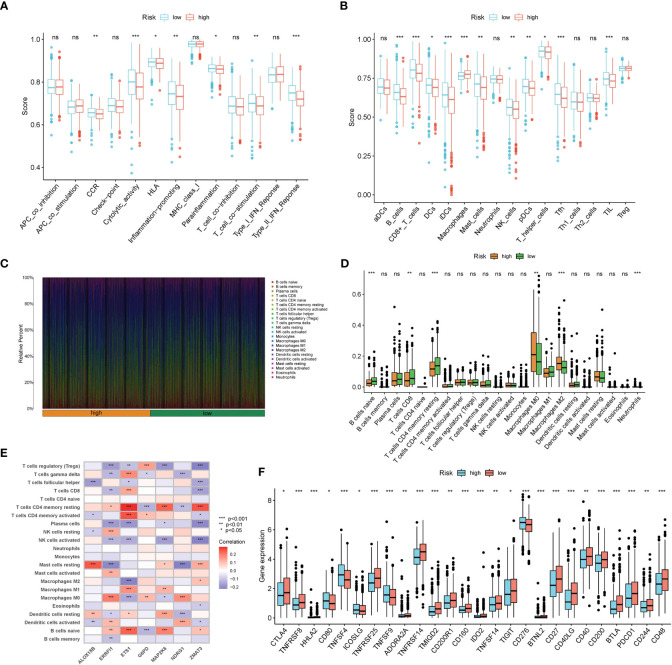
Dissection of the tumor microenvironment of the two risk groups. **(A, B)** Estimation of immune activities and immune infiltrating cells between the low risk group and the high risk group by ssGSEA. **(C)** Proportion of 22 immune infiltrating cells for each BRCA sample by CIBERSORT. **(D)** Estimation of 22 immune infiltrating cells between the low risk group and the high risk group by CIBERSORT. **(E)** Correlations between seven SOSCRGs and 22 immune infiltrating cells. **(F)** The expression pattern of immune checkpoints between the low risk group and the high risk group. “*” indicates *P* < 0.05, “**” indicates *P* < 0.01 and “***” indicates *P* < 0.001. "ns" indicates non-significant.

Results from ssGSEA ascertained that several immune activities (cytolytic activity, T cell co-stimulation, and type II IFN response, etc.) and immune infiltrating cells (B cells, CD8+ T cells, NK cells, and TIL, etc.) were higher in the low risk group (**Figures 7A, B**). CIBERSORT consistently suggested that the infiltration levels of B cells naive, T cells CD8, and T cells CD4 memory resting were higher in the low risk group (**Figures 7C, D**). Correlations between the seven SOSCRGs and 22 immune infiltrating cells were also unfolded (**Figure 7E**). Diverse immune infiltrating cells were significantly correlated with the seven SOSCRGs, such as T cells CD4 memory resting, T cells regulatory, B cells naive, and NK cells activated, etc., indicating potential functional associations. Furthermore, we found that the expression levels of a majority of immune checkpoints were significantly higher in the low risk group compared with the high risk group, including PDCD1, CTLA4, and TIGIT. (**Figure 7F**). These results indicated that the low risk group rendered immune-active, whereas the high risk group was relatively immune-cold. Patients in the low risk group may better benefit from immunotherapy based on their active tumor microenvironment.

### 3.5 The two risk groups were characterized by distinct functional strengths

**Figure 8:**
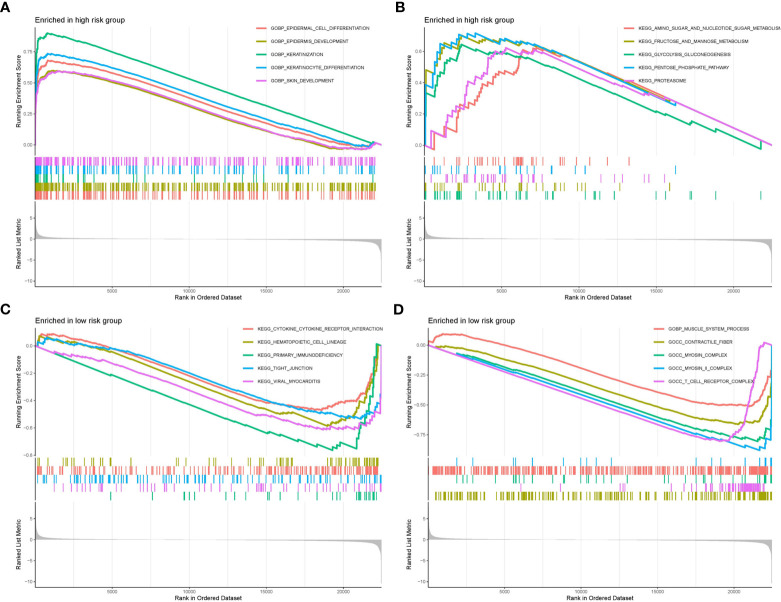
GSEA functional enrichment analysis of the two risk groups. **(A, B)** Functional strengthens in the high risk group. **(C, D)** Functional strengthens in the low risk group.

We selected the top ten significantly strengthened functional annotations of the two risk groups via GSEA to display (**Figures 8A–D**). Epidermal cell differentiation, keratinization, skin development, amino sugar and nucleotide metabolism, fructose and mannose metabolism, glycolysis gluconeogenesis, pentose phosphate pathway, and proteasome were strengthened in the high risk group. Cytokine-cytokine receptor interaction, hematopoietic cell lineage, primary immunodeficiency, tight junction, viral myocarditis, muscle system process, contractile fiber, myosin complex, and T cell receptor complex were strengthened in the low risk group.

### 3.6 The mutation features of the two risk groups were different

**Figure 9:**
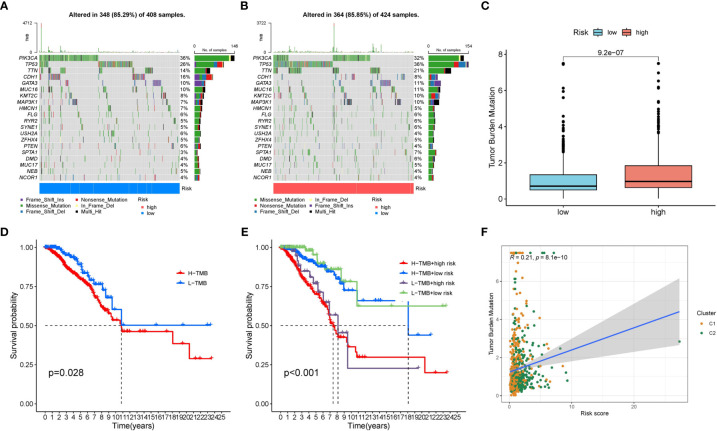
The mutation characteristics of the two risk groups. **(A)** The mutation landscape of the low risk group. **(B)** The mutation landscape of the high risk group. **(C)** Difference in TMB between the two risk groups. **(D)** Survival difference between low TMB group and high TMB group. **(E)** Survival difference between low TMB group and high TMB group combined with risk groups. **(F)** Correlation between the risk score and TMB.

We displayed the top 20 most frequently altered genes in the low risk group and high risk group. PIK3CA (36%) and TP53 (36%) were deciphered to be the most frequently altered genes in the low risk and high risk groups respectively, and the most common mutation type was observed to be missense mutation (**Figures 9A, B**). We also compared the TMB difference between the two risk groups which turned out to be statistically significant (**Figure 9C**). Patients with high TMB harbor poorer clinical outcomes compared to patients with low TMB (**Figure 9D**). Survival analysis combining risk score and TMB revealed that patients carrying low TMB and low risk score have the best prognosis, while patients carrying high TMB and high risk score suffered from the worst prognosis (**Figure 9E**). In addition, the risk score was significantly positively correlated with TMB (**Figure 9F**).

### 3.7 Patients in the low risk group were potentially sensitive to several drug agents

**Figure 10:**
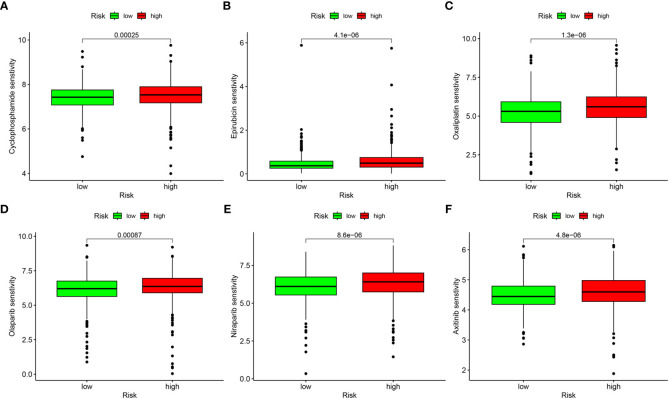
Drug sensitivity analysis. **(A)** Cyclophosphamide. **(B)** Epirubicin. **(C)** Oxaliplatin. **(D)** Olaparib. **(E)** Niraparib. **(F)** Axitinib.

We processed wide drug screening to determine potential drug agents that patients may sensitively respond to. These analyses determined three types of drug agents for patients in the low risk group: traditional chemotherapeutic drug agents (cyclophosphamide, epirubicin, and oxaliplatin) (**Figures 10A–C**), PARP inhibitors (olaparib and niraparib) (**Figures 10D, E**), and tyrosine kinase inhibitor (axitinib) (**Figure 10F**).

### 3.8 Single-cell analysis

**Figure 11:**
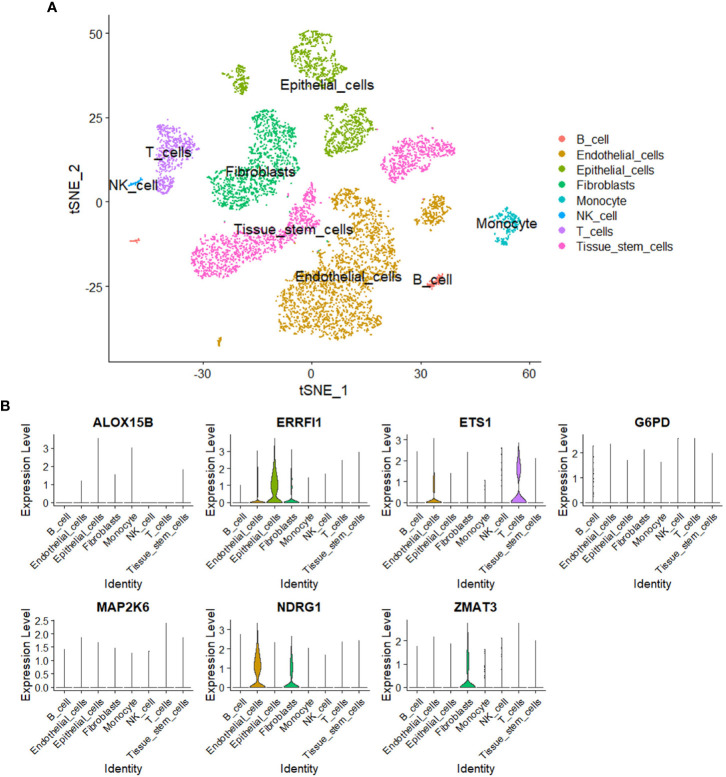
Single-cell analysis. **(A)** Identification of cell subgroups in the tumor microenvironment of breast cancer. **(B)** Expression pattern of the seven SOSCRGs in the tumor microenvironment.

A total of eight cell subgroups were identified in the tumor microenvironment of breast cancer, and endothelial cells, epithelial cells, and fibroblasts appeared to be the main cell subgroups (**Figure 11A**). The expression pattern of the seven SOSCRGs were subsequently determined. ERRFI1 was expressed in endothelial cells, epithelial cells, and fibroblasts. ETS1 was expressed in endothelial cells and T cells. NDRG1 was expressed in endothelial cells and fibroblasts. ZMAT3 was expressed in fibroblasts (**Figure 11B**).

### 3.9 The mRNA levels of seven SOSCRGs in breast cancer cell lines

**Figure 12:**
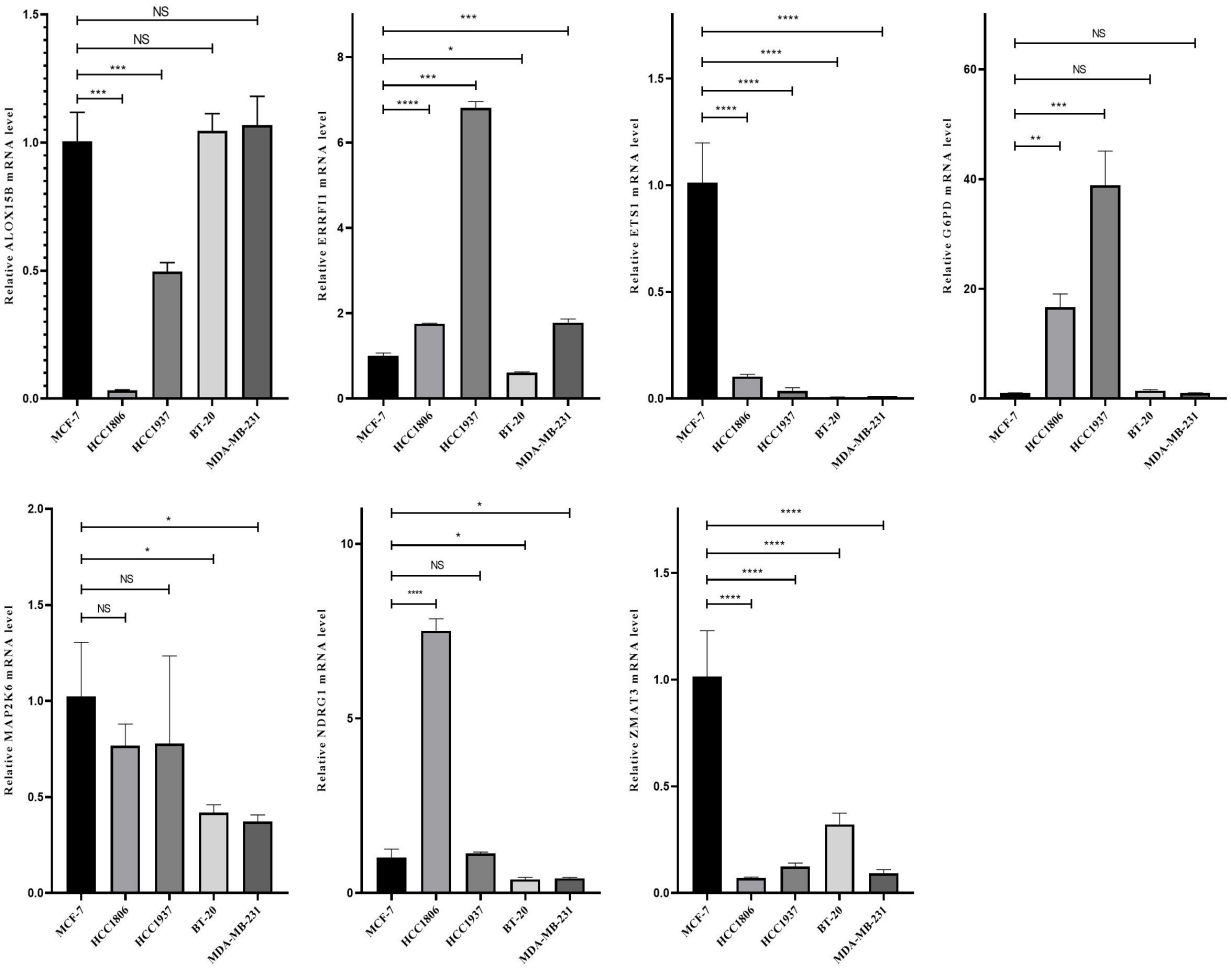
The mRNA levels of the seven SOSCRGs in five breast canceer cell lines. “*” indicates *P* < 0.05, “**” indicates *P* < 0.01 and “***” indicates *P* < 0.001. "ns" indicates non-significant.

We demonstrated the mRNA levels of the seven SOSCRGs in five breast cancer cell lines respectively using qPCR (**Figure 12**).

## 4 Discussion

Carcinogenesis increases with aging, which can be partially explained by it sharing several similar hallmarks, such as genomic instability, epigenetic alterations, loss of proteostasis, altered intercellular communication, and chronic inflammation (14, 20). Current opinion also suggests that epigenetic entropy increases with aging and is relevant to DNA damage (21). Meanwhile, oxidative stress is one of the most important origins that induce DNA damage (22). Therefore, the intimacy between senescence, oxidative stress, and cancer is intriguing and should be further explored. The need for established predictive models for these diverse clinical end events in cancer is growing more urgent, including information on survival, recurrence, and metastasis. Several previous studies have reported on how models were used to predict the prognosis of patients with breast cancer based on unilateral senescence-related or oxidative stress-related genes (16–18). Compared with these models, the predictive model constructed in the present study may have the following advantages: the first being that the genes characterized by senescence-oxidative stress co-relation were used to construct the present model, meaning it was initiative. Secondly, AUCs from the time-dependent ROC curves of the present model exhibited the highest, which were 0.831, 0.79, and 0.711 at 1-, 3-, and 5 years. Additionally, we dissected the expression pattern of the seven SOSCRGs in the tumor microenvironment of breast cancer by single-cell analysis.

SVM classification is a powerful machine learning method that is widely applied in cancer subtyping (23). We identified two subtypes by SVM classification in BRCA based on SOSCRGs. Interestingly, subtype 1 exhibited active immune activities and abundant immune cell infiltration in the tumor microenvironment (immune-active), whereas subtype 2 showed much lower immune activities and immune cell infiltration levels (immune-cold). Higher infiltration levels of immune cells in the tumor microenvironment infer priority to mobilize the intratumoral immune system to slash cancer cells, which also means better response to immunotherapy (24). Thus patients in the SOSCRGs-based subtype 1 may potentially better benefit from immunotherapy, which can contribute to clinical decisions.

Immune infiltrating cells in the tumor microenvironment are attributed to their important roles in affecting the prognosis of cancer, especially tumor-infiltrating lymphocytes (TILs) (25–27). High infiltration levels of T cells are often supposed to be associated with a better prognosis (28, 29). Studies have examined whether CD4 + T cells alleviate CD8 + T cells exhaustion, and high infiltration levels of CD4 + T cells and CD8 + T cells predicted better survival for patients with breast cancer (30, 31). We also identified higher infiltration levels of both CD8 + T cells and CD4 + T cells memory resting in the low risk group that harbored favorable clinical outcomes.

In recent years, immunotherapy has risen to become a first-line anti-cancer strategy. Identification of the expression patterns of immunotherapy targets may provide potential survival priority for patients (32). Studies have shown that the combined blockade of PD1 and CTLA4 achieves better prognosis improvement compared to monotherapy in several cancer types (33–35). Our results showed that the expression levels of PD1, TIGIT, and CTLA4 were higher in the low risk group, suggesting co-blockade of these molecules as a new immune checkpoint blockade strategy for patients in the low risk group. This also indicates the value of the present model in aiding clinical treatment.

Metabolic reprogramming has been widely observed in diverse cancer types to facilitate cell growth and proliferation. Aberrant enhancement of glycolysis is an important component in cancer metabolic reprogramming, which also refers to the Warburg effect (36). Elevated glycolysis level generally infers an unfavorable prognosis (37, 38). We found several strengthened functional terms relevant to sugar metabolism in the high risk group (amino sugar and nucleotide metabolism, fructose and mannose metabolism, glycolysis gluconeogenesis, and pentose phosphate pathway), which may explain the corresponding worse prognosis. Besides, targeting glycolysis may also serve as a potential therapeutic strategy for patients in the high risk group.

Excessive mutation of tumor suppressor genes originally accelerates carcinogenesis (39). Both PIK3CA and TP53 are common mutated oncogenes in breast cancer (40). We identified PIK3CA and TP53 as potential carcinogenic driving genes in the low risk group and the high risk group respectively. A previous study found that the prognostic effects of PIK3CA and TP53 mutations were different in patients with early breast cancer (41). Patients with PIK3CA-only mutation harbored relatively favorable disease-free interval (DFI), whereas patients with TP53-only mutation harbored worse DFI, more importantly, patients with PIK3CA-TP53 co-mutation exhibited the worst DFI (41). This also proved that the prognosis of patients with dominant TP53 mutation in the high risk group was worse than patients with dominant PIK3CA mutation in the low risk group. Additionally, patients in the high risk group had higher TMB than patients in the low risk group, and the risk score was significantly positively correlated with TMB. This indicated that patients in the high risk group suffered from more mutation accumulation, thereby contributing to unsatisfactory clinical results.

Single-cell transcriptome data is sequenced from annotated cells with high quality, which renders it more precise than common bulk RNA-sequencing data. Thus it is widely applied to dissect the tumor microenvironment to further understand the intratumoral heterogeneity (42–44). In the present study, we dissected the expression pattern of SOSCRGs in the tumor microenvironment of breast cancer based on single-cell analysis. We found that endothelial cells, epithelial cells, and fibroblasts appeared to be the main cell subgroups. Only the expressions of ERRFI1, ETS1, NDRG1, and ZMAT3 were detected in the tumor microenvironment. Furlan et al. (45) reported the pivotal role of ETS1 in modulating the links between breast cancer cells and endothelial cells and facilitating intratumoral angiogenesis. Thus it is that these SOSCRGs that may serve as media molecules during cancer cells-tumor microenvironment interactions to affect tumor development and progression. However, more experimental evidence is waiting to be accomplished.

The present study has several limitations. Above all, it would be better to verify the expressions of the seven SOSCRGs in breast cancer using clinical specimens. Apart from that, the application of the SOSCRGs-based predictive model in a prospective cohort would further prove its clinical utility. In addition, as we mentioned above, the regulatory network and mechanisms of the SOSCRGs in breast cancer require further elucidation by more experimental evidence.

## 5 Conclusion

Two distinct BRCA subtypes were determined based on SOSCRGs, among which subtype 1 was immune-active and subtype 2 was immune-cold, and the clinical outcomes between patients in the two subtypes were significantly different. A seven-SOSCRGs-based predictive model was constructed and validated to fairly predict the prognosis for patients with BRCA. Subgroup analysis verified the applicability of the predictive model. The risk score was deciphered as an independent prognostic predictor by univariate and multivariate Cox regression analysis. A prognostic nomogram integrated with the risk score and clinicopathological characteristics was established and identified to harbor robust predictive efficacy for patients with BRCA. We further found that the two risk groups had distinct immune infiltration patterns, immune checkpoint expression patterns, functional strengths, and mutation landscapes. Three types of drug agents were predicted to be potentially sensitive to patients in the low risk group. Furthermore, the expression patterns of the seven SOSCRGs in the tumor microenvironment were dissected by single-cell analysis. Multidimensional investigations verified that the SOSCRGs-based predictive model may provide new insights into prognosis prediction, risk stratification, and clinical decision for patients with BRCA.

## Data availability statement

The datasets presented in this study can be found in online repositories. The names of the repository/repositories and accession number(s) can be found in the article/**Supplementary Material**.

## Ethics statement

Written informed consent was obtained from the individual(s), and minor(s)’ legal guardian/next of kin, for the publication of any potentially identifiable images or data included in this article.

## Author contributions

YY conducted the bioinformatic analysis and qPCR experiments. YY, YL, and TG drafted the manuscript. CZ, YS, AX, and LJ integrated all the data and edited the manuscript. JO and SYW outlined the study and gave final approval of the manuscript. All authors have agreed to be accountable for the content of this study. All authors contributed to the article and approved the submitted version.
