# Supplementary material for: Leveraging senescence-oxidative stress co-relation to predict prognosis and drug sensitivity in breast invasive carcinoma
Source: Front Endocrinol (Lausanne). 2023 Aug 4;14:1179050. doi: 10.3389/fendo.2023.1179050 (PMC10437062; doi:10.3389/fendo.2023.1179050)
Supplement: Supplementary file 1 [file DataSheet_1.zip › 2.NMF/heatmap.all.pdf]

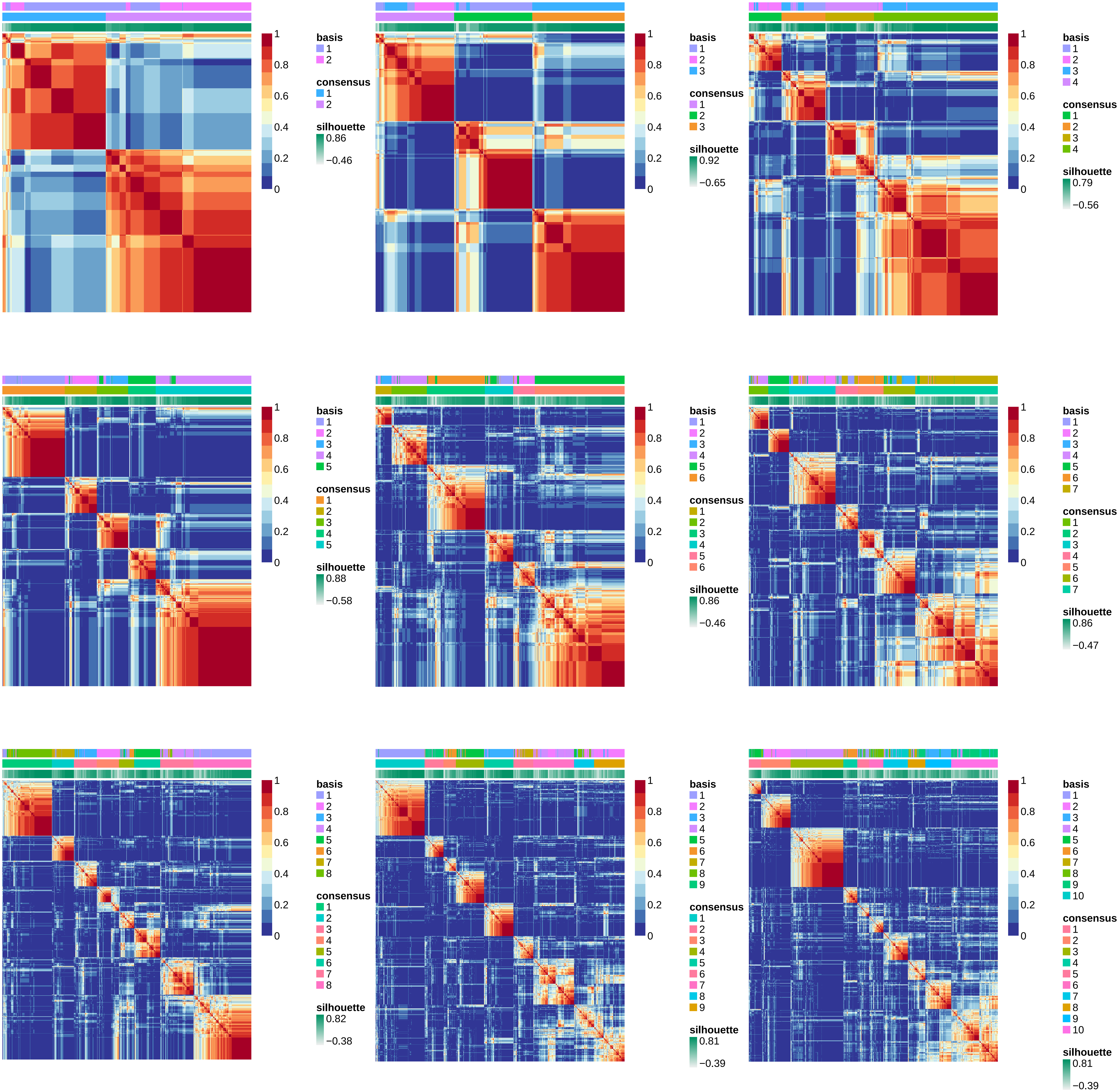
1
0.8
0.6
0.4
0.2
0
basis
1
2
consensus
1
2
silhouette
0.86
−0.46
basis
1
2
3
consensus
1
2
3
silhouette
0.92
−0.65
basis
1
2
3
4
consensus
1
2
3
4
silhouette
0.79
−0.56
basis
1
2
3
4
5
consensus
1
2
3
4
5
silhouette
0.88
−0.58
basis
1
2
3
4
5
6
consensus
1
2
3
4
5
6
silhouette
0.86
−0.46
basis
1
2
3
4
5
6
7
consensus
1
2
3
4
5
6
7
silhouette
0.86
−0.47
basis
1
2
3
4
5
6
7
8
consensus
1
2
3
4
5
6
7
8
silhouette
0.82
−0.38
basis
1
2
3
4
5
6
7
8
9
consensus
1
2
3
4
5
6
7
8
9
silhouette
0.81
−0.39
basis
1
2
3
4
5
6
7
8
9
10
consensus
1
2
3
4
5
6
7
8
9
10
silhouette
0.81
−0.39
