# Supplementary figures and images for: Leveraging senescence-oxidative stress co-relation to predict prognosis and drug sensitivity in breast invasive carcinoma
Source: Front Endocrinol (Lausanne). 2023 Aug 4;14:1179050. doi: 10.3389/fendo.2023.1179050 (PMC10437062; doi:10.3389/fendo.2023.1179050)

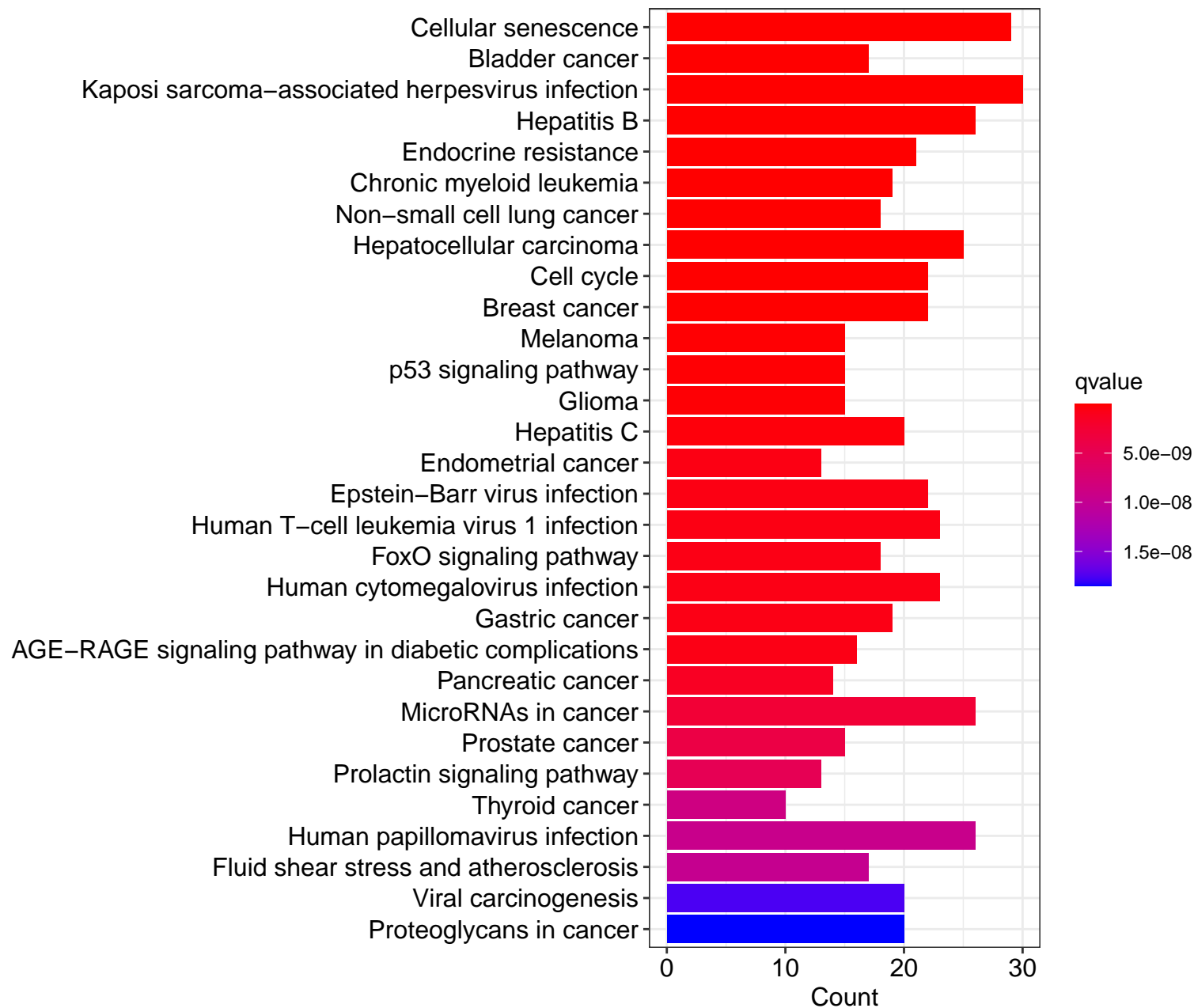

Supplement: Supplementary file 1 [file DataSheet_1.zip › 1.KEGG_GO/barplot.pdf]

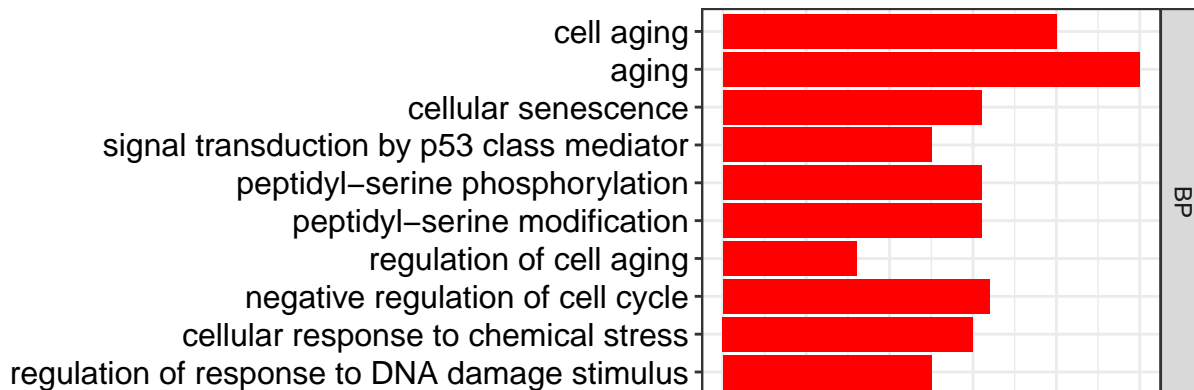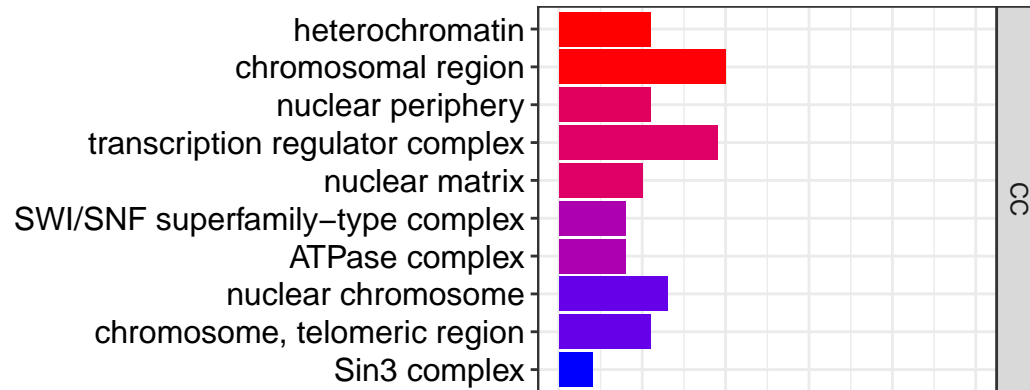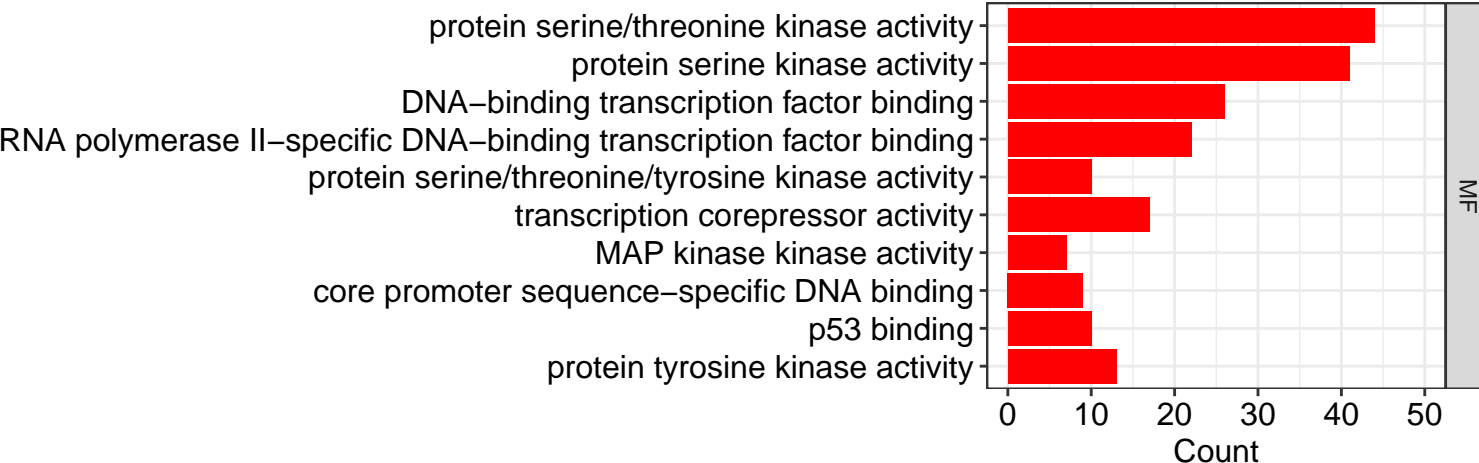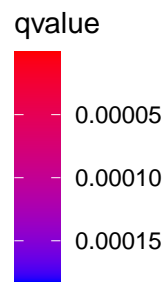

Supplement: Supplementary file 1 [file DataSheet_1.zip › 1.KEGG_GO/barplotgeo.pdf]

# NMF rank survey

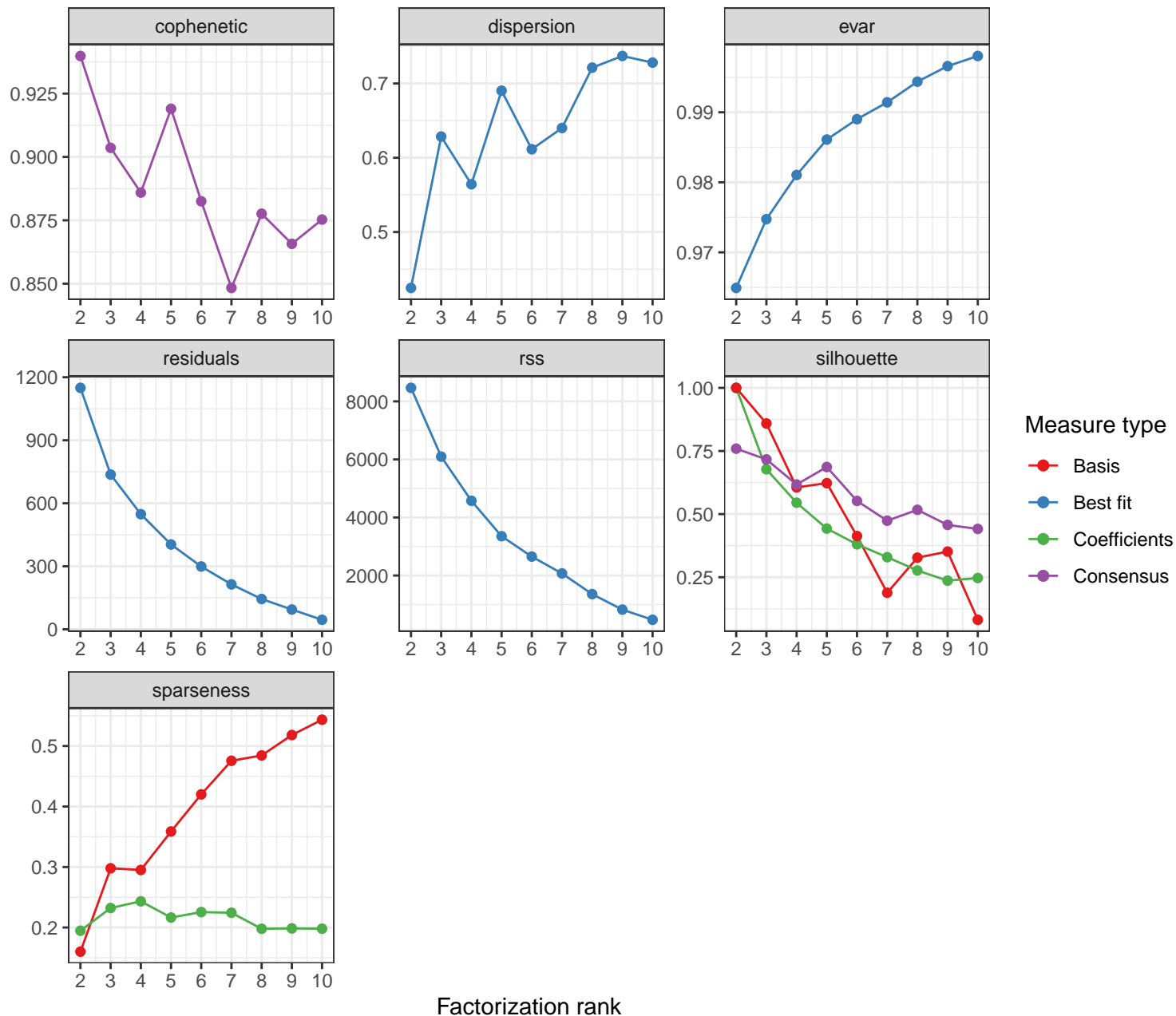

Supplement: Supplementary file 1 [file DataSheet_1.zip › 2.NMF/cophenetic.pdf]

Consensus matrix

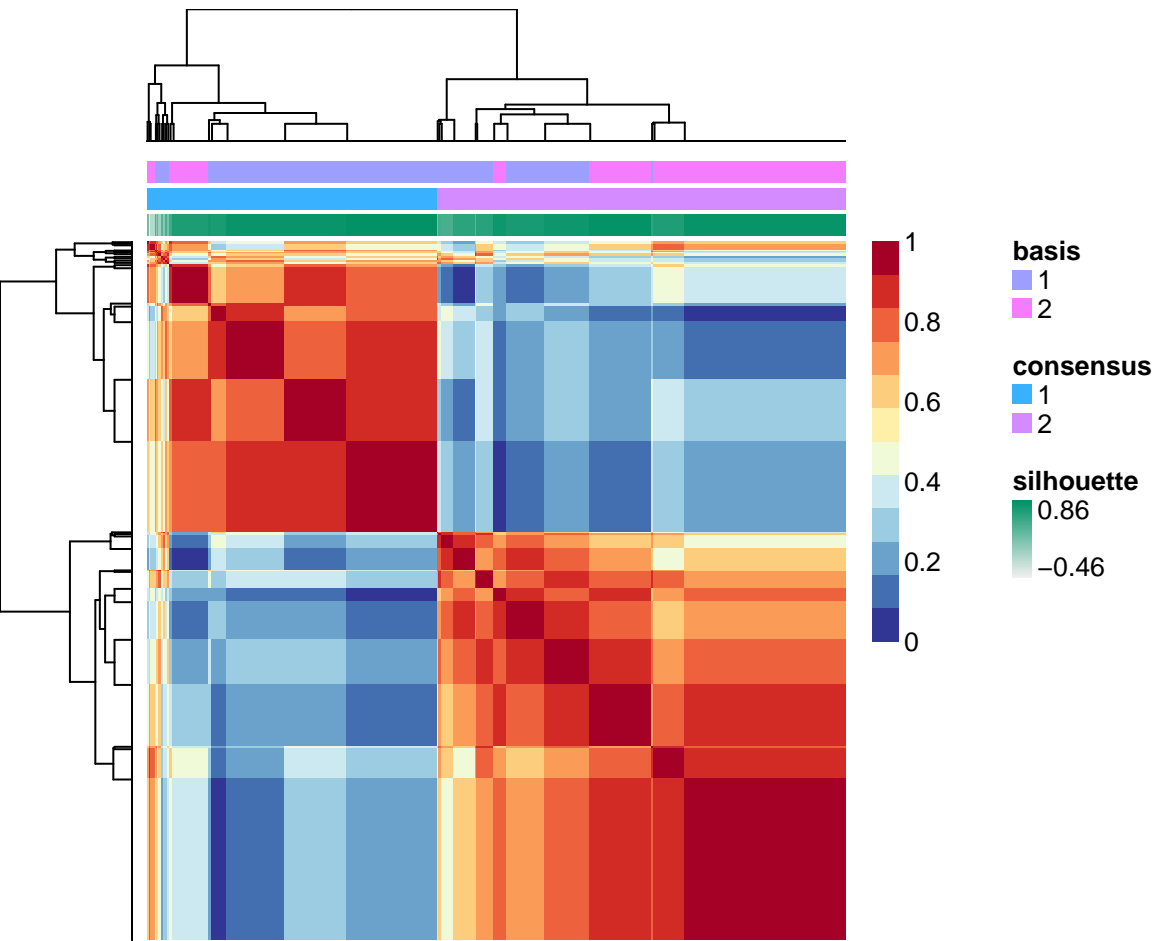

Supplement: Supplementary file 1 [file DataSheet_1.zip › 2.NMF/heatmap.pdf]

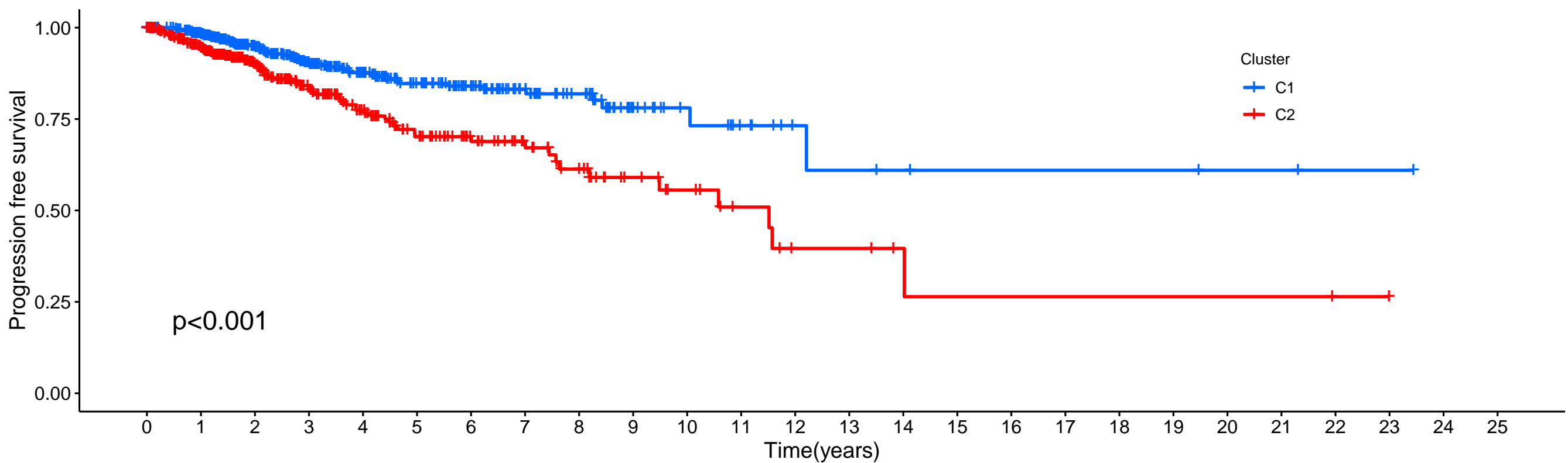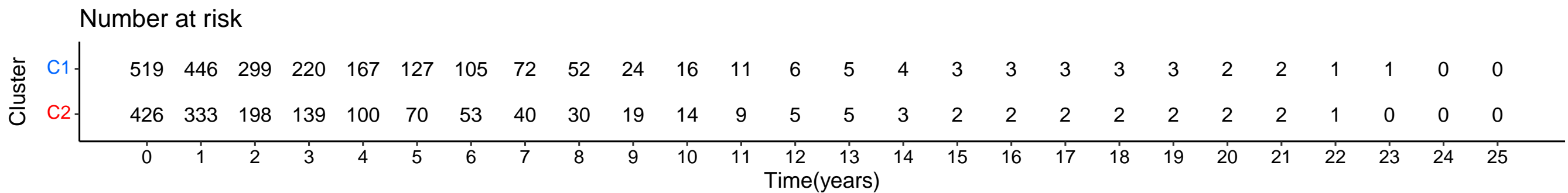

Supplement: Supplementary file 1 [file DataSheet_1.zip › 2.NMF/PFS.pdf]

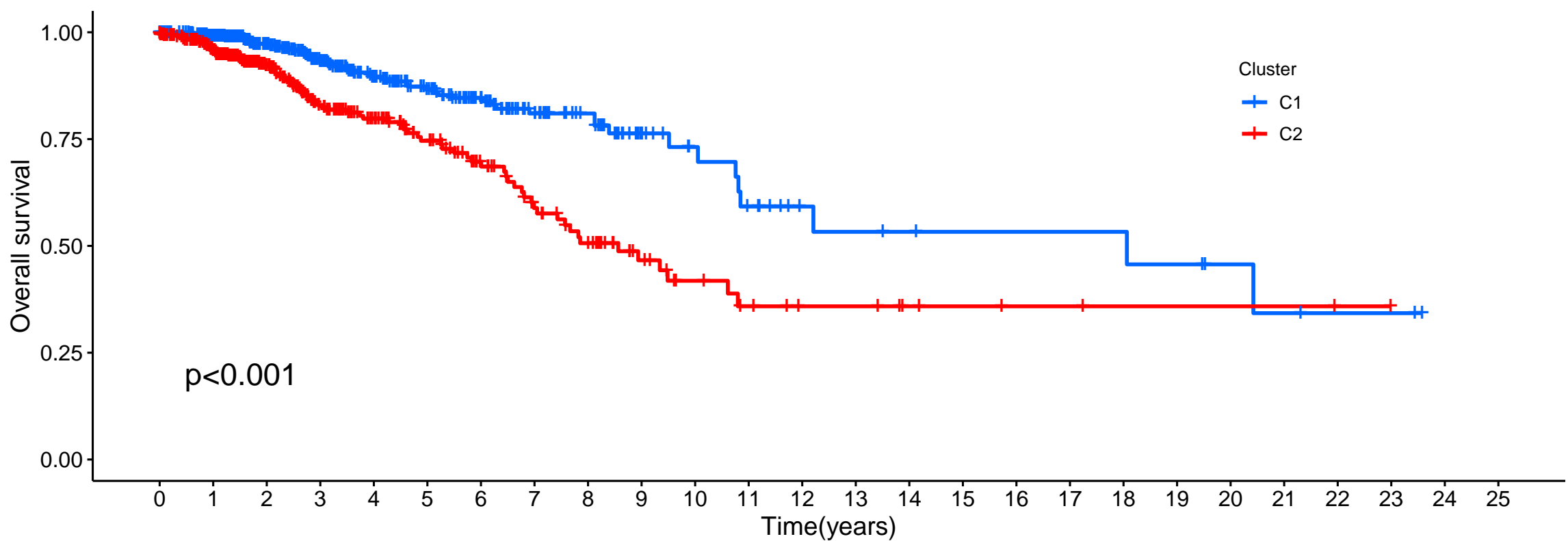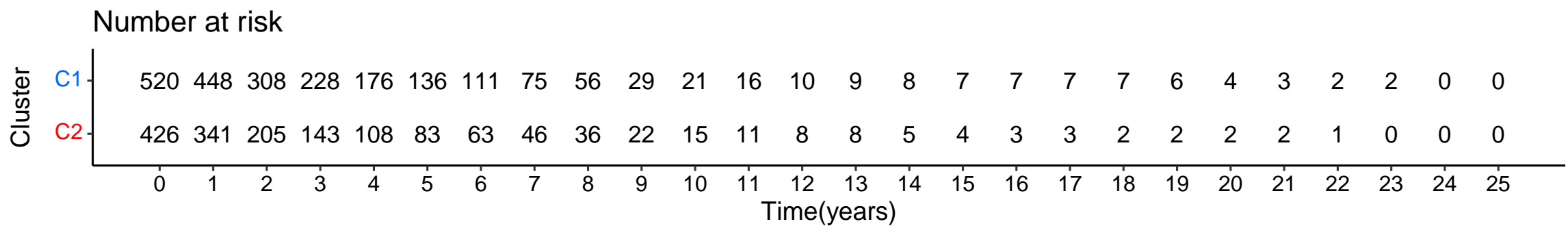

Supplement: Supplementary file 1 [file DataSheet_1.zip › 2.NMF/survival.pdf]

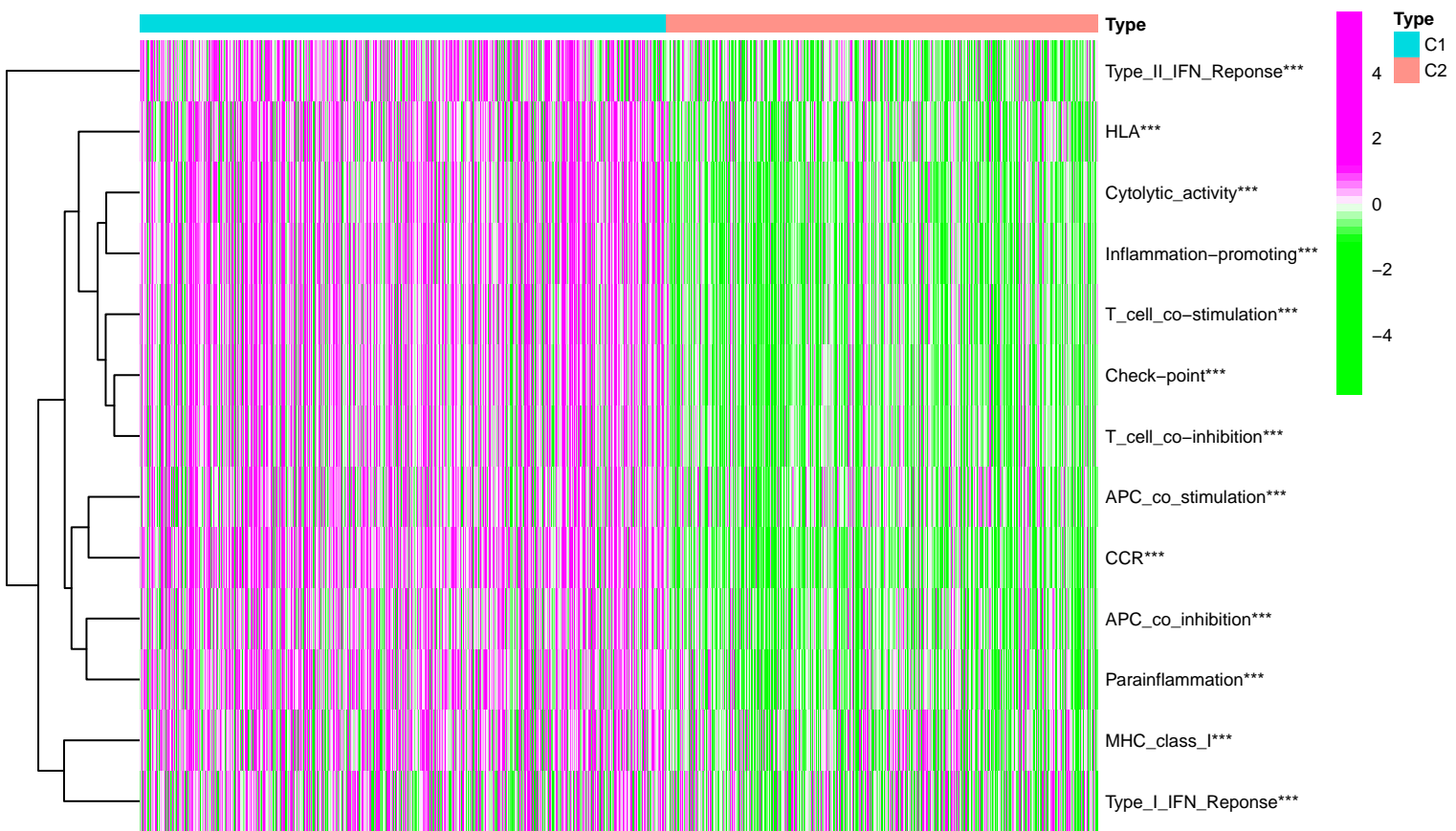

Supplement: Supplementary file 1 [file DataSheet_1.zip › 4.SSGSEA/heatmap.pdf]

Cluster

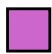

C1

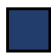

C2

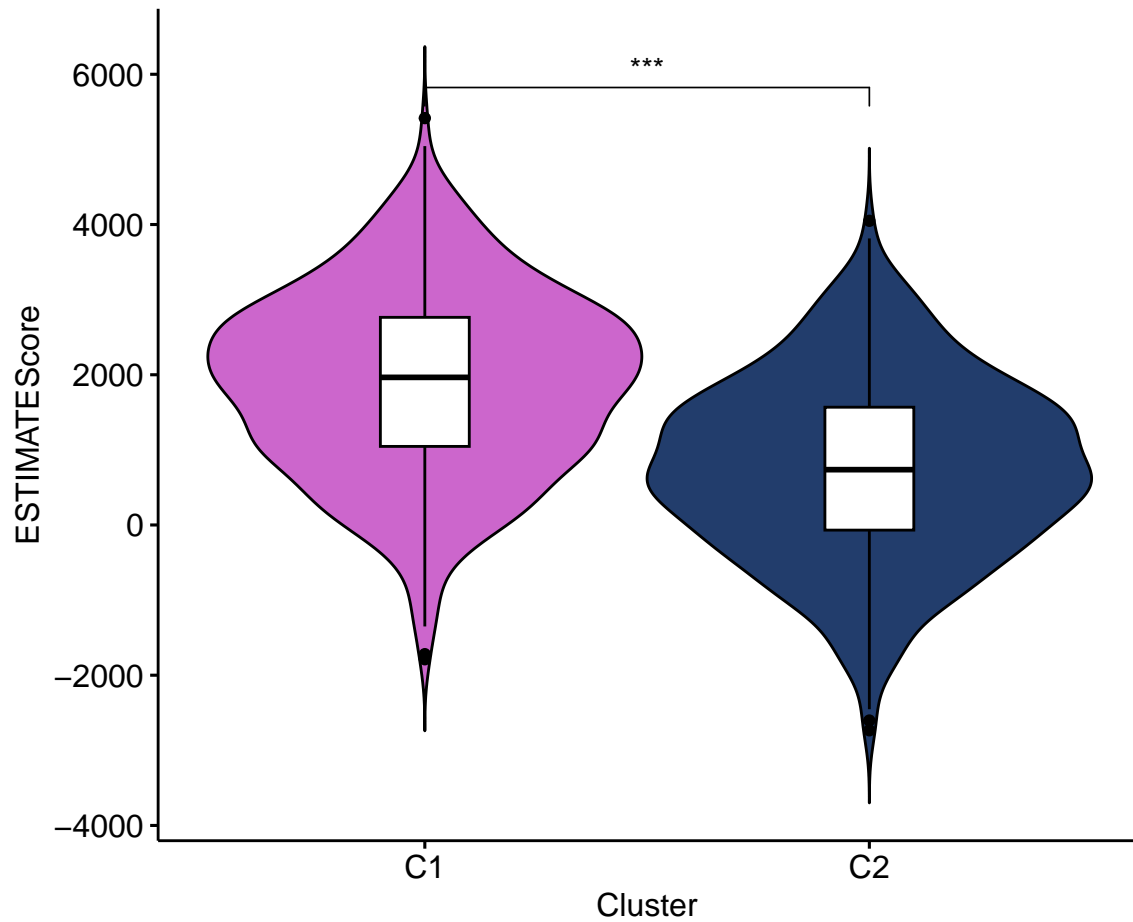

Supplement: Supplementary file 2 [file DataSheet_2.zip › 3.DAFEN/vioplot.ESTIMATEScore.pdf]

Cluster

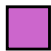

C1

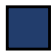

C2

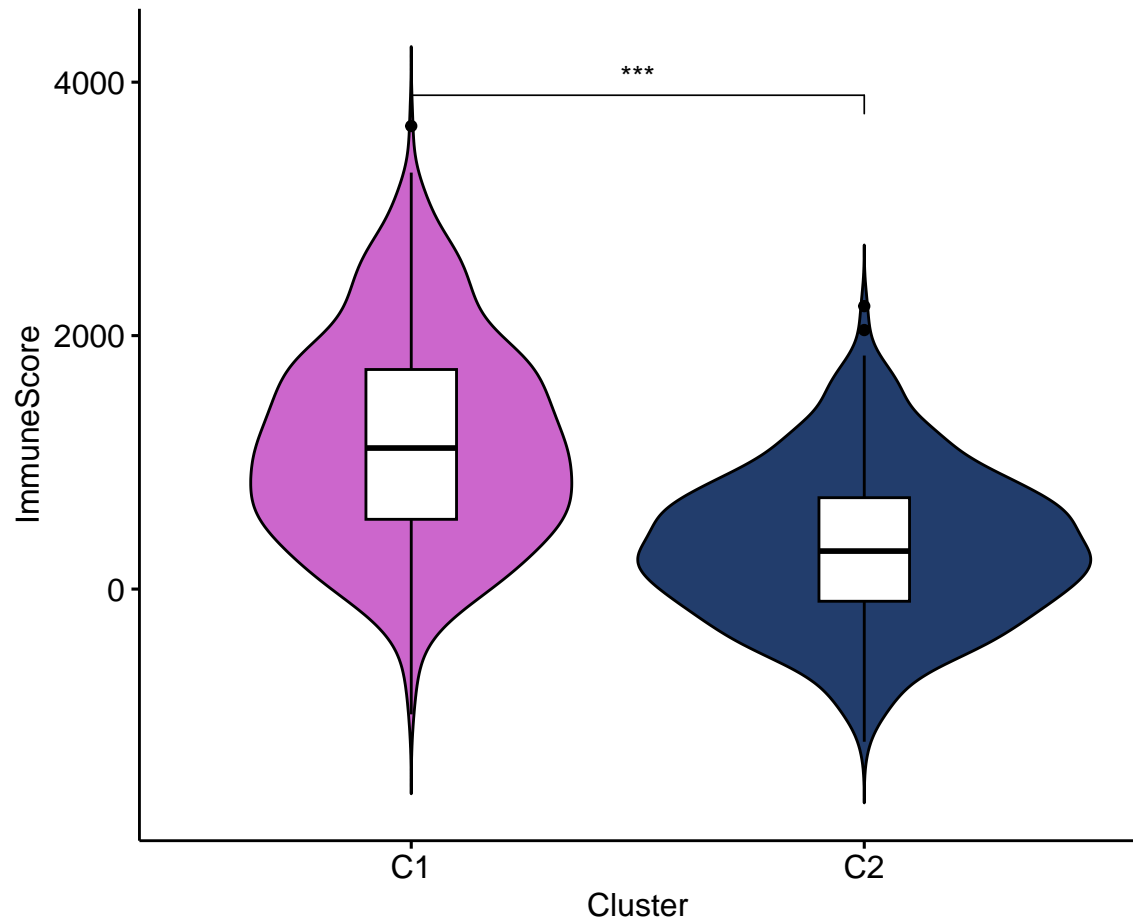

Supplement: Supplementary file 2 [file DataSheet_2.zip › 3.DAFEN/vioplot.ImmuneScore.pdf]

Cluster

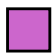

C1

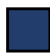

C2

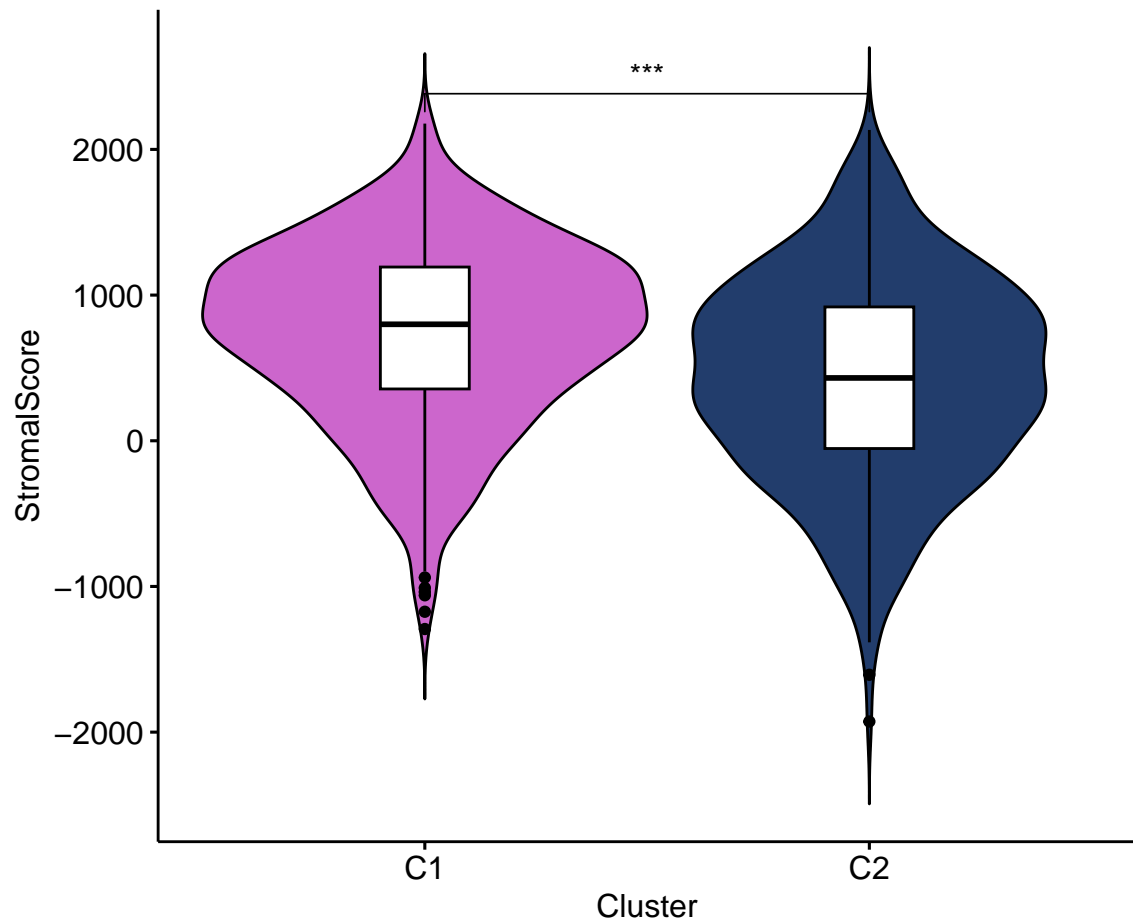

Supplement: Supplementary file 2 [file DataSheet_2.zip › 3.DAFEN/vioplot.StromalScore.pdf]

Cluster

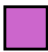

C1

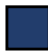

C2

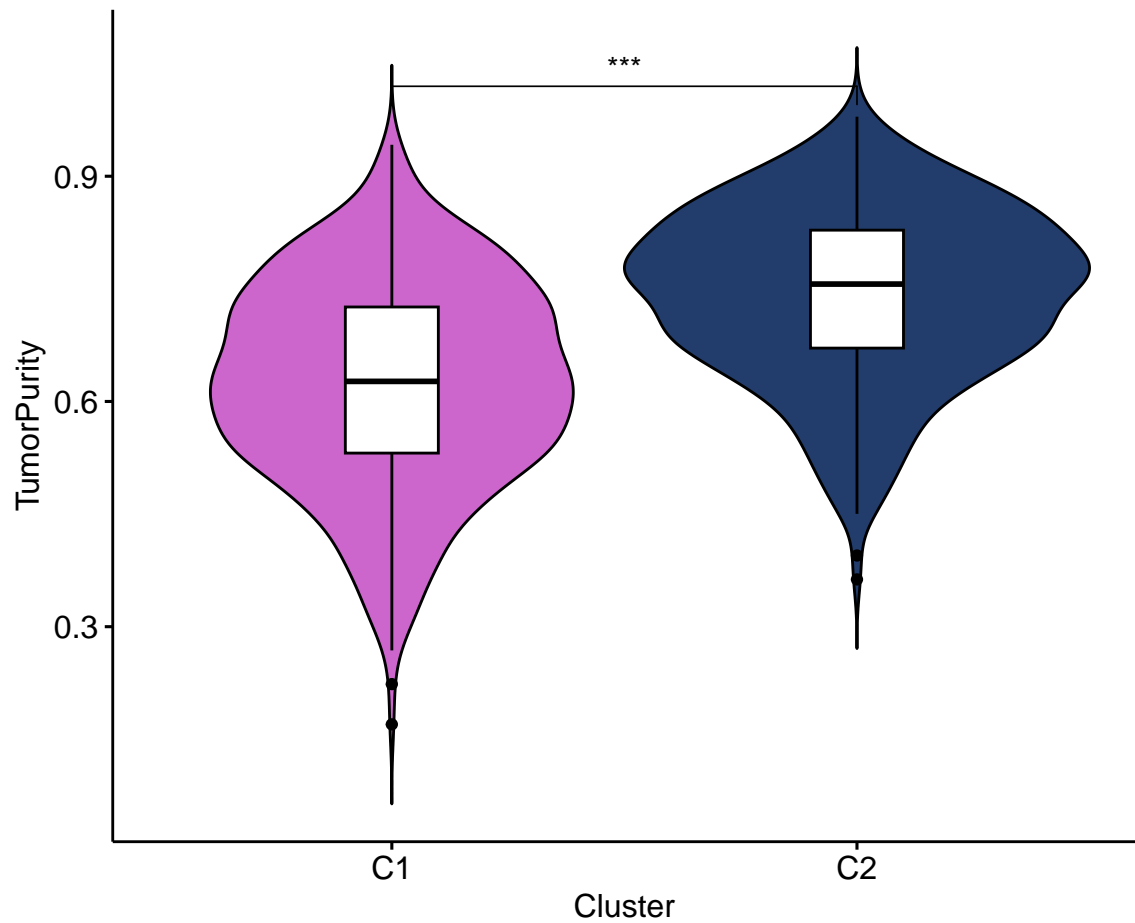

Supplement: Supplementary file 2 [file DataSheet_2.zip › 3.DAFEN/vioplot.TumorPurity.pdf]

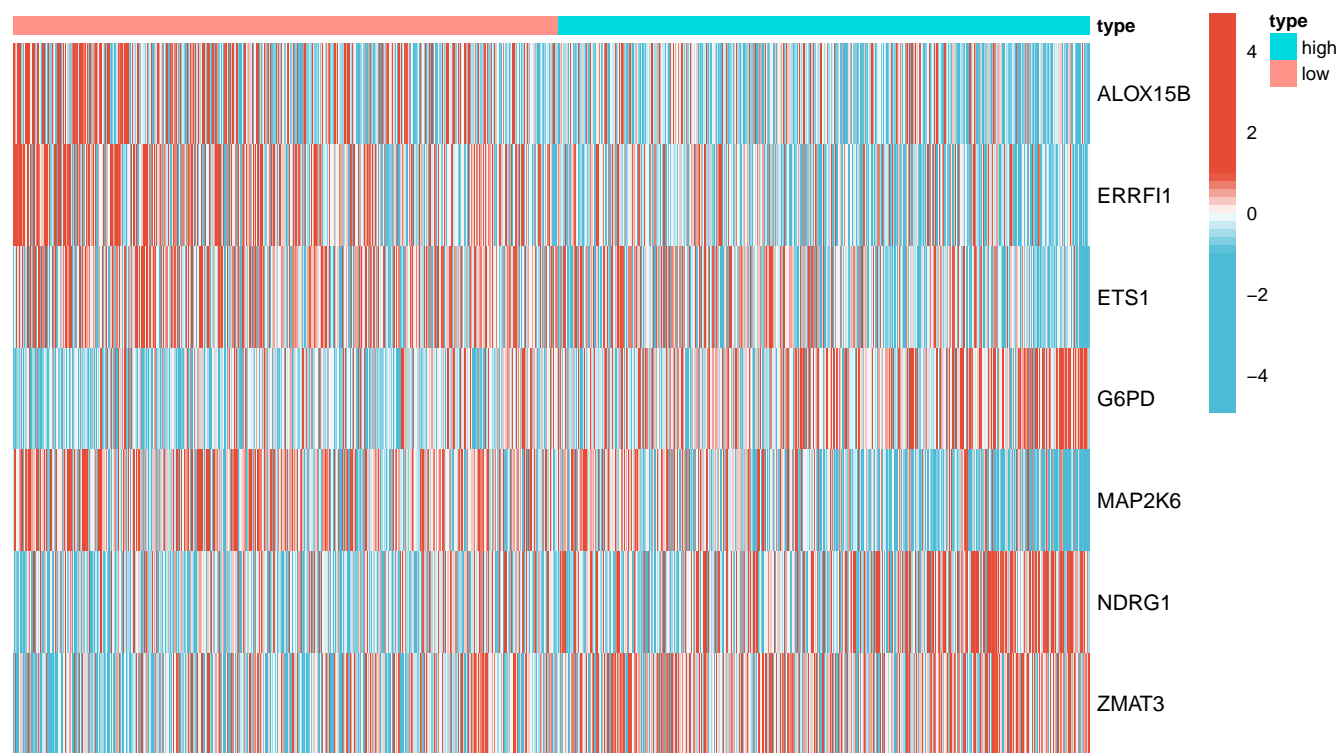

Supplement: Supplementary file 3 [file DataSheet_3.zip › 5.model/allheatmap.pdf]

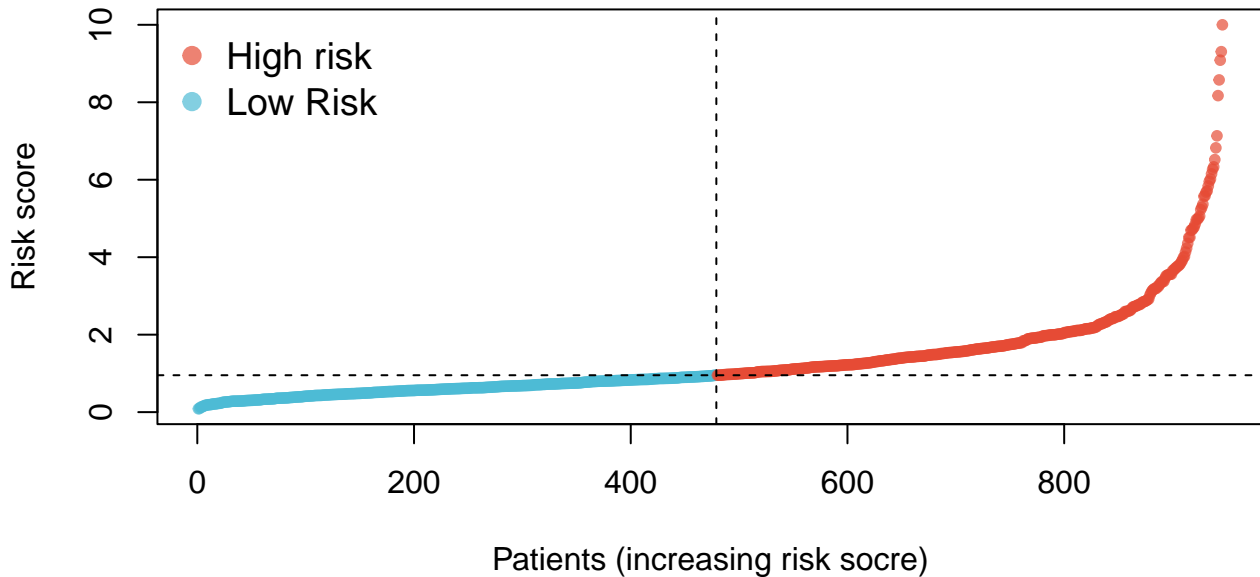

Supplement: Supplementary file 3 [file DataSheet_3.zip › 5.model/allriskScore.pdf]

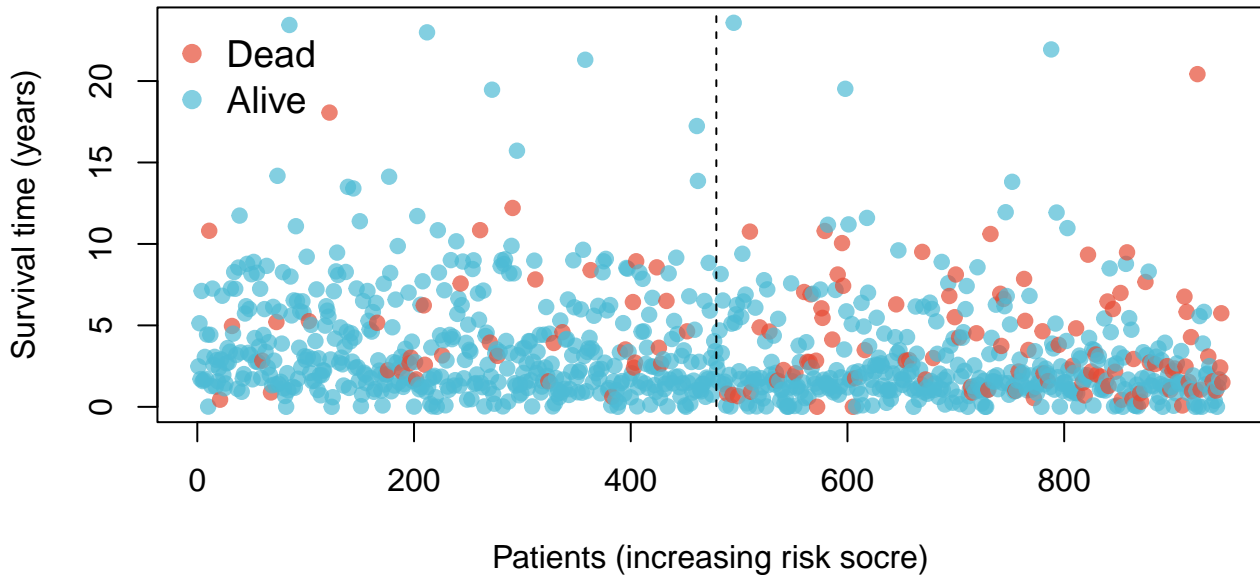

Supplement: Supplementary file 3 [file DataSheet_3.zip › 5.model/allsurvStat.pdf]

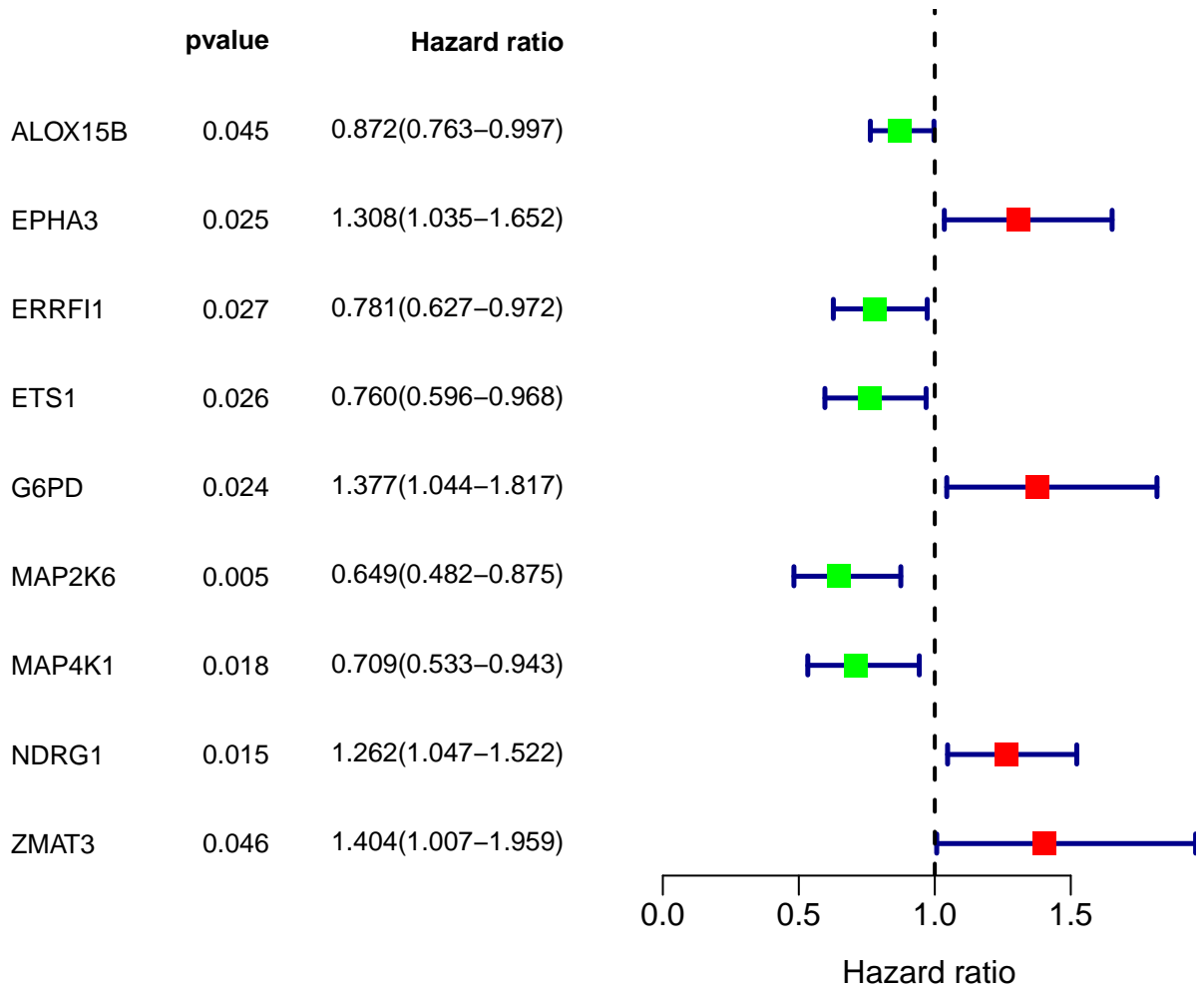

Supplement: Supplementary file 3 [file DataSheet_3.zip › 5.model/forest.pdf]

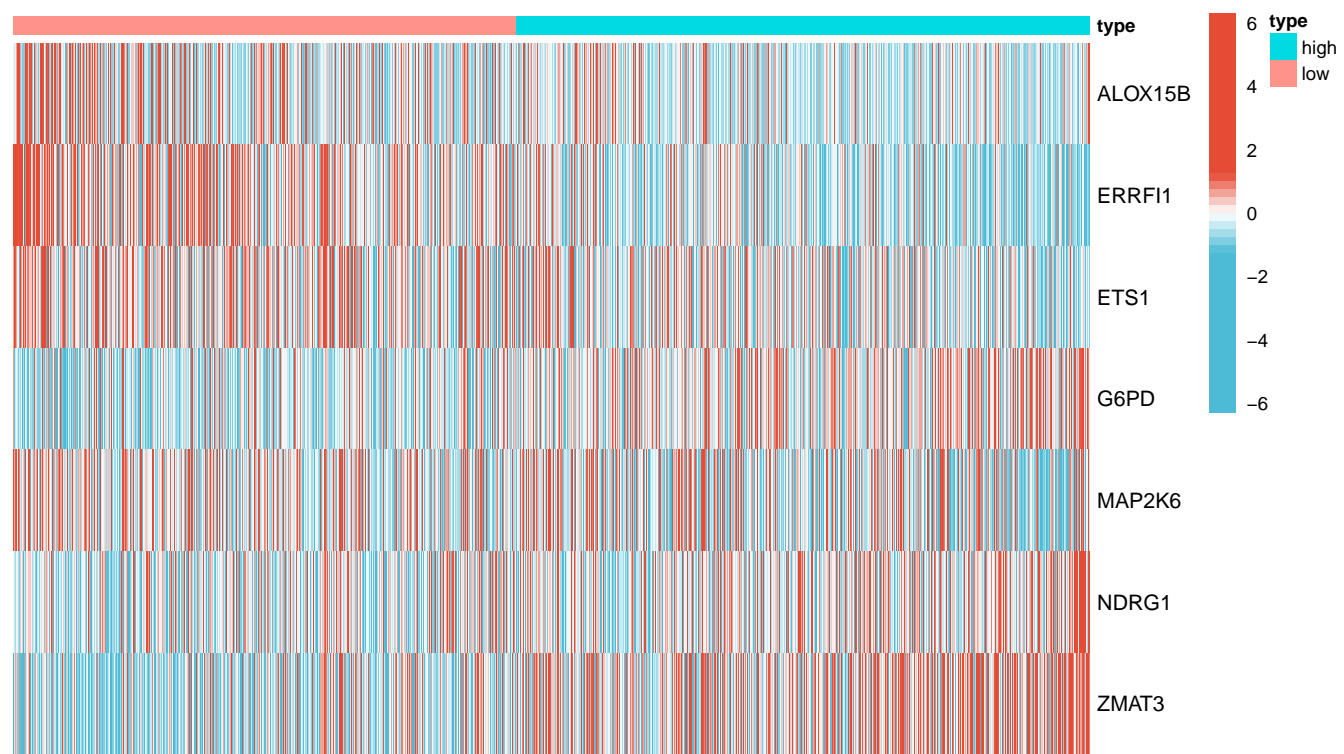

Supplement: Supplementary file 3 [file DataSheet_3.zip › 5.model/GEOheatmap.pdf]

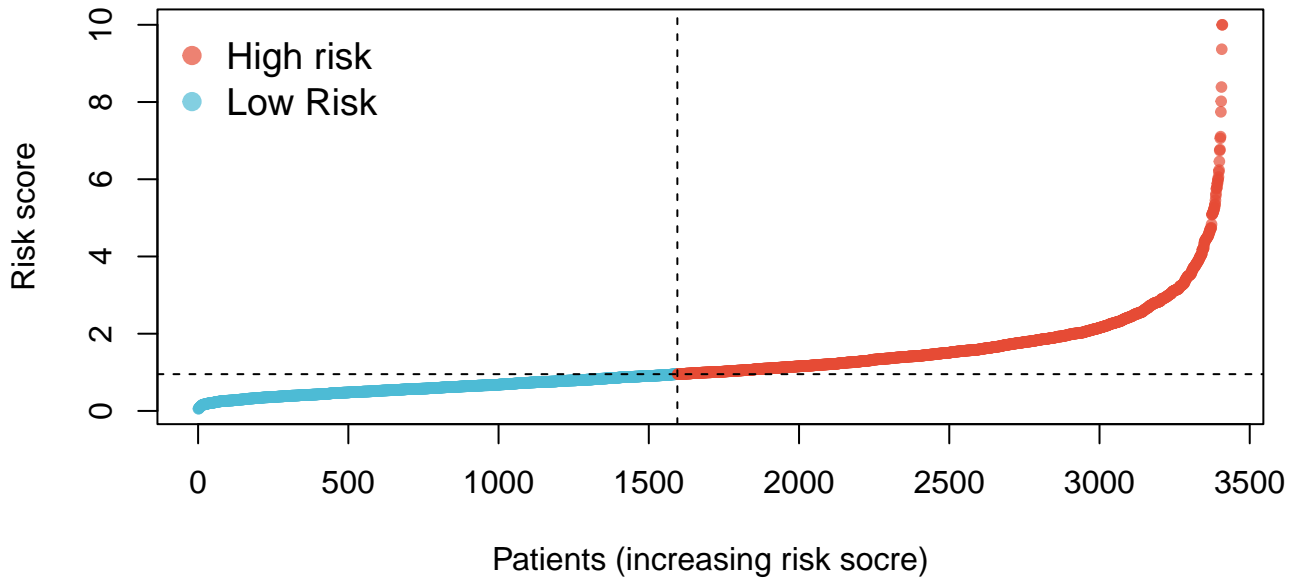

Supplement: Supplementary file 3 [file DataSheet_3.zip › 5.model/GEOriskScore.pdf]

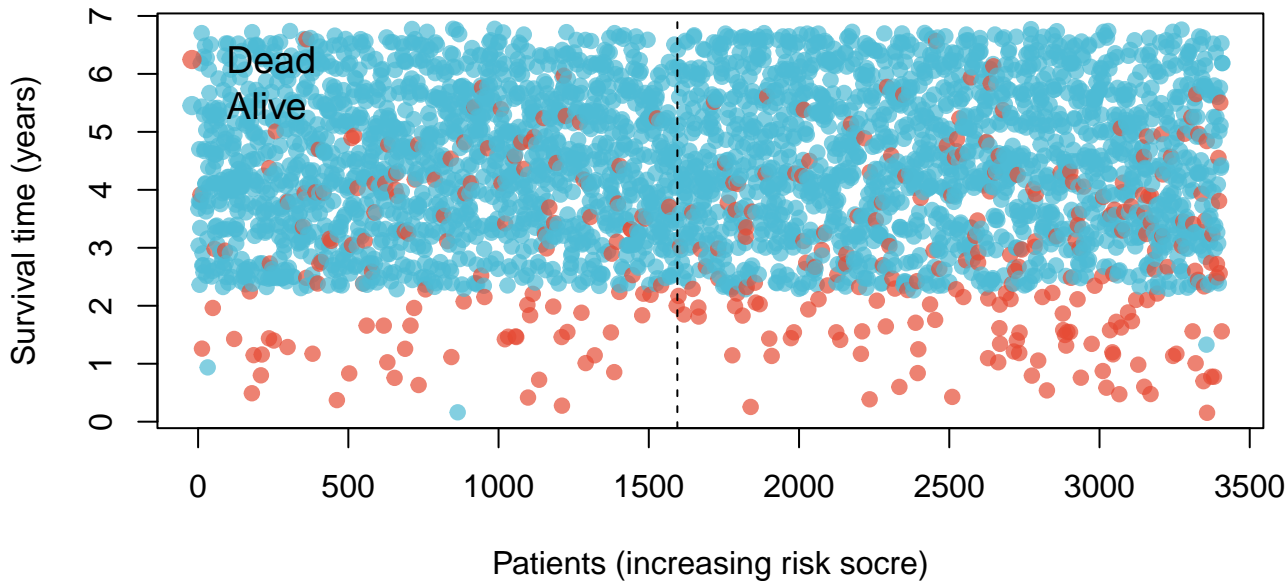

Supplement: Supplementary file 3 [file DataSheet_3.zip › 5.model/GEOsurvStat.pdf]

Partial Likelihood Deviance

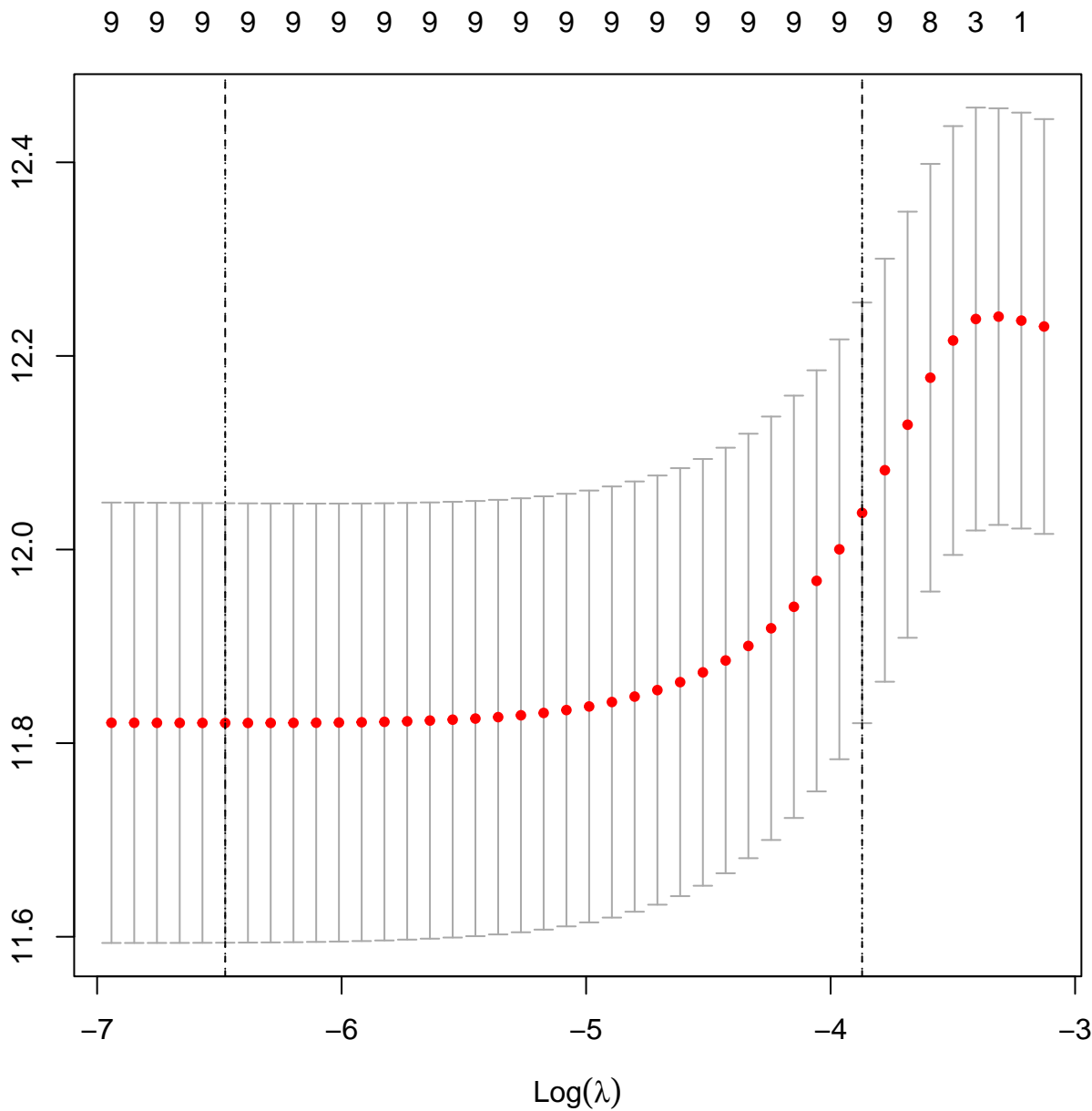

Supplement: Supplementary file 3 [file DataSheet_3.zip › 5.model/lasso.cvfit.pdf]

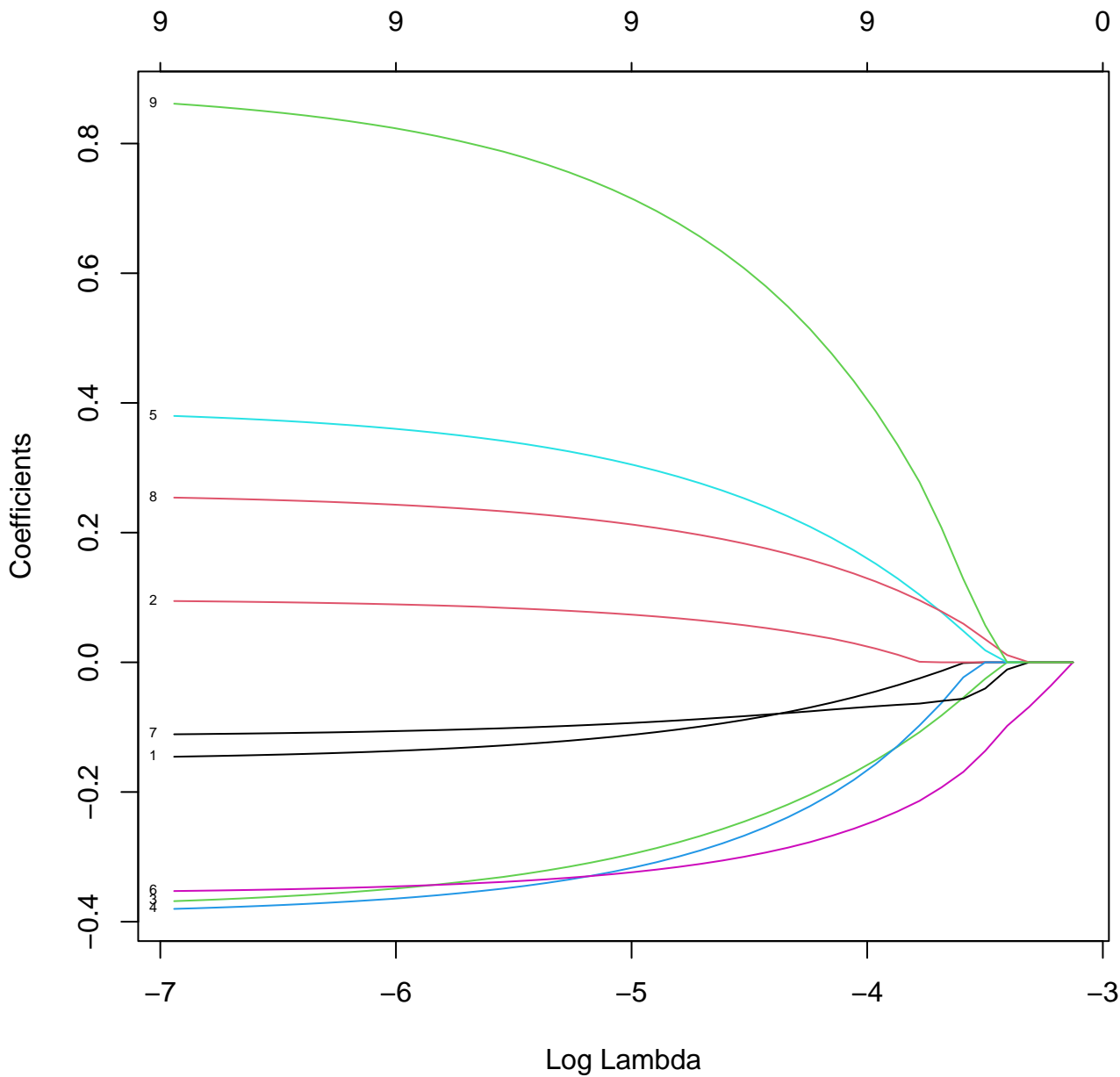

Supplement: Supplementary file 3 [file DataSheet_3.zip › 5.model/lasso.lambda.pdf]

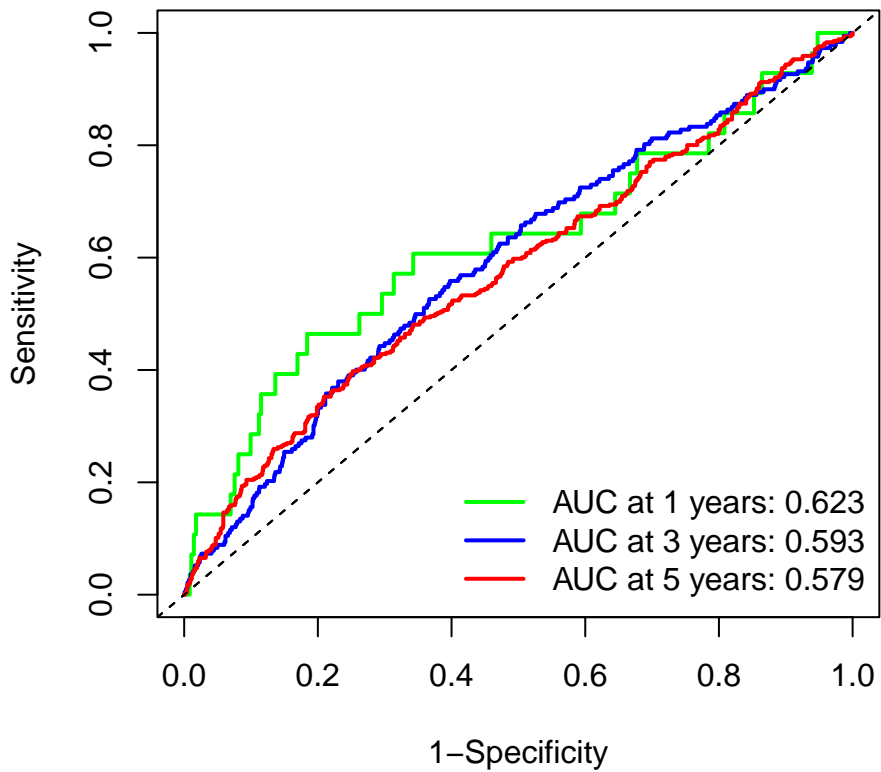

Supplement: Supplementary file 3 [file DataSheet_3.zip › 5.model/ROC.GEO.pdf]

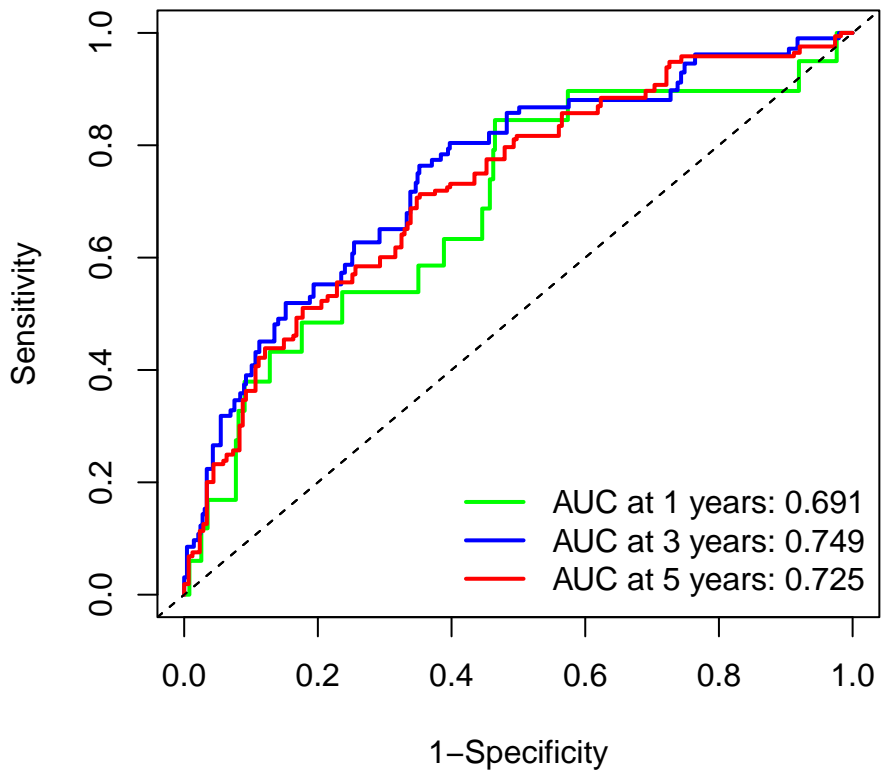

Supplement: Supplementary file 3 [file DataSheet_3.zip › 5.model/ROC.TCGAall.pdf]

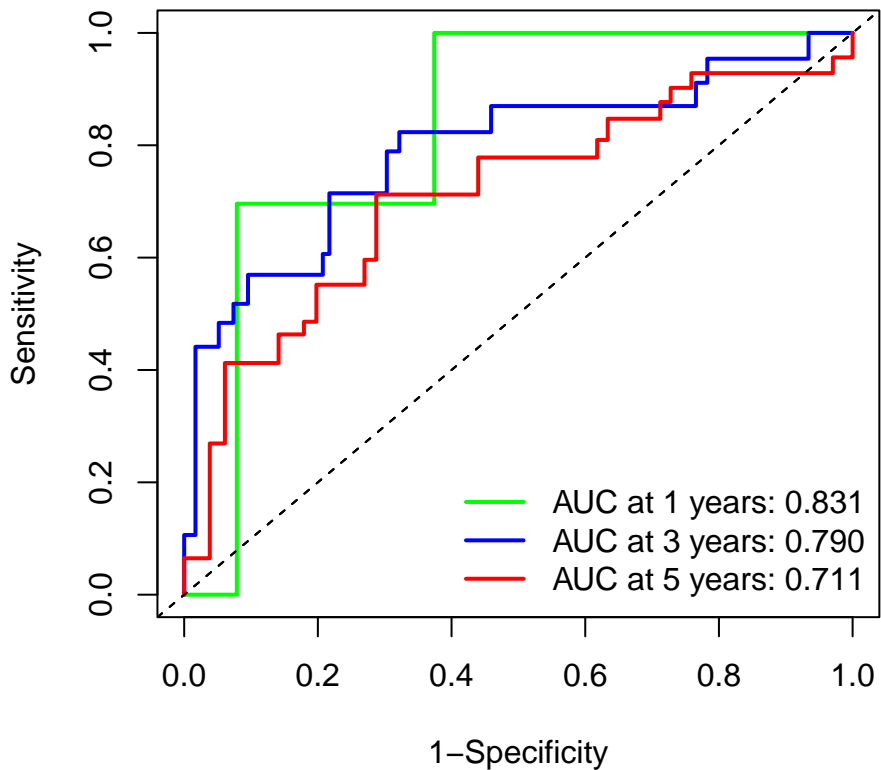

Supplement: Supplementary file 3 [file DataSheet_3.zip › 5.model/ROC.TCGAtest.pdf]

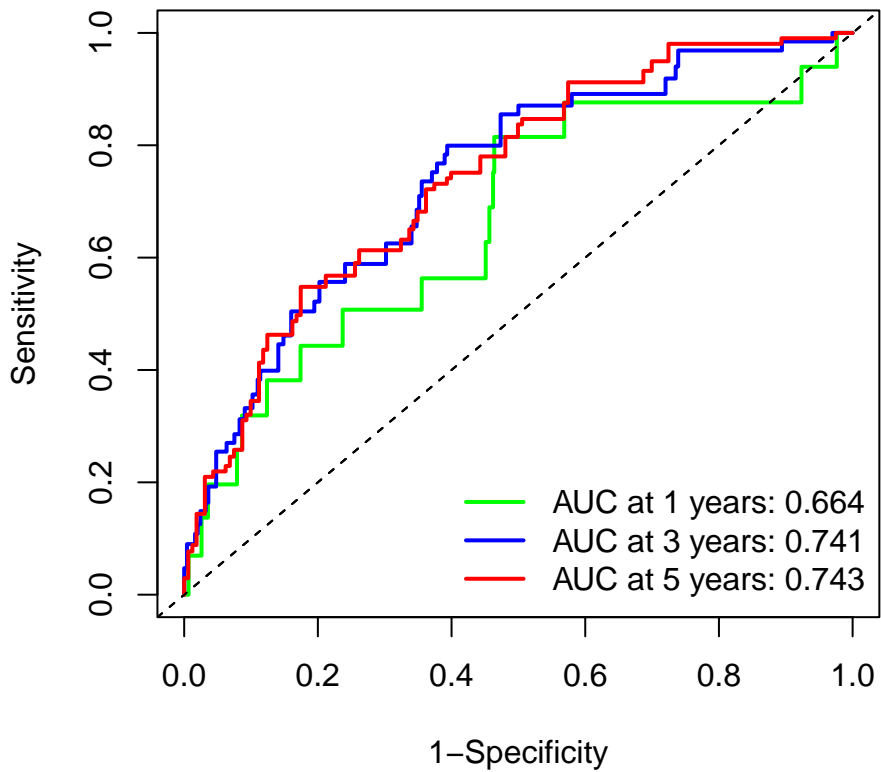

Supplement: Supplementary file 3 [file DataSheet_3.zip › 5.model/ROC.TCGAtrain.pdf]

Survival probability

Risk High risk Low risk

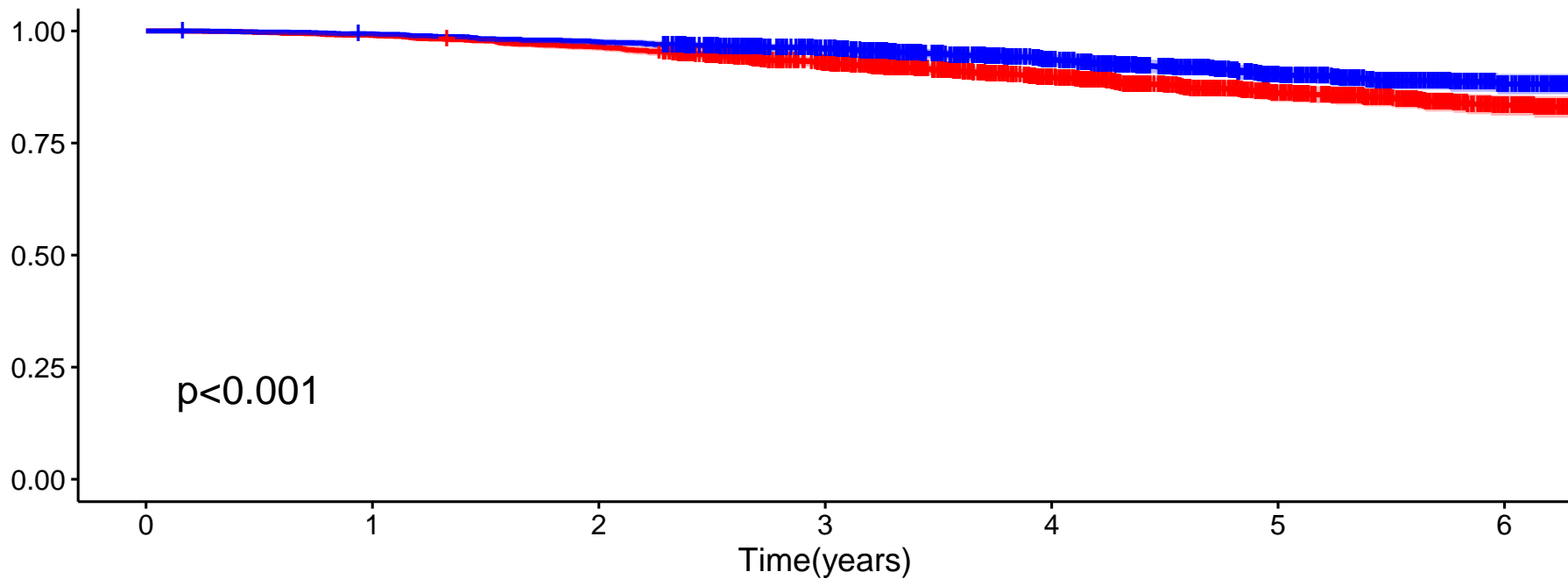

Risk

High risk  
Low risk

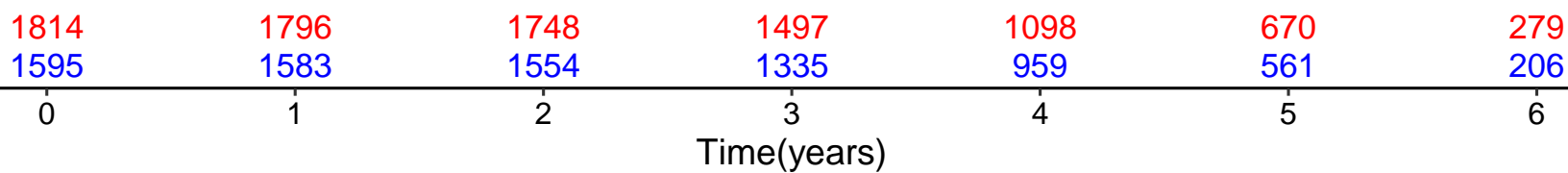

Supplement: Supplementary file 3 [file DataSheet_3.zip › 5.model/surv.GEO.pdf]

Survival probability

Risk High risk Low risk

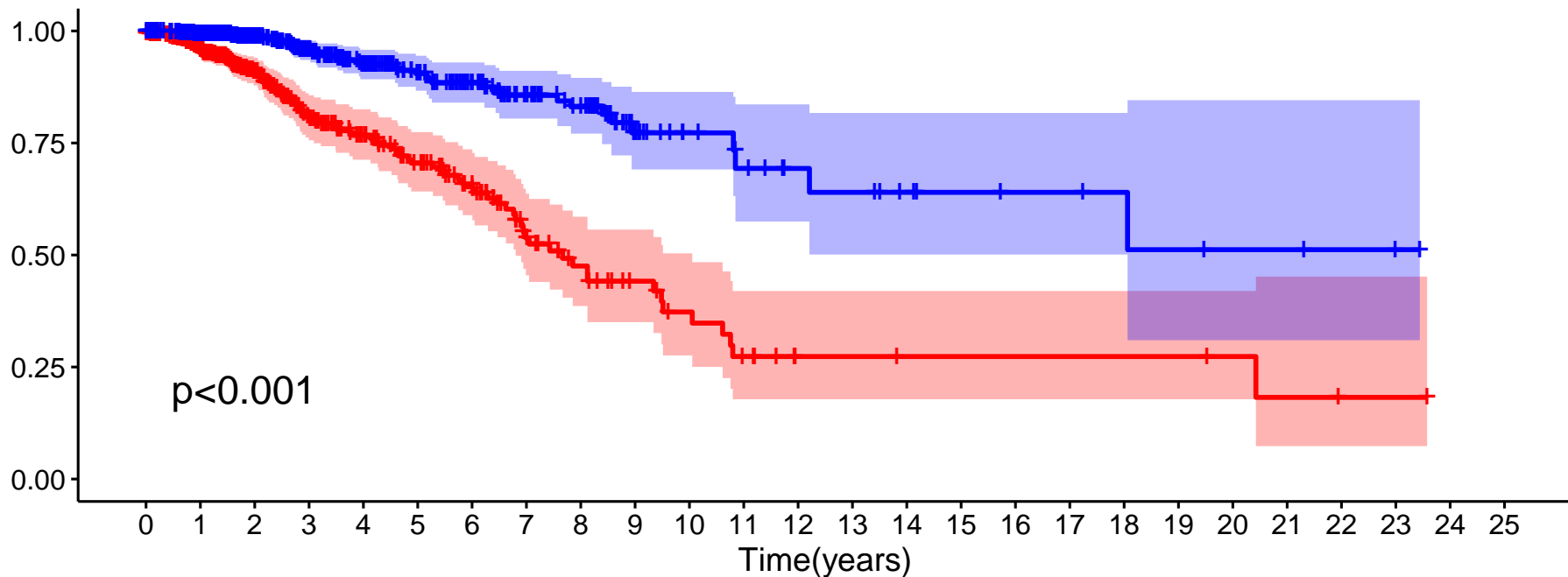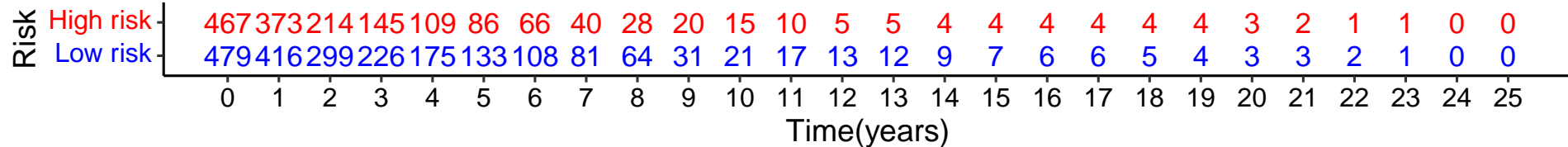

Supplement: Supplementary file 3 [file DataSheet_3.zip › 5.model/surv.TCGAall.pdf]

Survival probability

Risk High risk Low risk

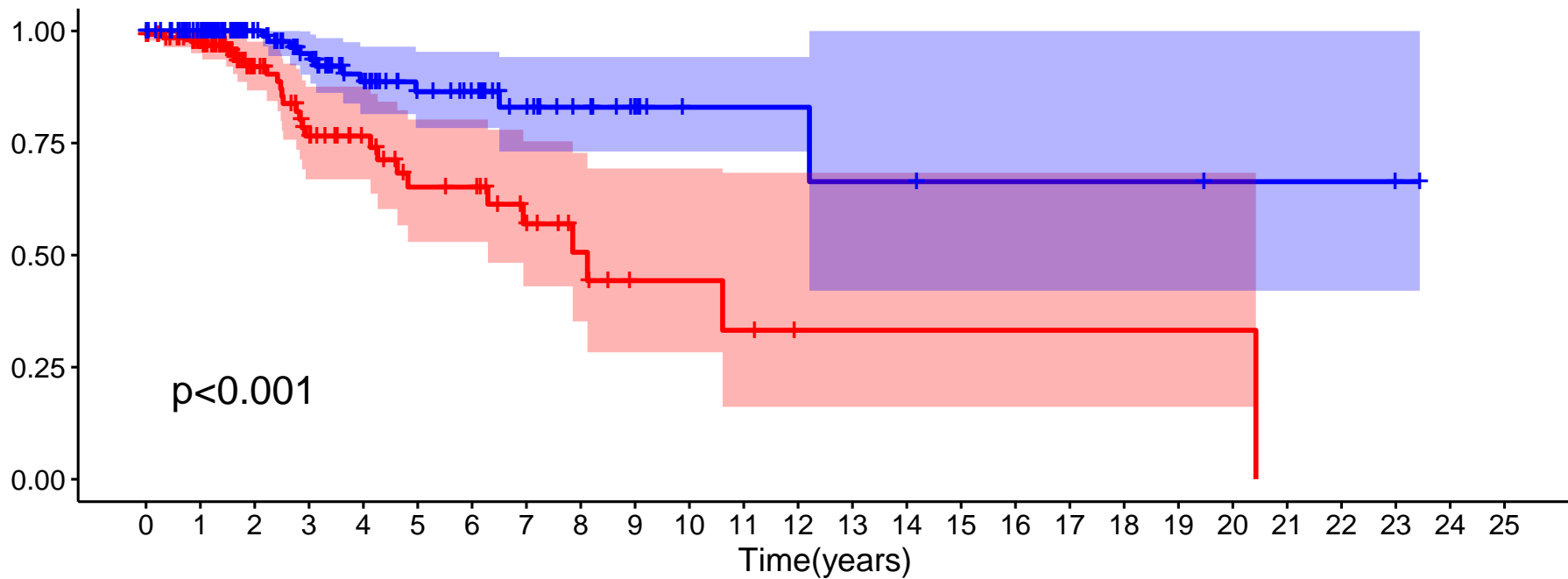

Risk

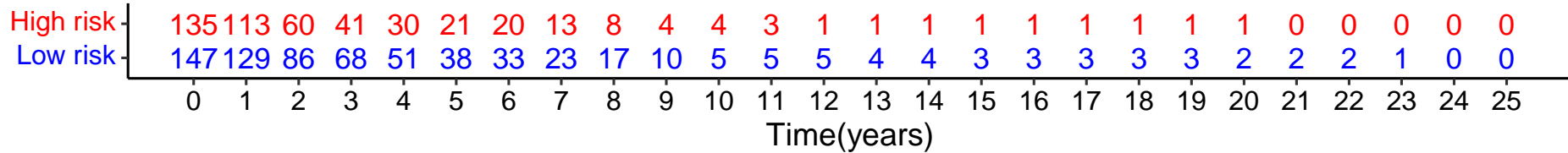

Supplement: Supplementary file 3 [file DataSheet_3.zip › 5.model/surv.TCGAtest.pdf]

Survival probability

Risk High risk Low risk

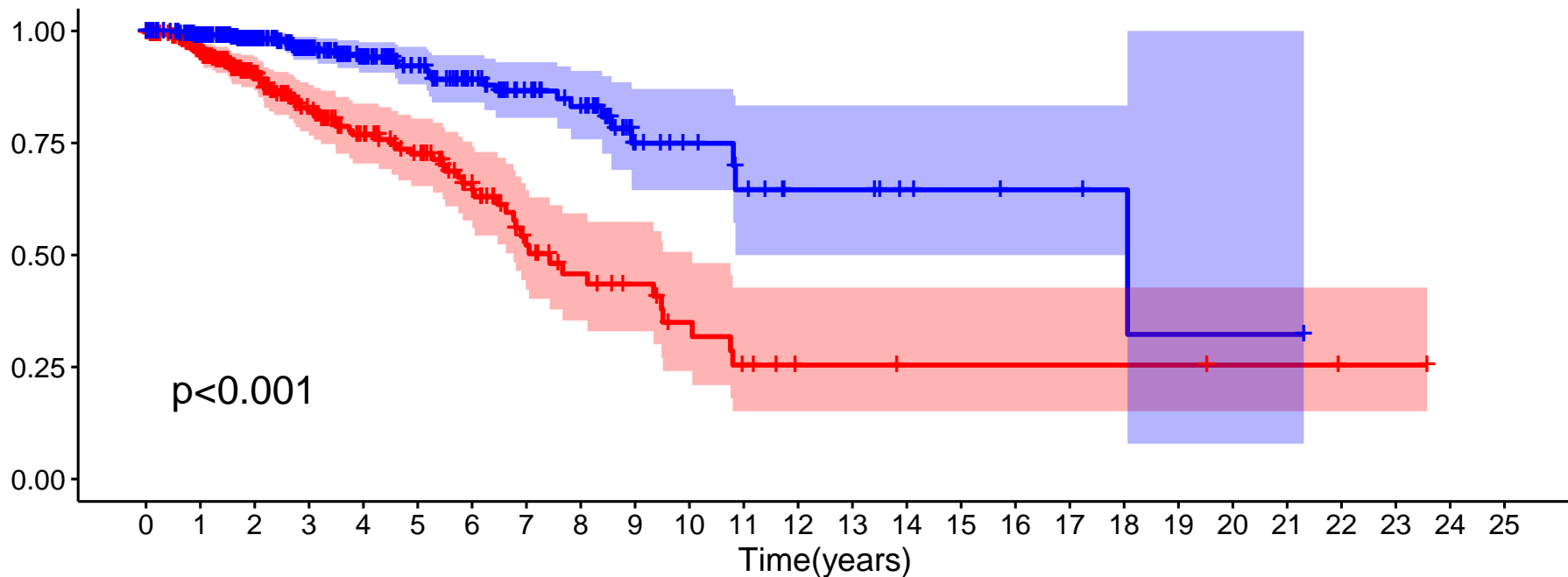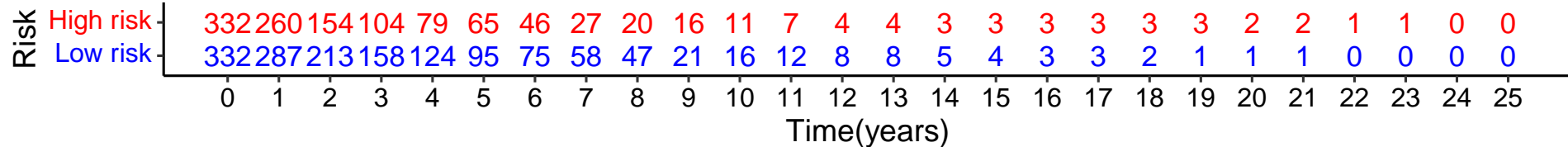

Supplement: Supplementary file 3 [file DataSheet_3.zip › 5.model/surv.TCGAtrain.pdf]

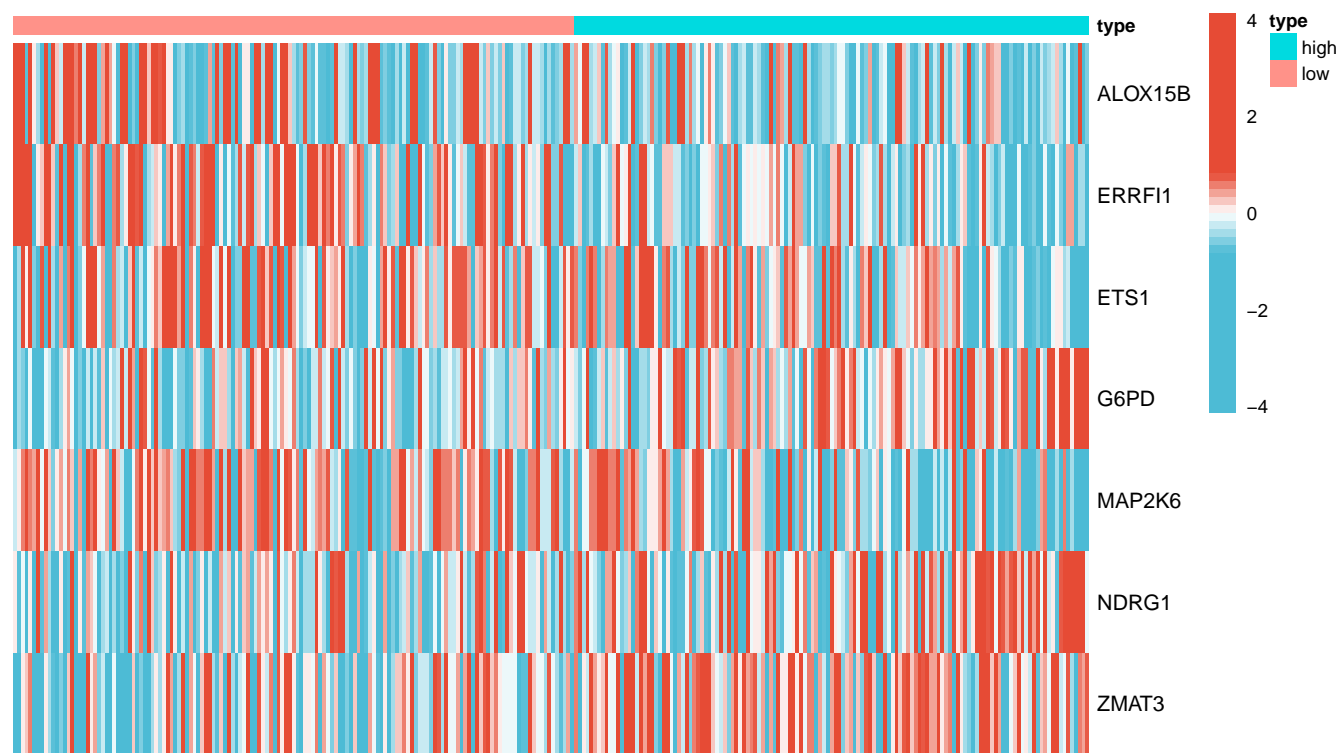

Supplement: Supplementary file 3 [file DataSheet_3.zip › 5.model/testheatmap.pdf]

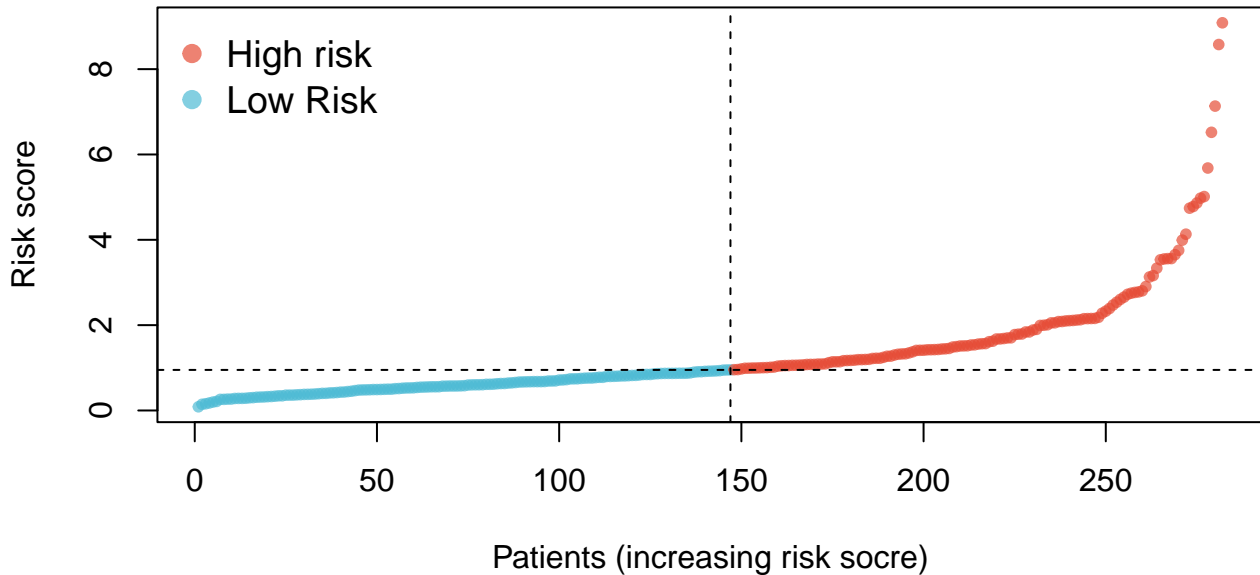

Supplement: Supplementary file 3 [file DataSheet_3.zip › 5.model/testriskScore.pdf]

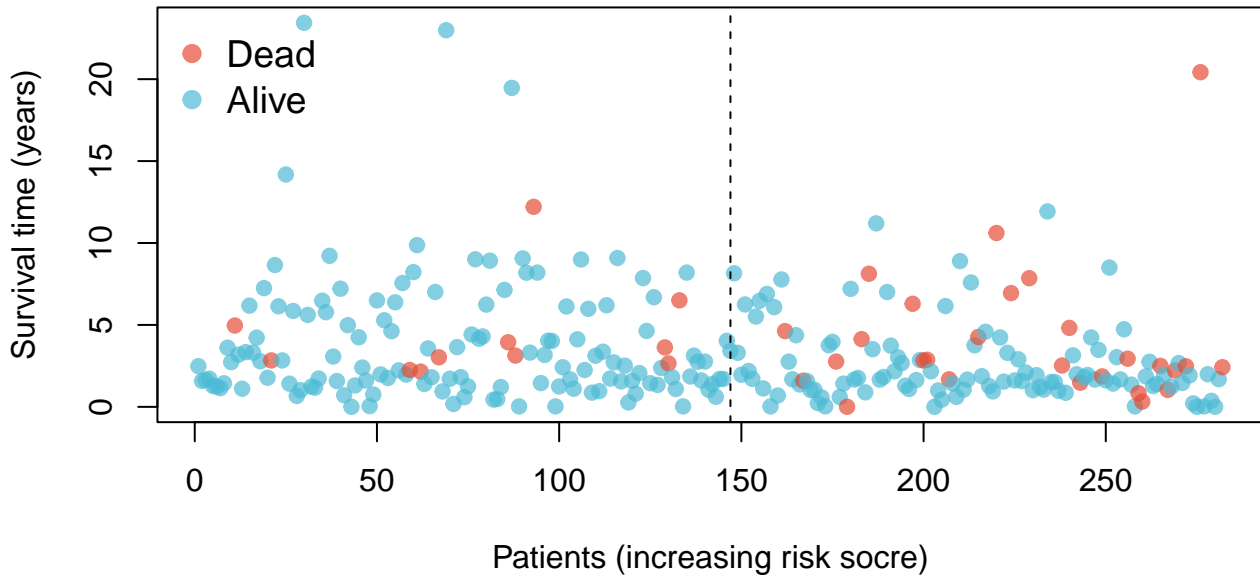

Supplement: Supplementary file 3 [file DataSheet_3.zip › 5.model/testsurvStat.pdf]

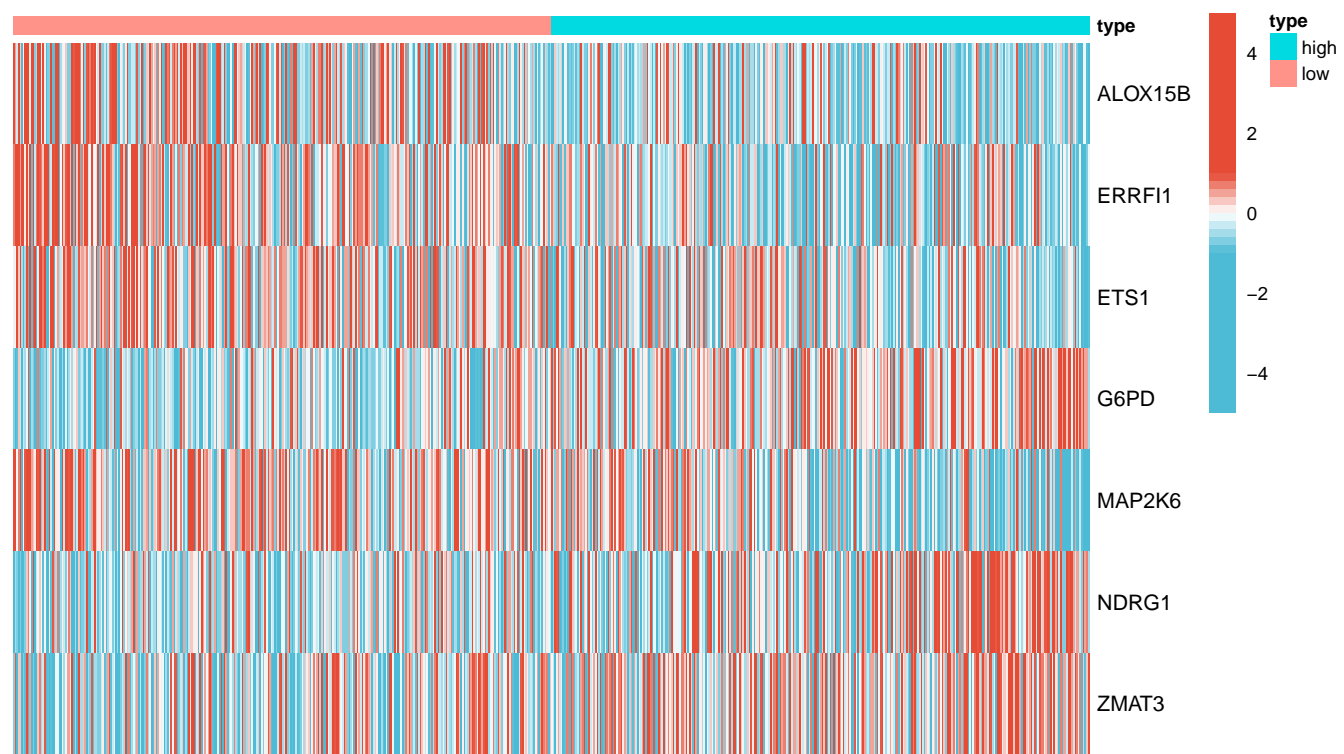

Supplement: Supplementary file 3 [file DataSheet_3.zip › 5.model/trainheatmap.pdf]

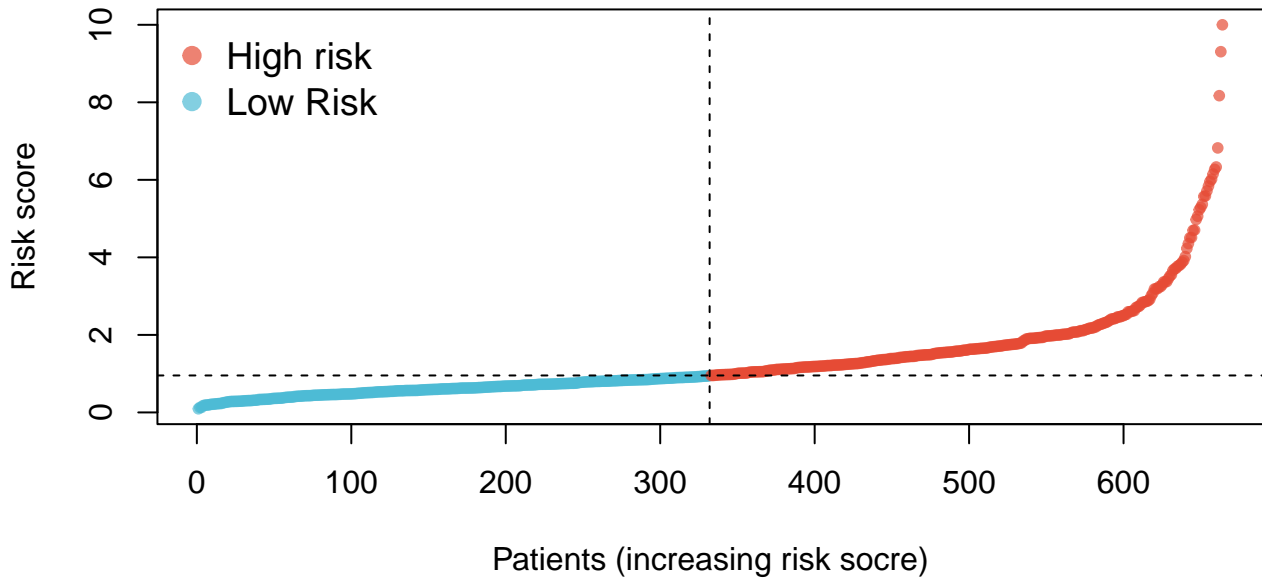

Supplement: Supplementary file 3 [file DataSheet_3.zip › 5.model/trainriskScore.pdf]

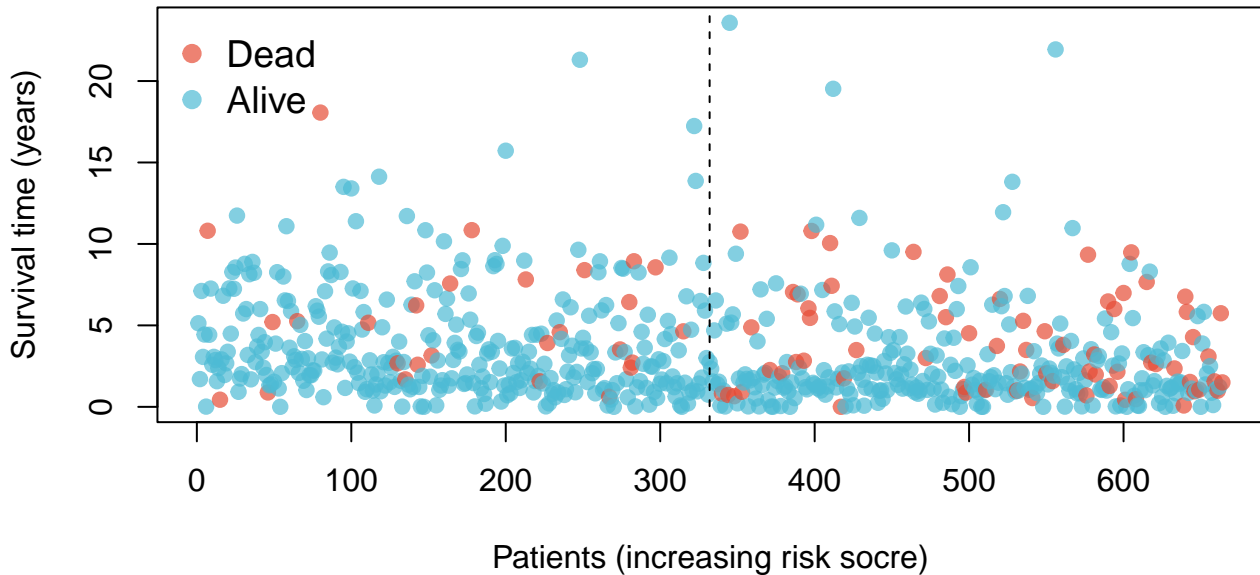

Supplement: Supplementary file 3 [file DataSheet_3.zip › 5.model/trainsurvStat.pdf]

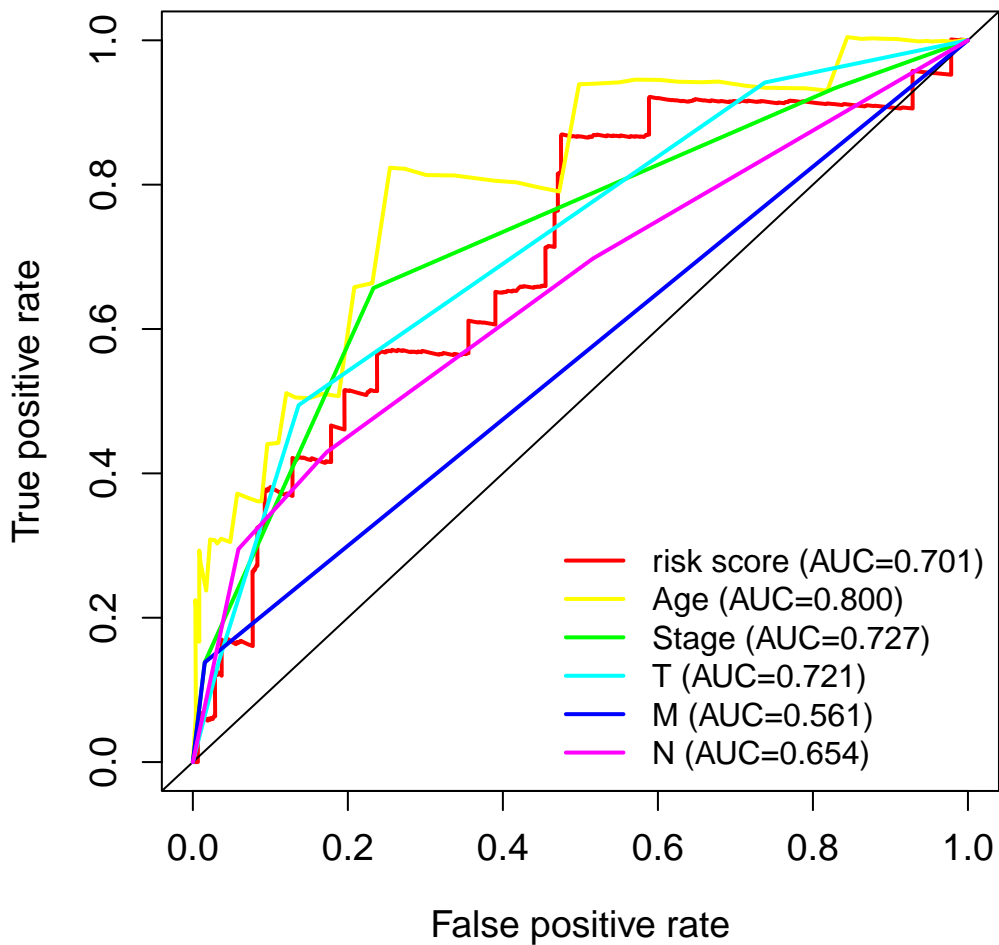

Supplement: Supplementary file 3 [file DataSheet_3.zip › 6.cli_roc/cliROC.pdf]

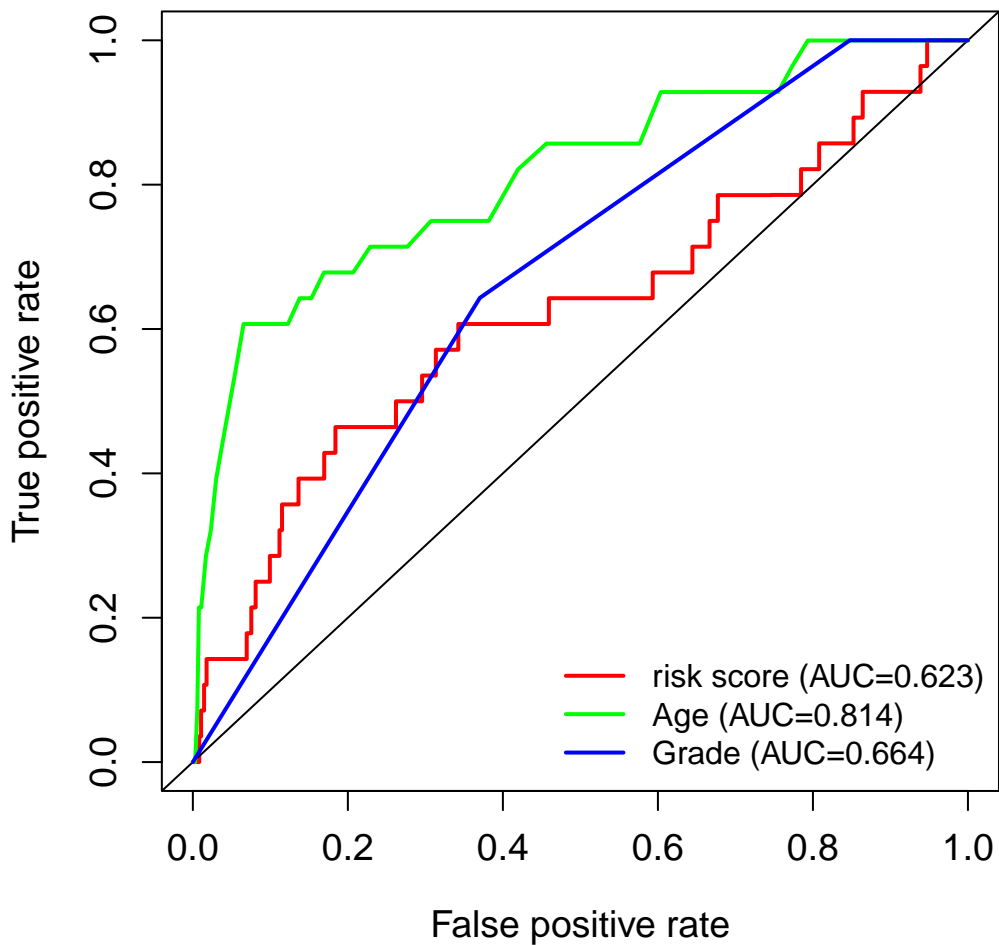

Supplement: Supplementary file 3 [file DataSheet_3.zip › 6.cli_roc/GEO_cliROC.pdf]

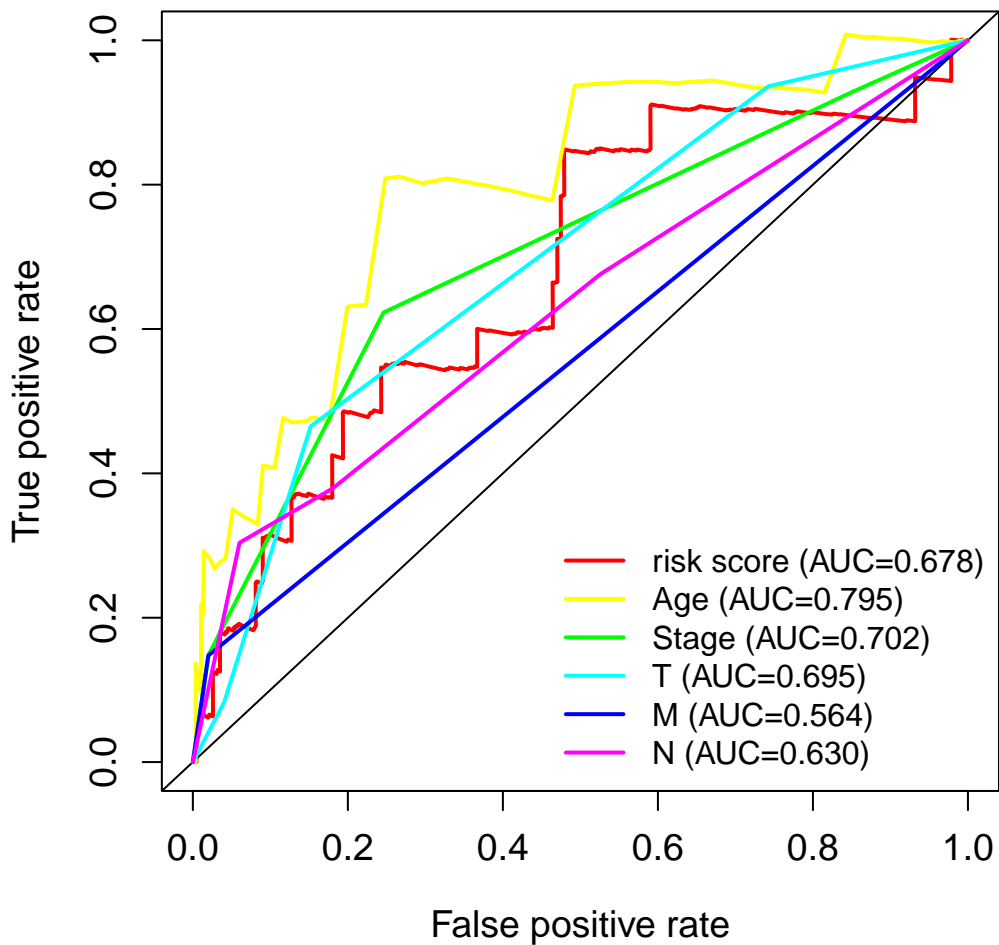

Supplement: Supplementary file 3 [file DataSheet_3.zip › 6.cli_roc/tcga.traincliROC.pdf]

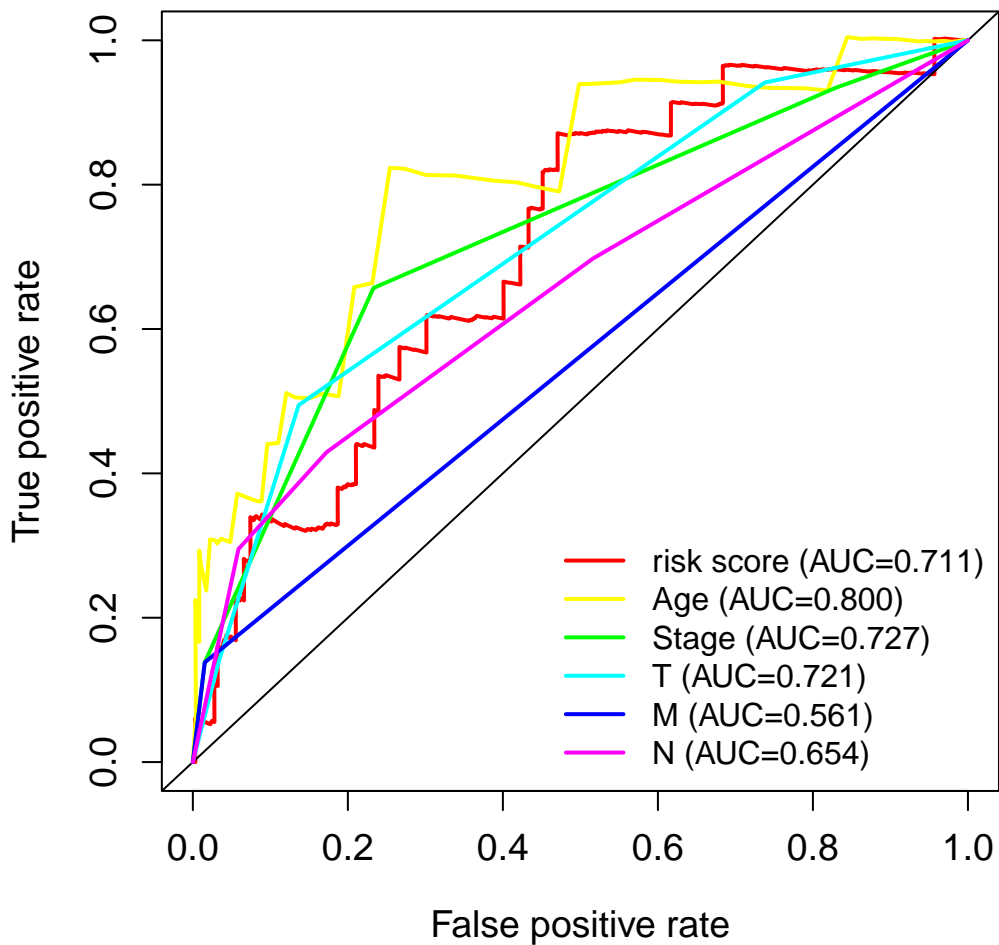

Supplement: Supplementary file 3 [file DataSheet_3.zip › 6.cli_roc/TCGA_ALL_cliROC.pdf]

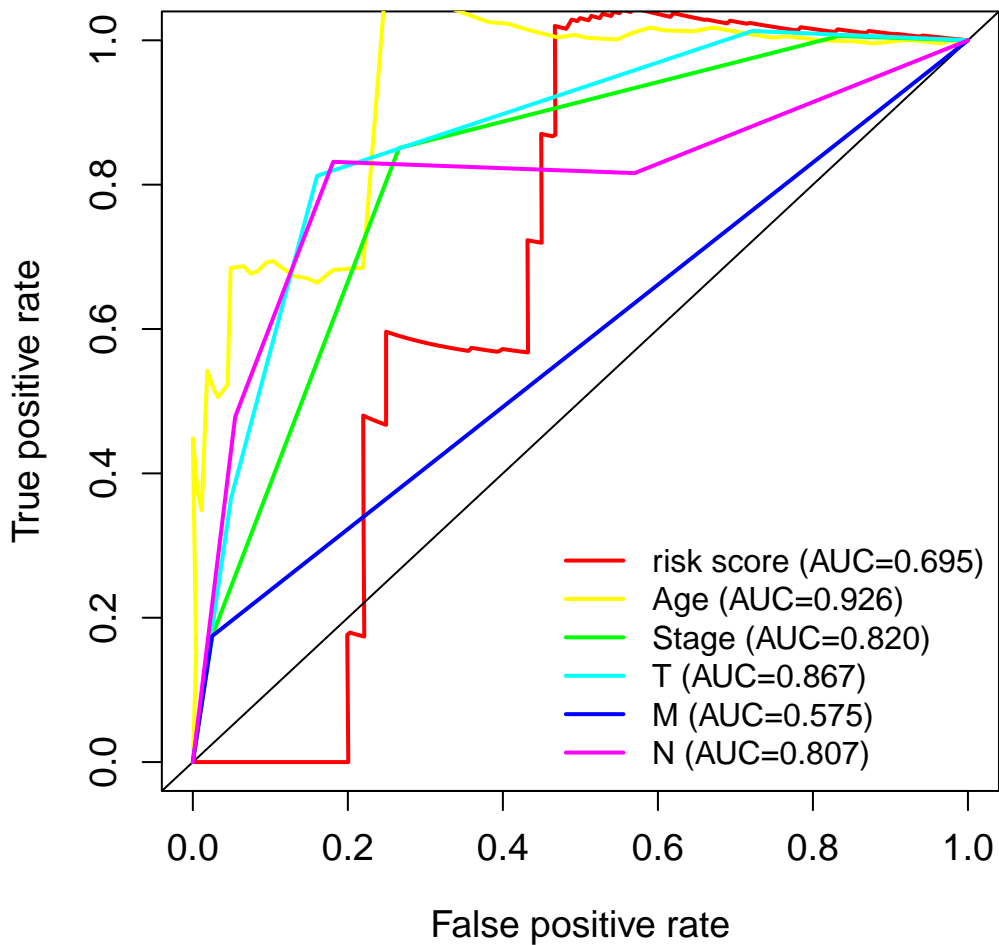

Supplement: Supplementary file 3 [file DataSheet_3.zip › 6.cli_roc/tcga_test_cliROC.pdf]

# Patients with >65

Risk + high + low

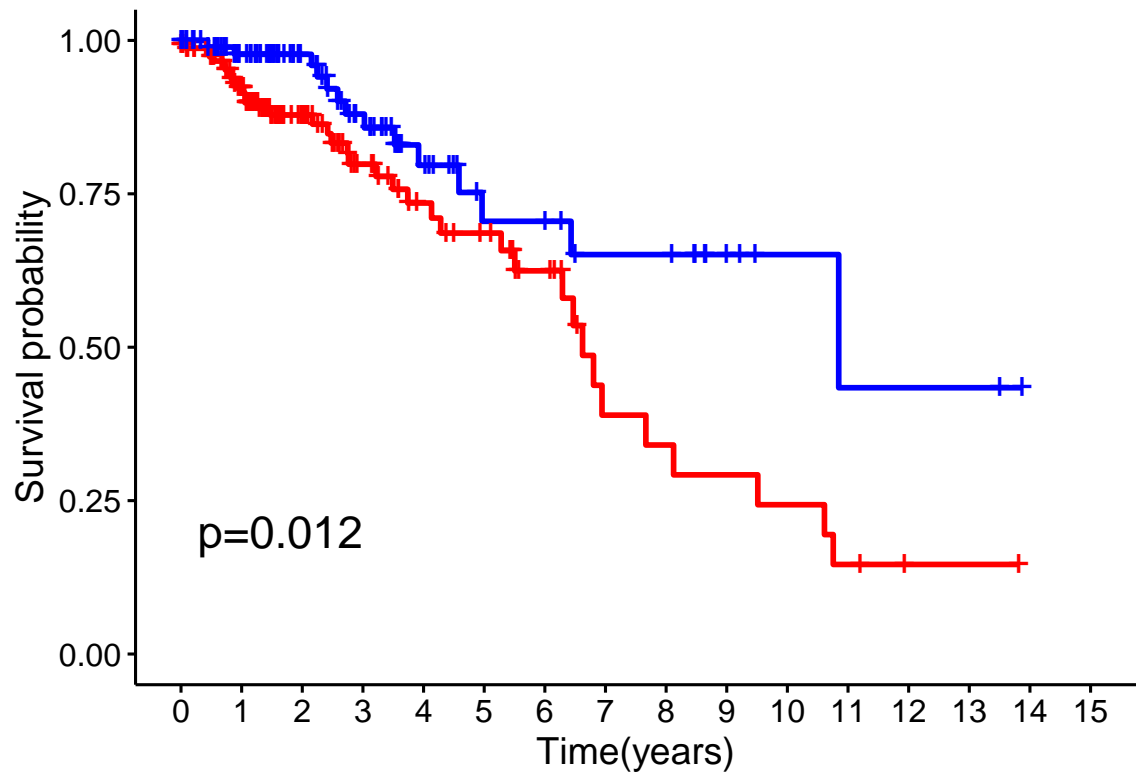

Supplement: Supplementary file 3 [file DataSheet_3.zip › 7.cliCor/survival.Age_gt65.pdf]

# Patients with $\leq 65$

Risk + high + low

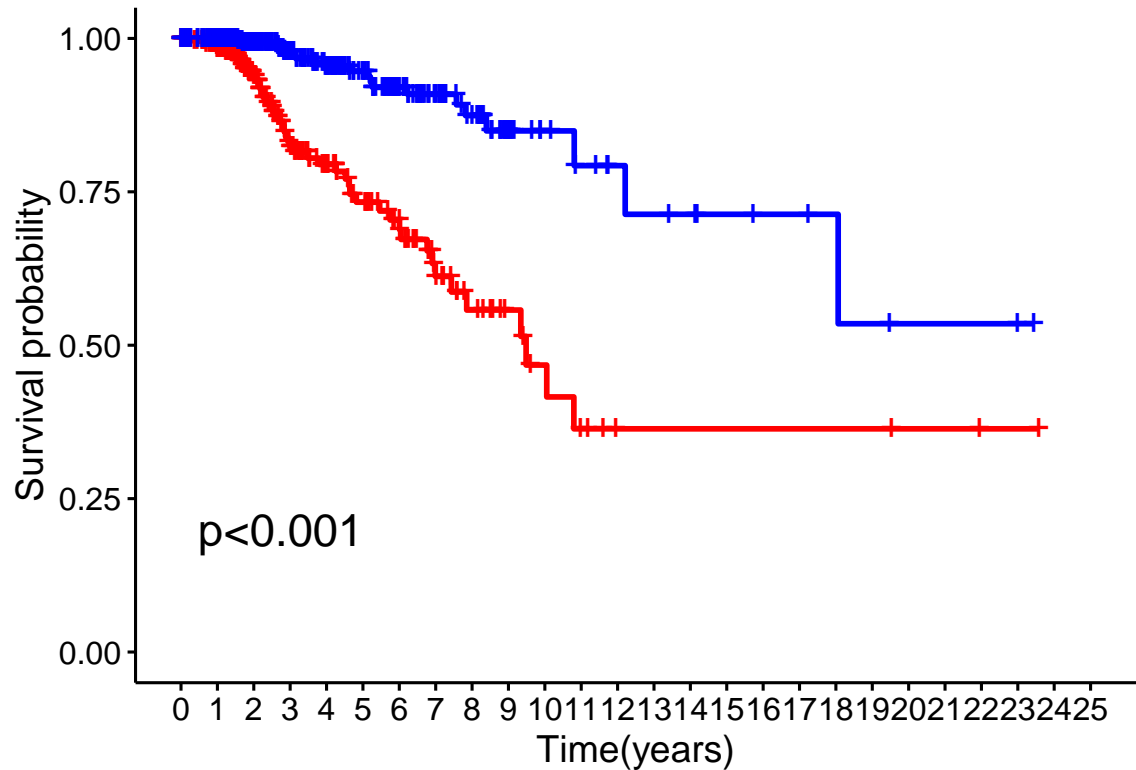

Supplement: Supplementary file 3 [file DataSheet_3.zip › 7.cliCor/survival.Age_le65.pdf]

# Patients with FEMALE

Risk + high + low

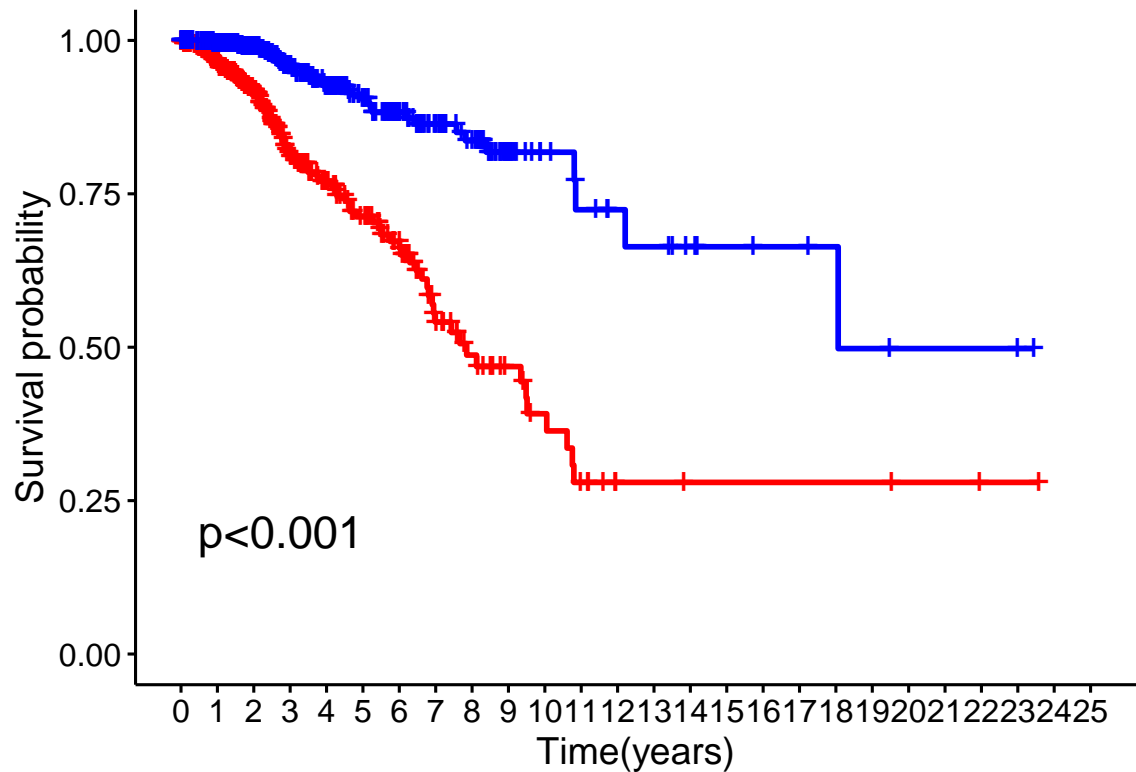

Supplement: Supplementary file 3 [file DataSheet_3.zip › 7.cliCor/survival.Gender_FEMALE.pdf]

# Patients with MALE

Risk + high + low

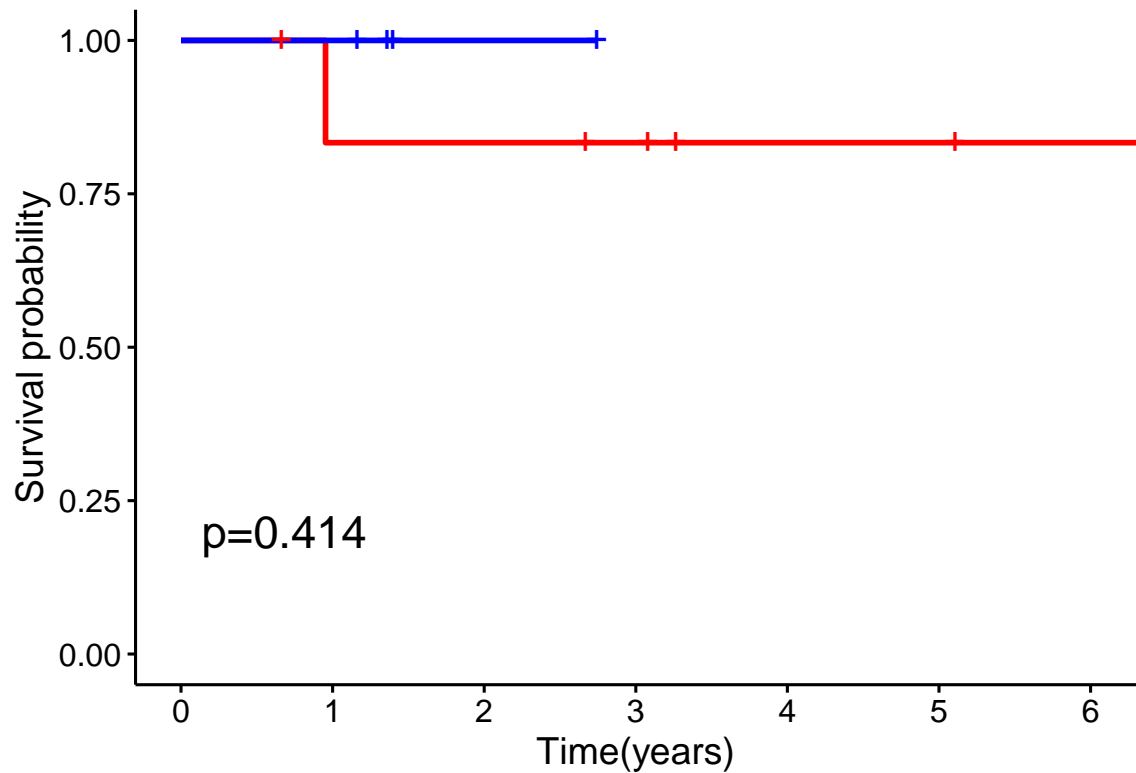

Supplement: Supplementary file 3 [file DataSheet_3.zip › 7.cliCor/survival.Gender_MALE.pdf]

# Patients with N0-1

Risk + high + low

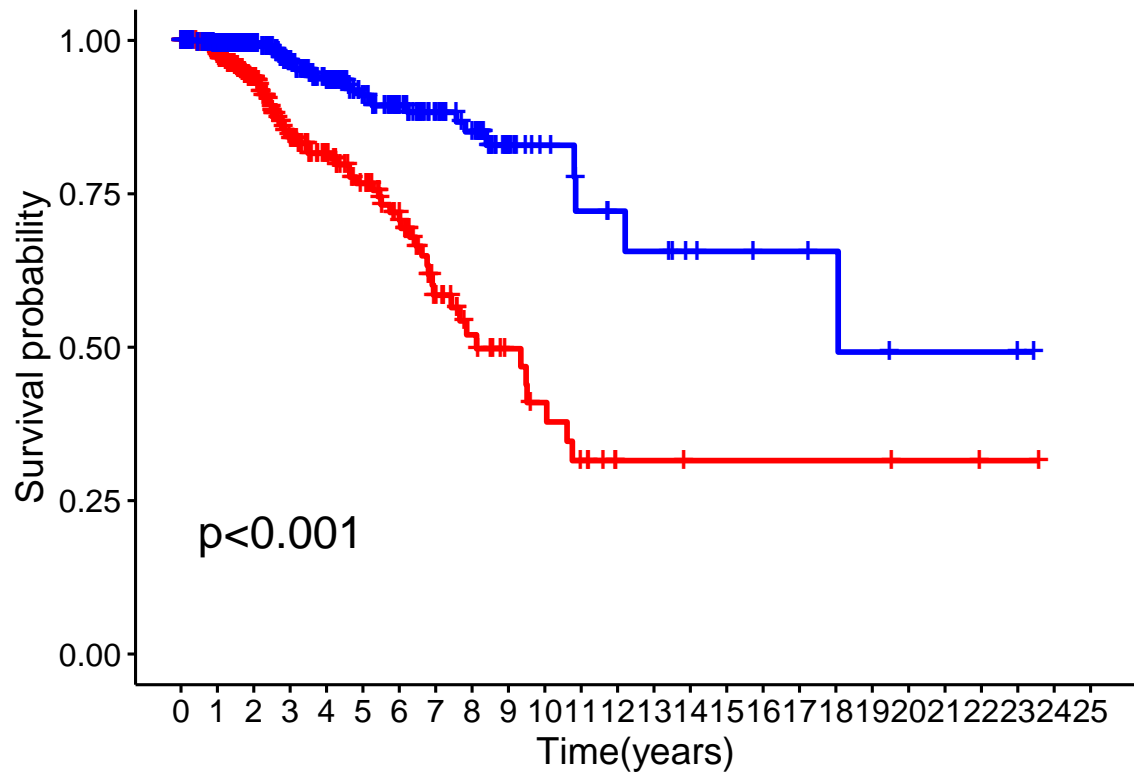

Supplement: Supplementary file 3 [file DataSheet_3.zip › 7.cliCor/survival.N_N0-1.pdf]

# Patients with N2-3

Risk + high + low

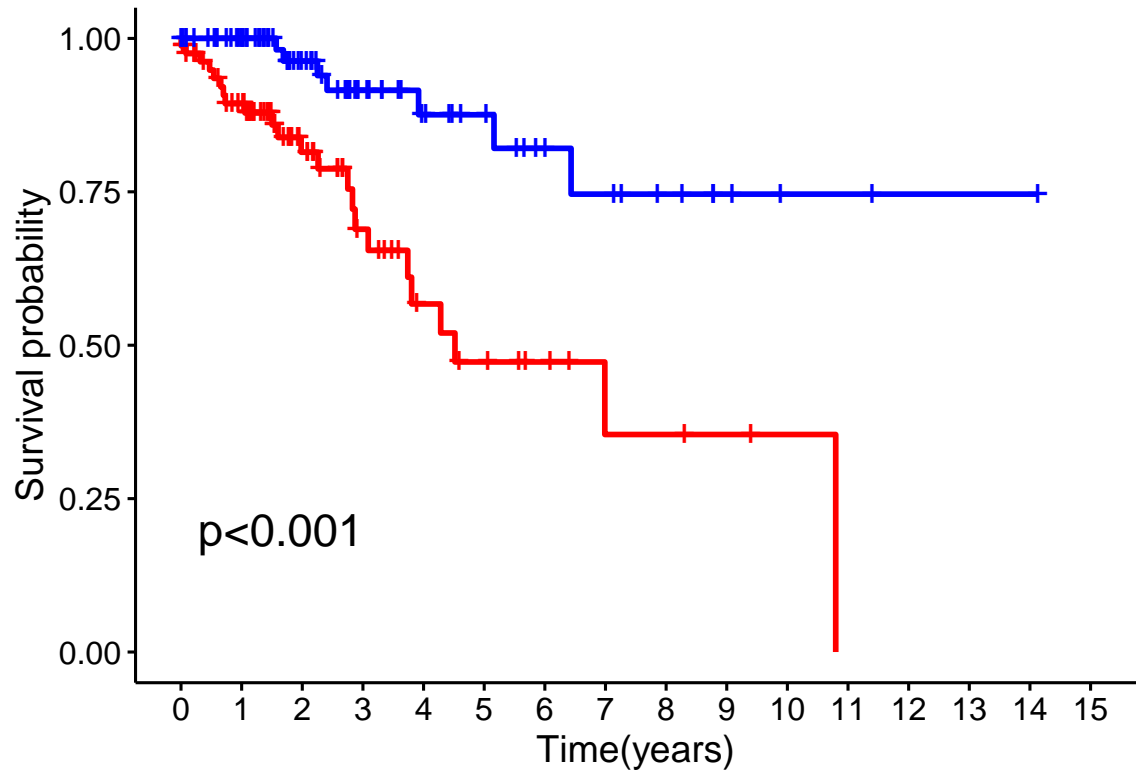

Supplement: Supplementary file 3 [file DataSheet_3.zip › 7.cliCor/survival.N_N2-3.pdf]

# Patients with Stage I-II

Risk + high + low

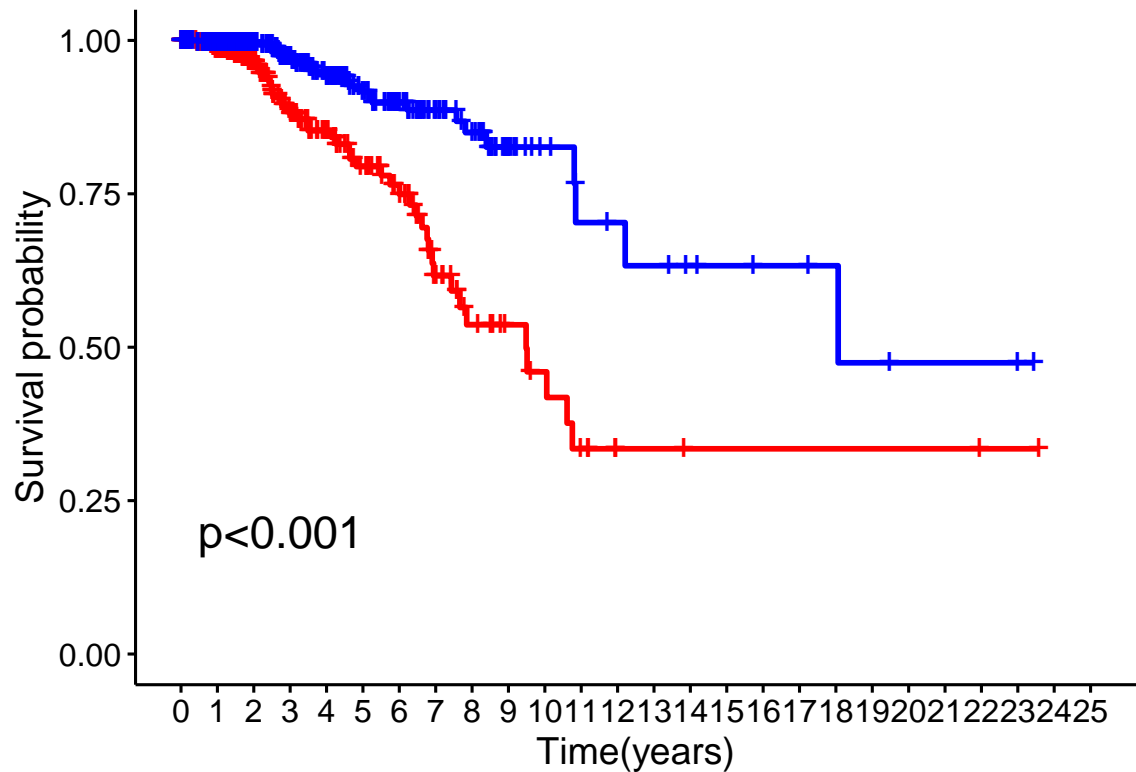

Supplement: Supplementary file 3 [file DataSheet_3.zip › 7.cliCor/survival.Stage_Stage I-II.pdf]

# Patients with Stage III–IV

Risk    + high    + low

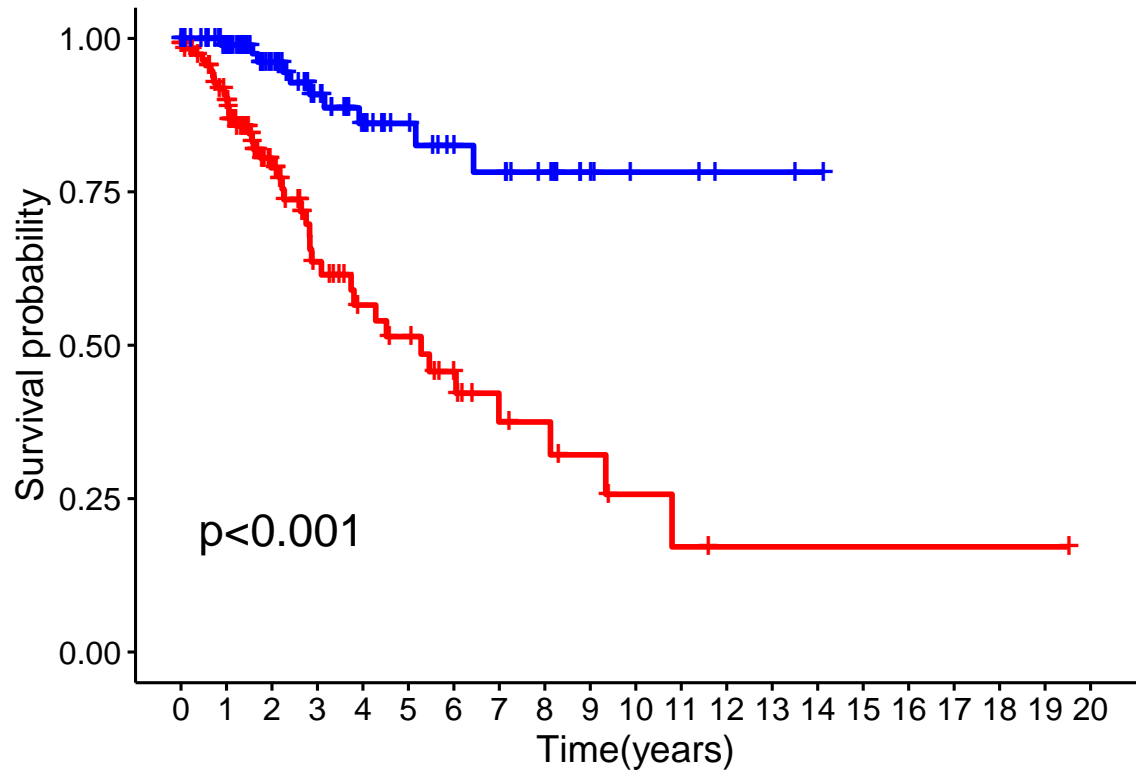

Supplement: Supplementary file 3 [file DataSheet_3.zip › 7.cliCor/survival.Stage_Stage III-IV.pdf]

# Patients with T1-2

Risk + high + low

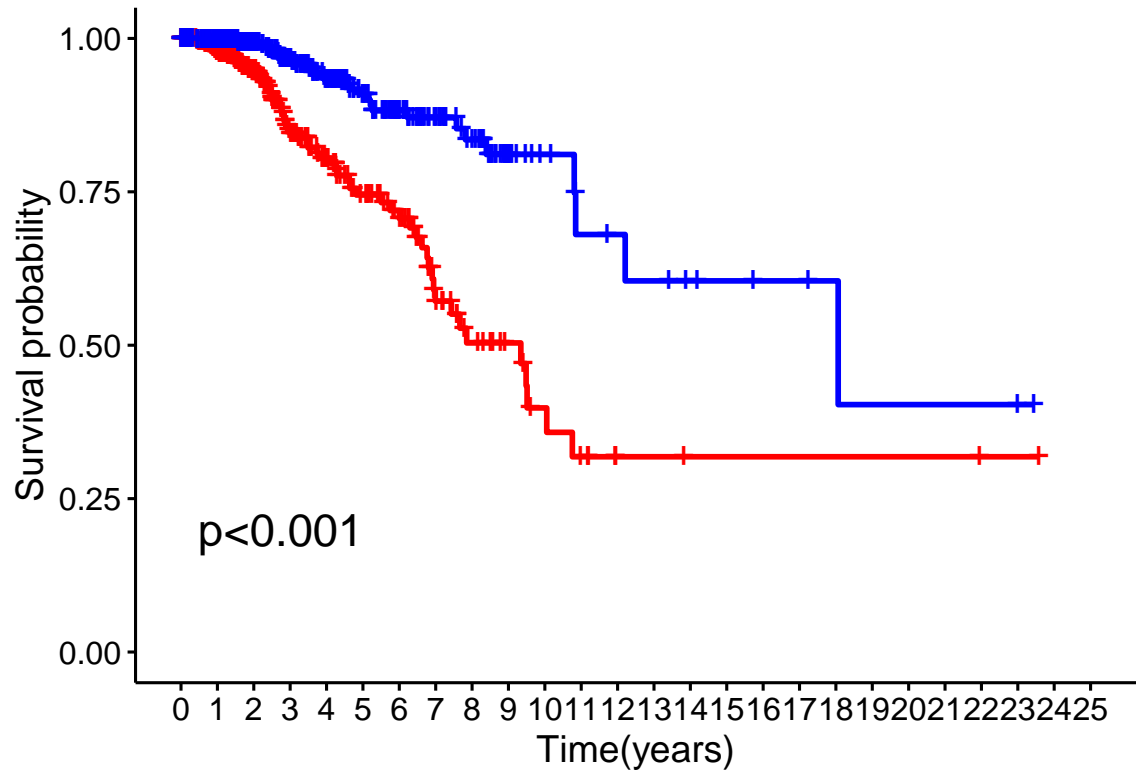

Supplement: Supplementary file 3 [file DataSheet_3.zip › 7.cliCor/survival.T_T1-2.pdf]

# Patients with T3-4

Risk + high + low

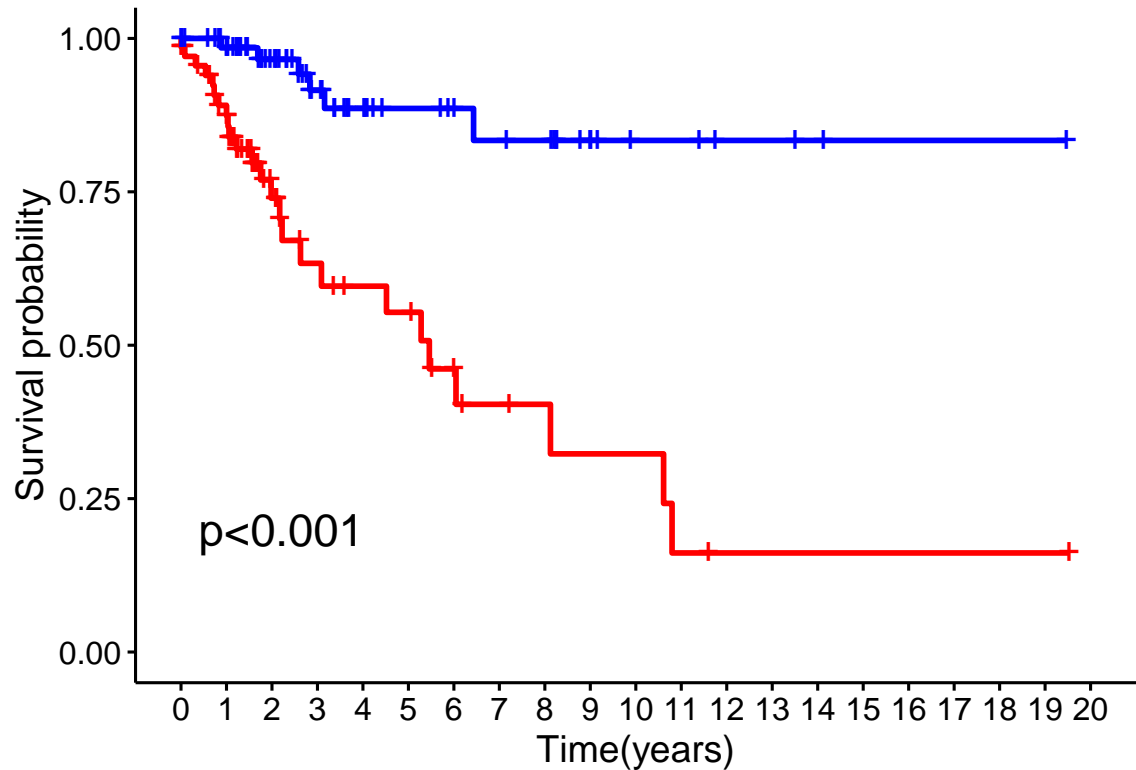

Supplement: Supplementary file 3 [file DataSheet_3.zip › 7.cliCor/survival.T_T3-4.pdf]

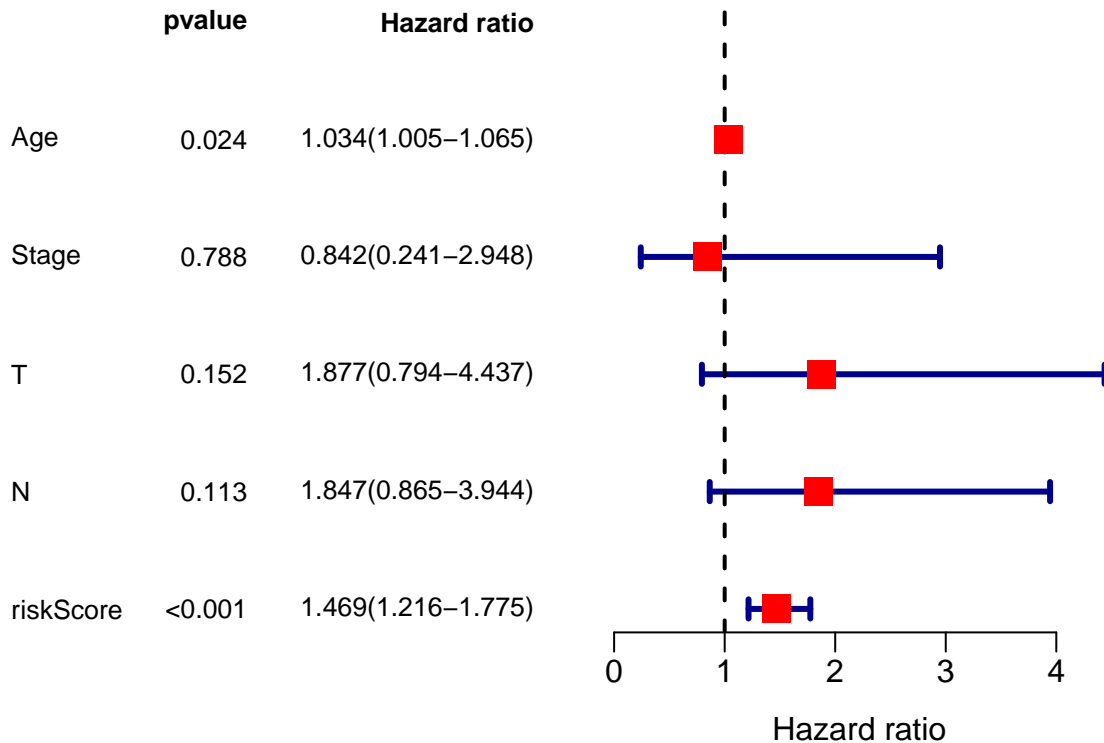

Supplement: Supplementary file 3 [file DataSheet_3.zip › 8.Nomo/35.Forest/TCGAtestmultiForest.pdf]

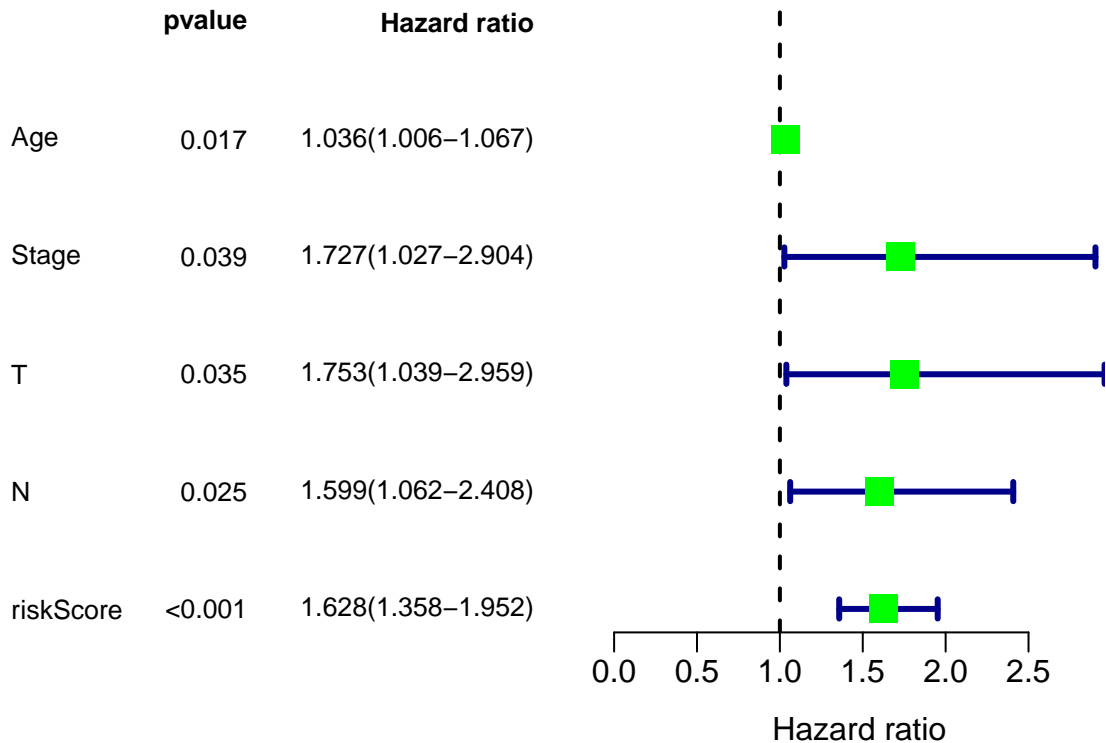

Supplement: Supplementary file 3 [file DataSheet_3.zip › 8.Nomo/35.Forest/TCGAtestuniForest.pdf]

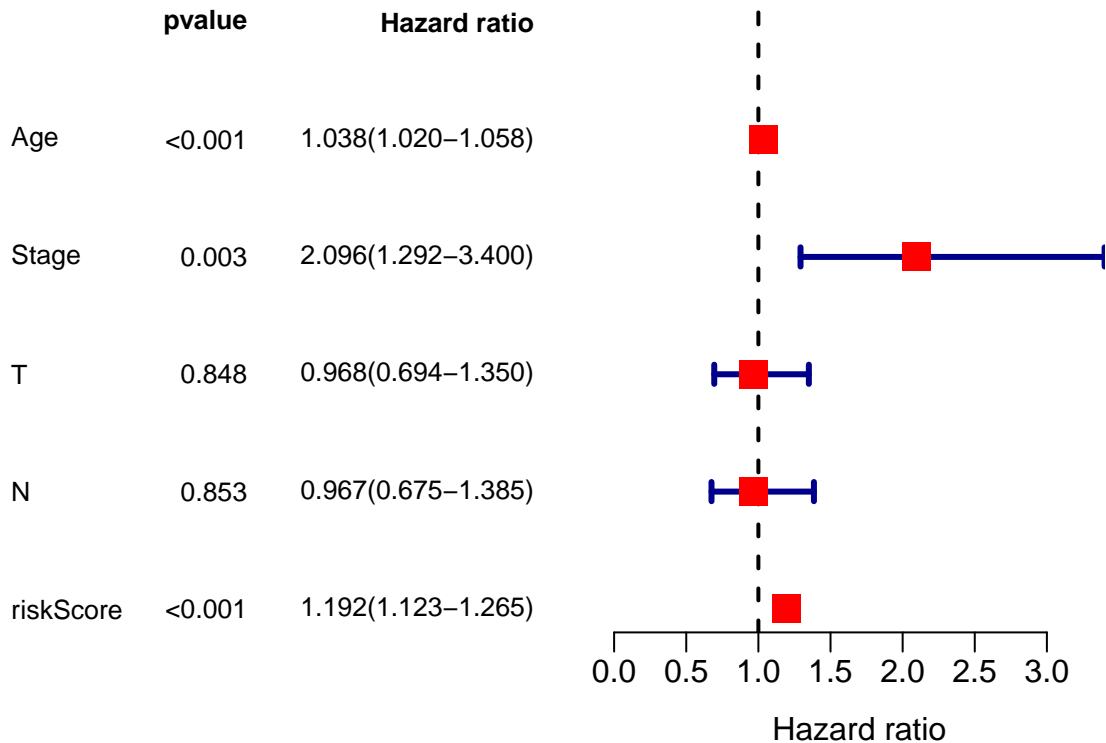

Supplement: Supplementary file 3 [file DataSheet_3.zip › 8.Nomo/35.Forest/trainmultiForest.pdf]

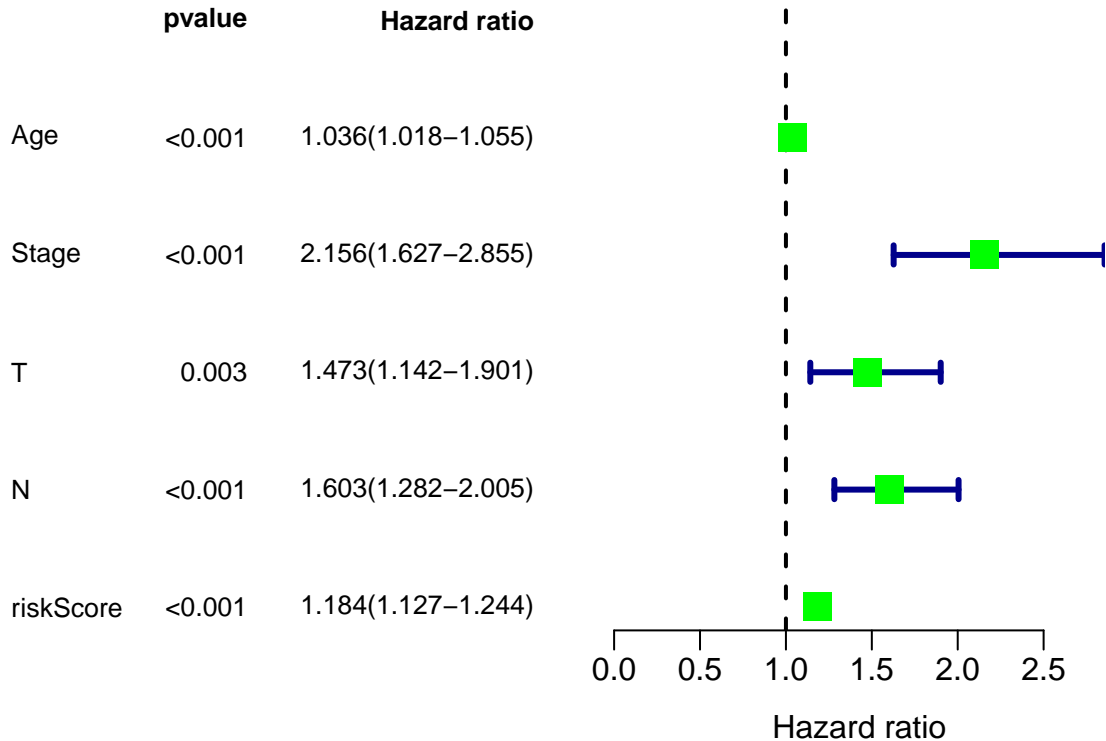

Supplement: Supplementary file 3 [file DataSheet_3.zip › 8.Nomo/35.Forest/trainuniForest.pdf]

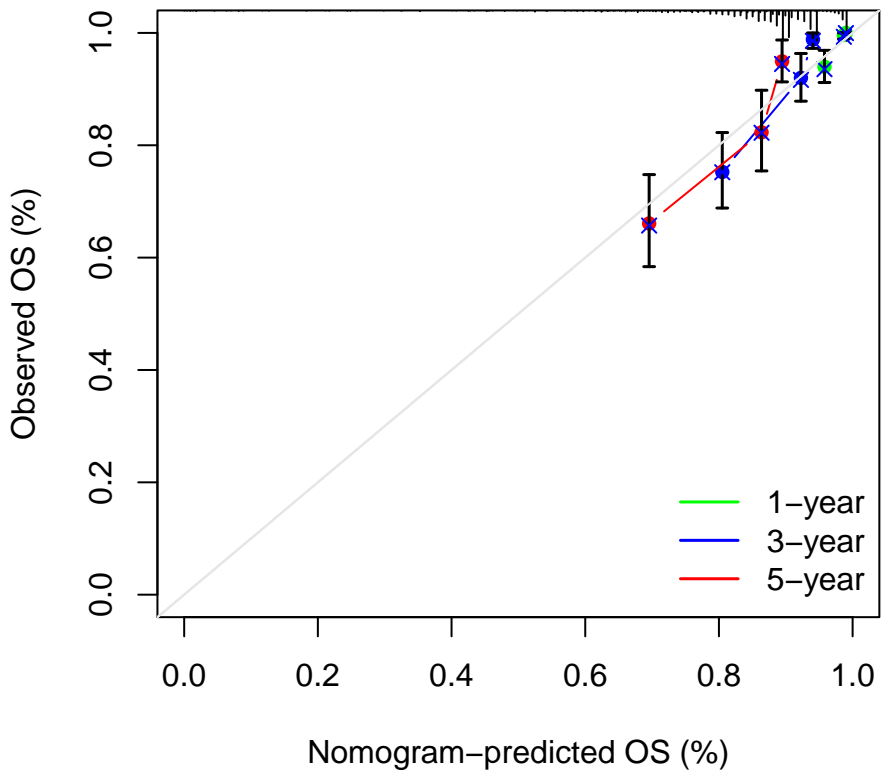

Supplement: Supplementary file 3 [file DataSheet_3.zip › 8.Nomo/calibration.pdf]

Strata + nomoRisk=High + nomoRisk=Low

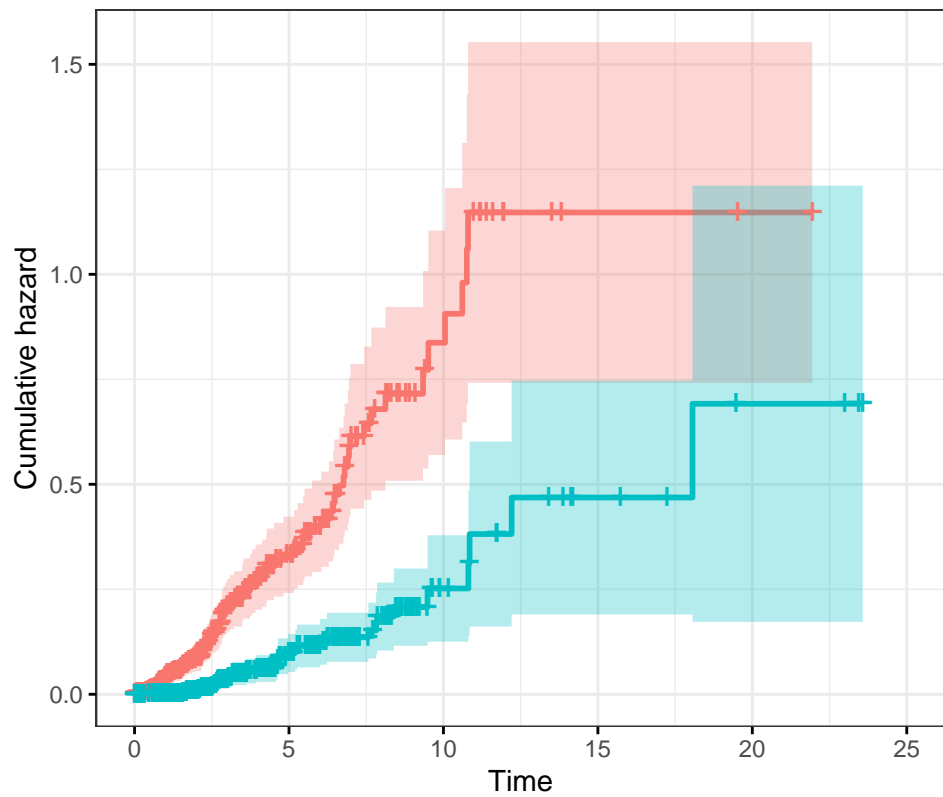

Supplement: Supplementary file 3 [file DataSheet_3.zip › 8.Nomo/cumulative.pdf]

Points

N

T

Gender

M

Stage\*

Age\*\*\*

risk\*\*\*

**Total points**

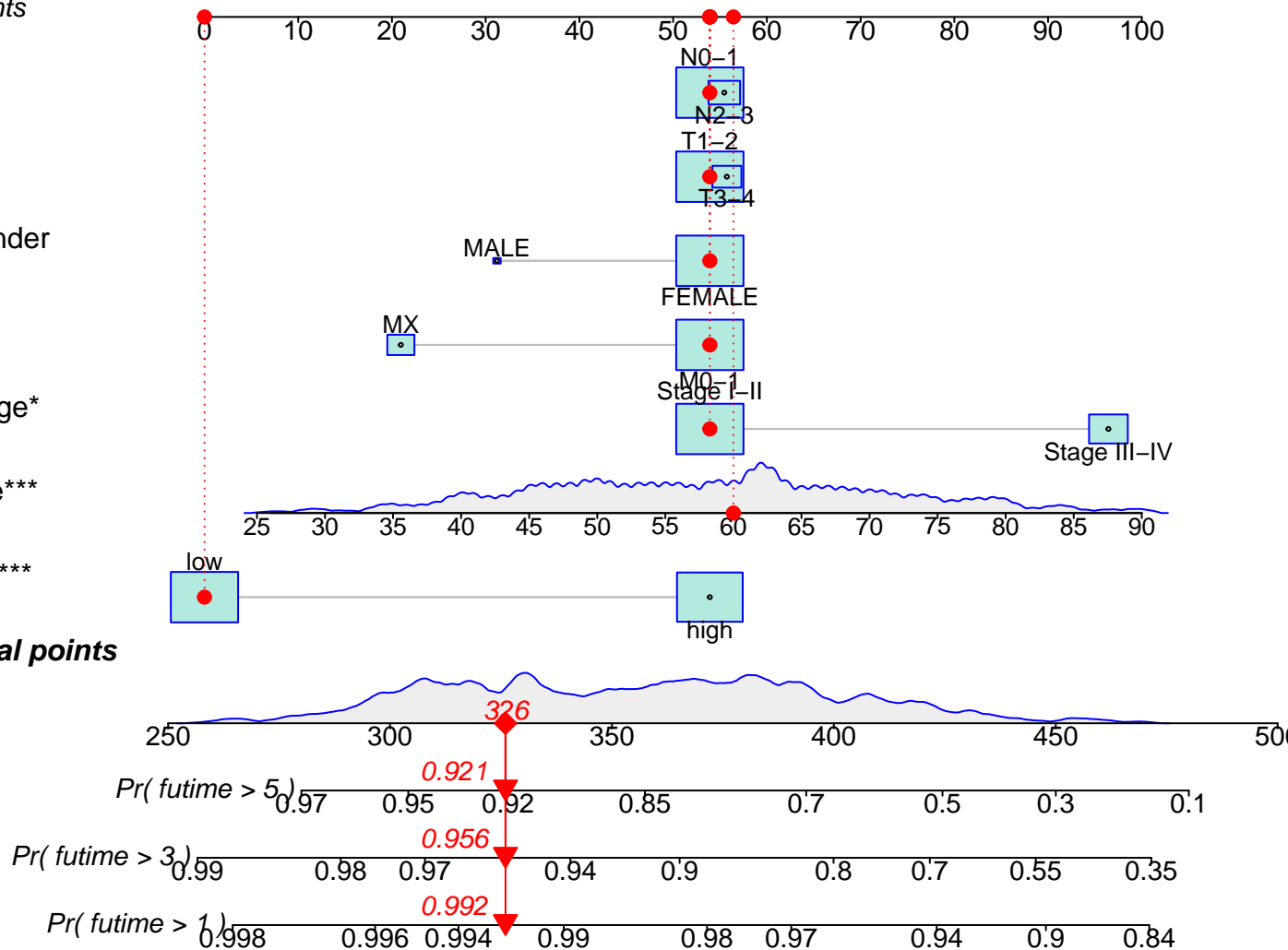

Supplement: Supplementary file 3 [file DataSheet_3.zip › 8.Nomo/Nomo.pdf]

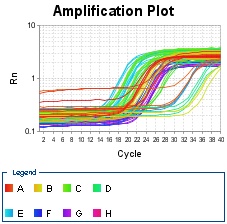

Supplement: Supplementary file 4 [file DataSheet_4.zip › 14.PCR-2023-2-12/PCR-2023-2-12/1/1-Amplification Plot.jpg]

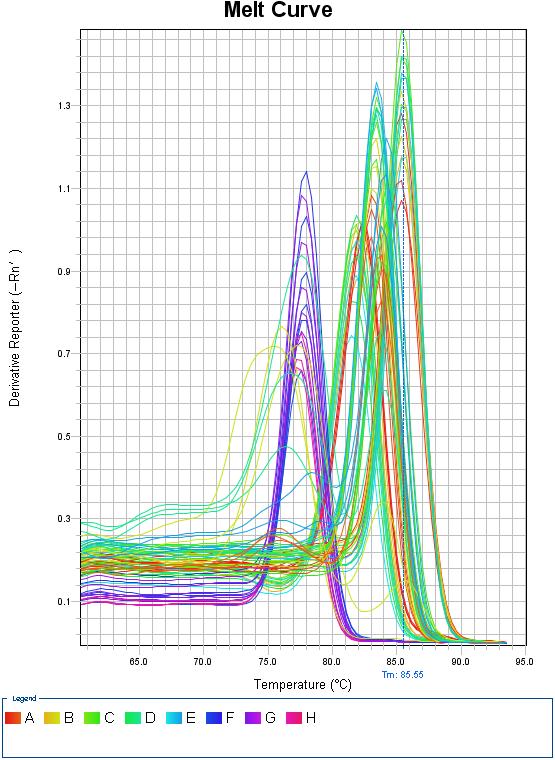

Supplement: Supplementary file 4 [file DataSheet_4.zip › 14.PCR-2023-2-12/PCR-2023-2-12/1/1-Melt Curve.jpg]

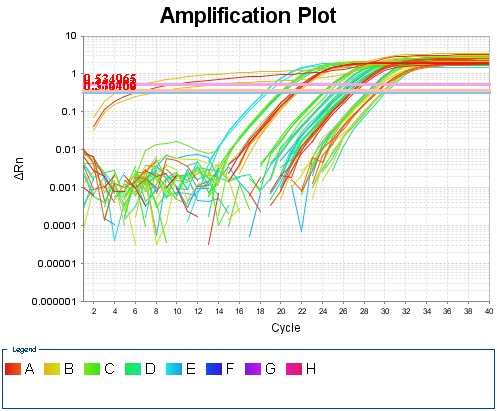

Supplement: Supplementary file 4 [file DataSheet_4.zip › 14.PCR-2023-2-12/PCR-2023-2-12/2/Amplification Plot.jpg]

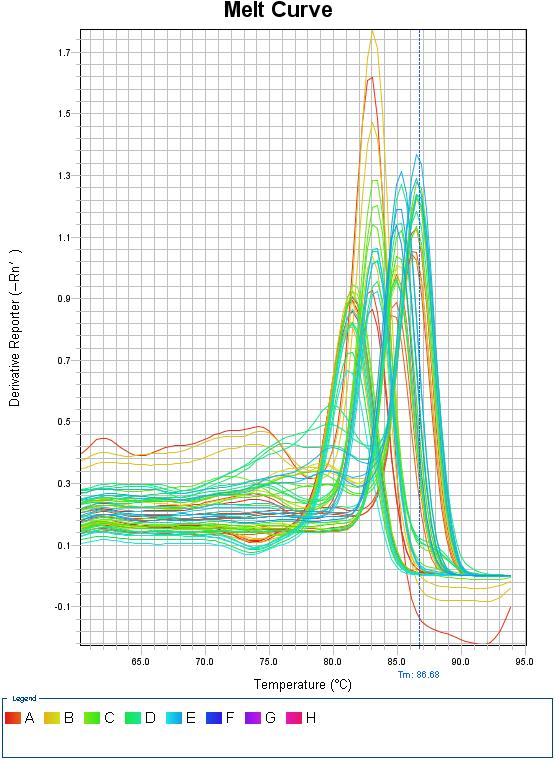

Supplement: Supplementary file 4 [file DataSheet_4.zip › 14.PCR-2023-2-12/PCR-2023-2-12/2/Melt Curve.jpg]

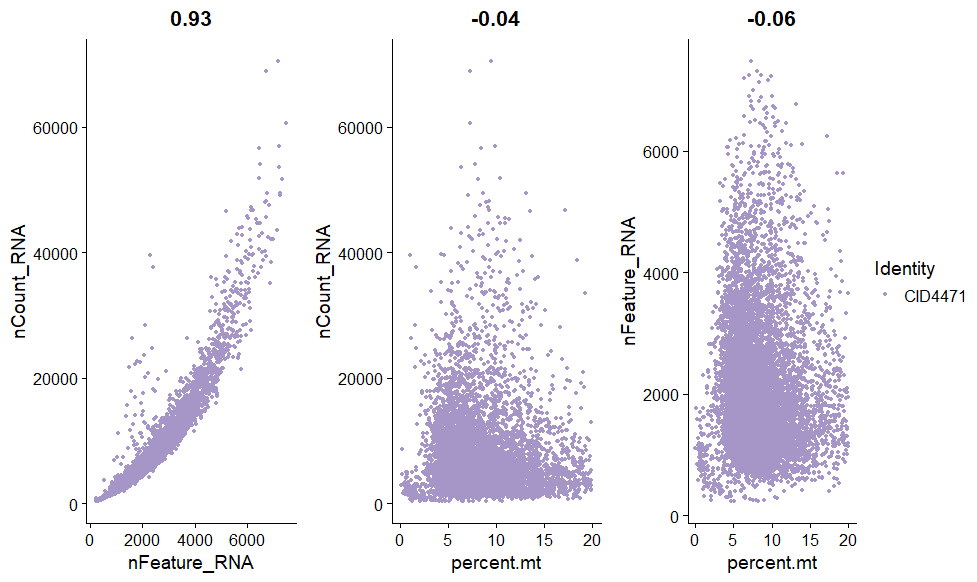

Supplement: Supplementary file 4 [file DataSheet_4.zip › 13.GSM5354529_CID4471/Rplot.png]

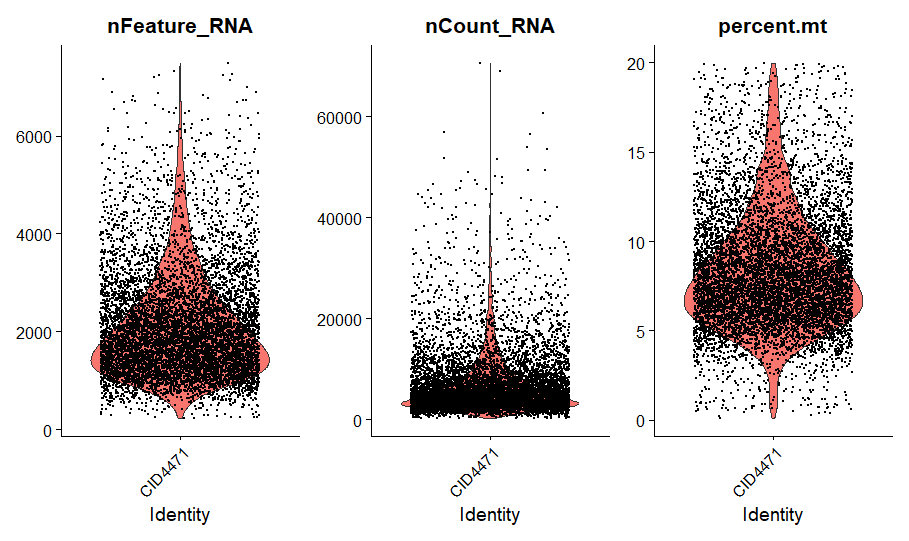

Supplement: Supplementary file 4 [file DataSheet_4.zip › 13.GSM5354529_CID4471/Rplot01.png]

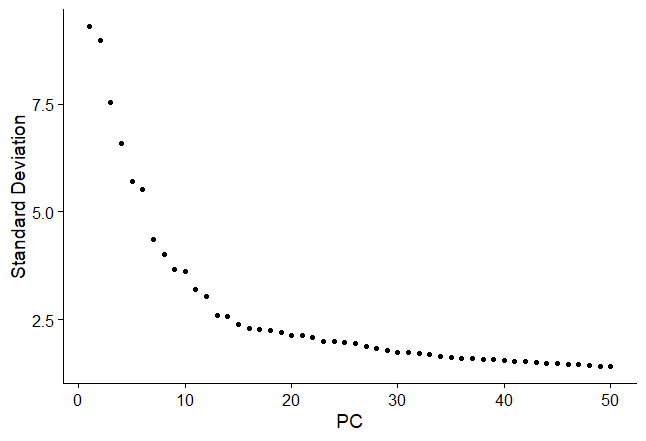

Supplement: Supplementary file 4 [file DataSheet_4.zip › 13.GSM5354529_CID4471/Rplot02.png]

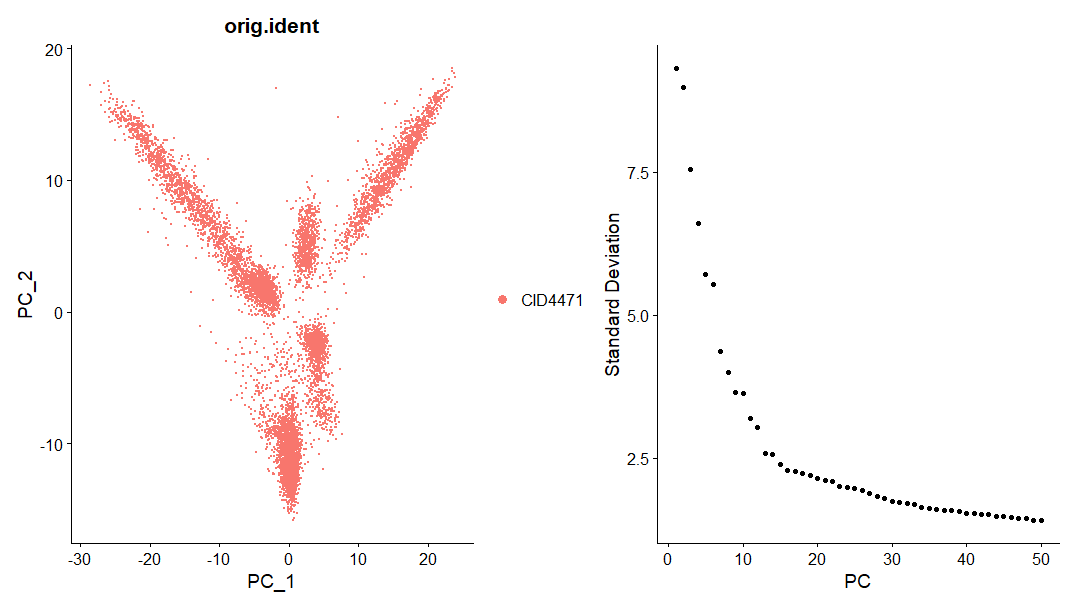

Supplement: Supplementary file 4 [file DataSheet_4.zip › 13.GSM5354529_CID4471/Rplot03.png]

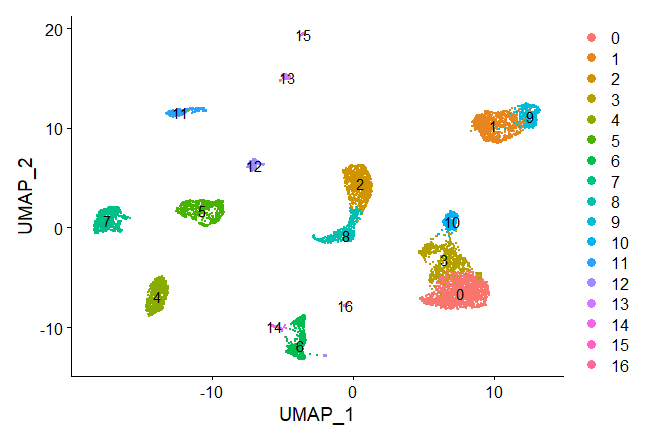

Supplement: Supplementary file 4 [file DataSheet_4.zip › 13.GSM5354529_CID4471/Rplot04.png]

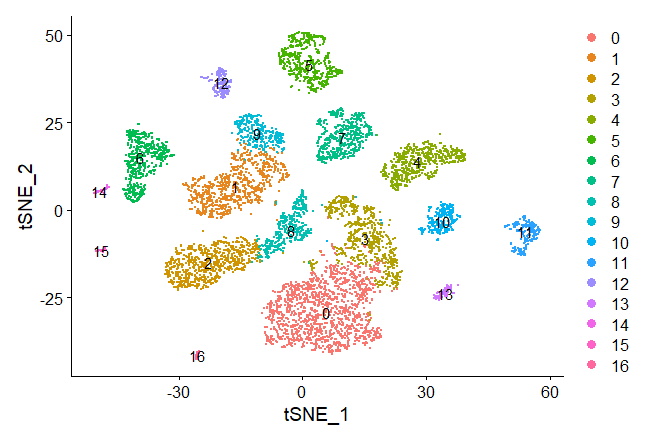

Supplement: Supplementary file 4 [file DataSheet_4.zip › 13.GSM5354529_CID4471/Rplot05.png]

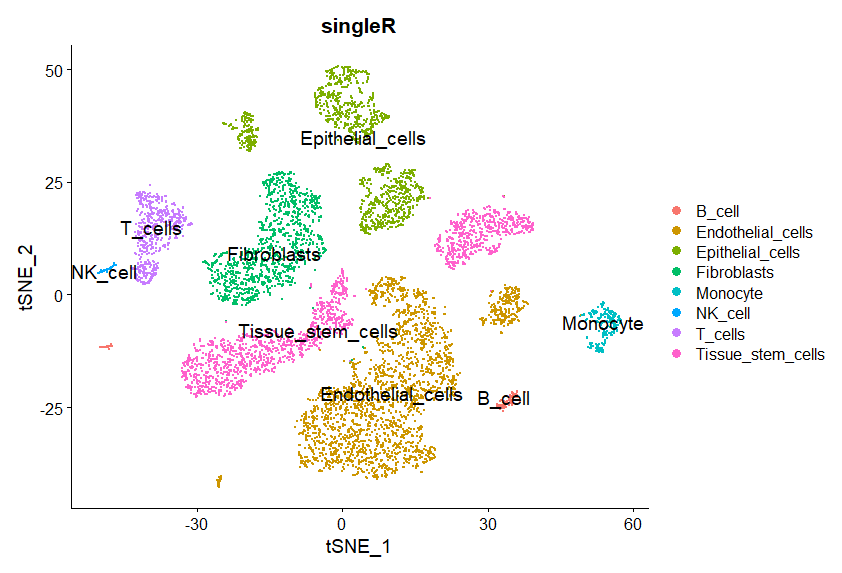

Supplement: Supplementary file 4 [file DataSheet_4.zip › 13.GSM5354529_CID4471/Rplot06.png]

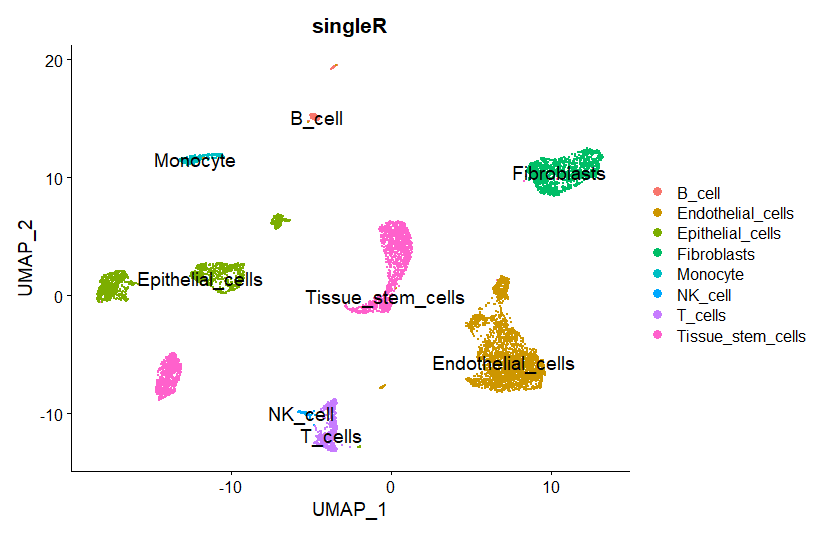

Supplement: Supplementary file 4 [file DataSheet_4.zip › 13.GSM5354529_CID4471/Rplot07.png]

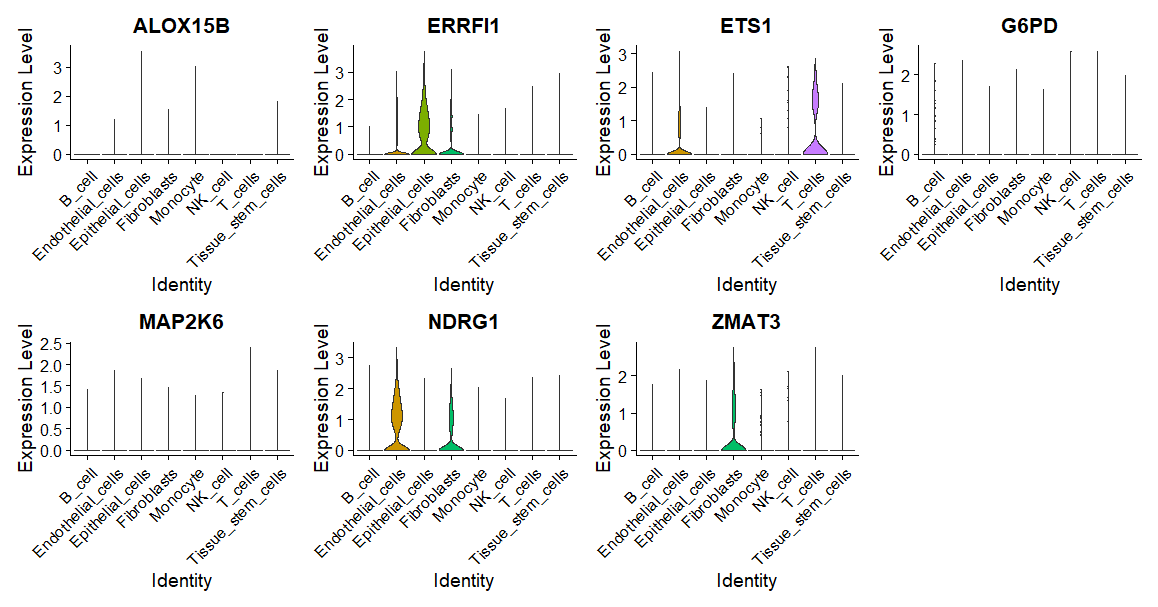

Supplement: Supplementary file 4 [file DataSheet_4.zip › 13.GSM5354529_CID4471/Rplot08.png]

Risk 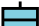 low 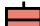 high

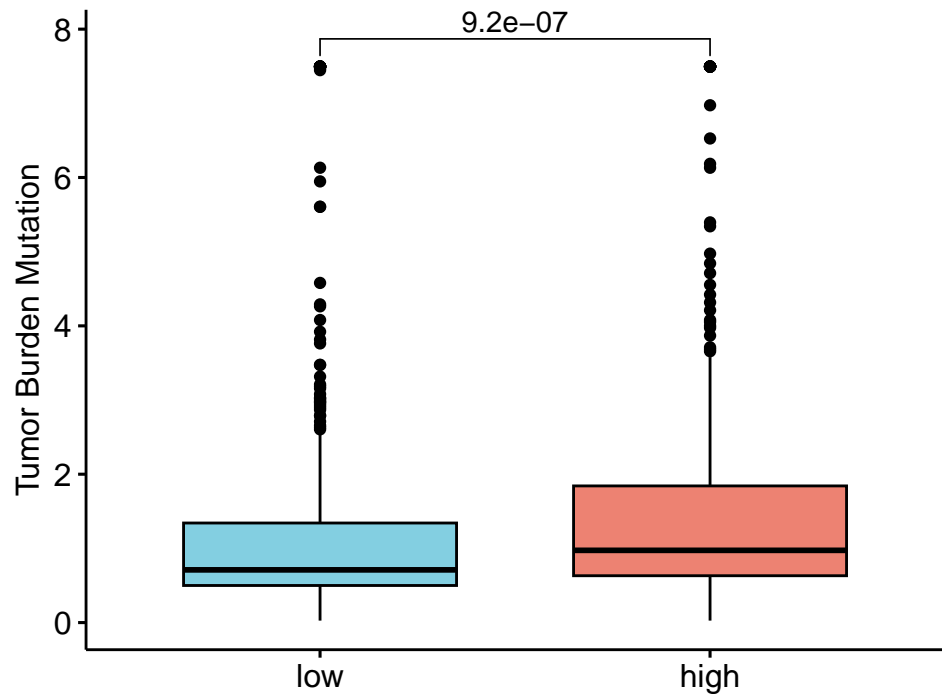

Supplement: Supplementary file 5 [file DataSheet_5.zip › 11.TUbian/boxplot.pdf]

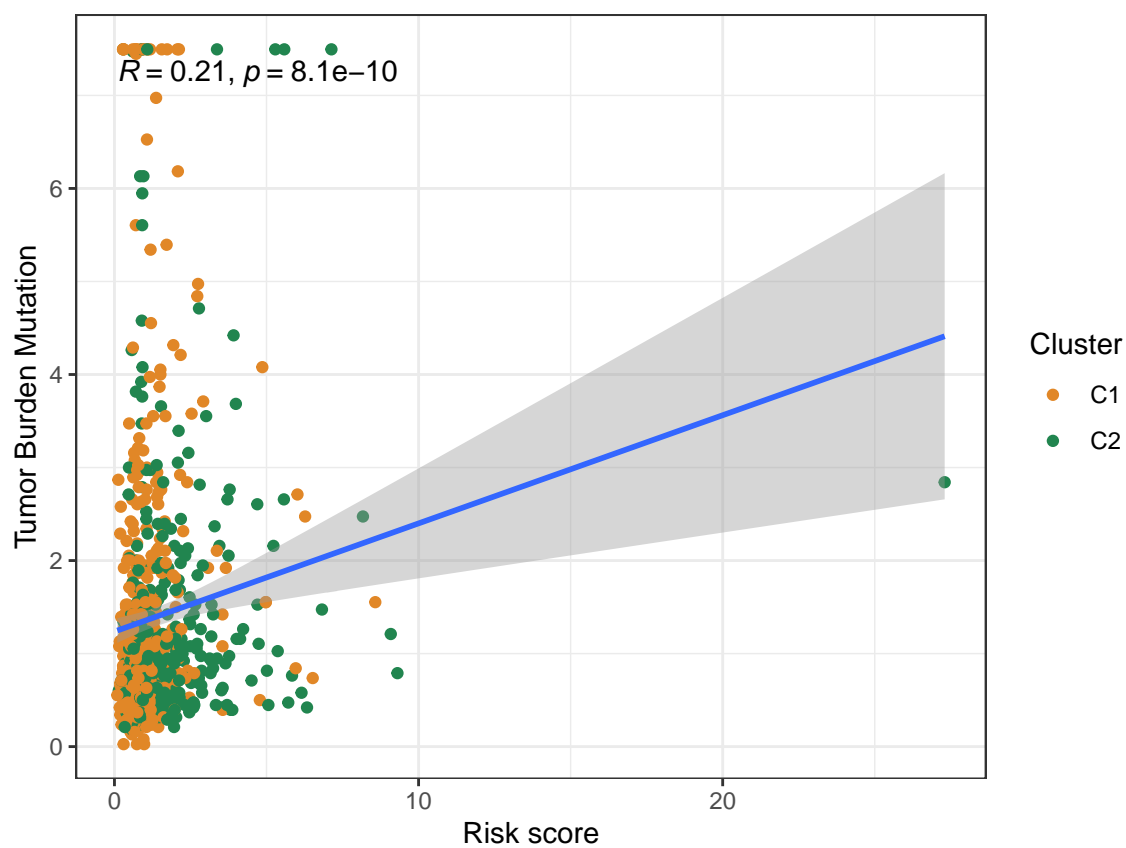

Supplement: Supplementary file 5 [file DataSheet_5.zip › 11.TUbian/cor.pdf]

# Altered in 364 (85.85%) of 424 samples.

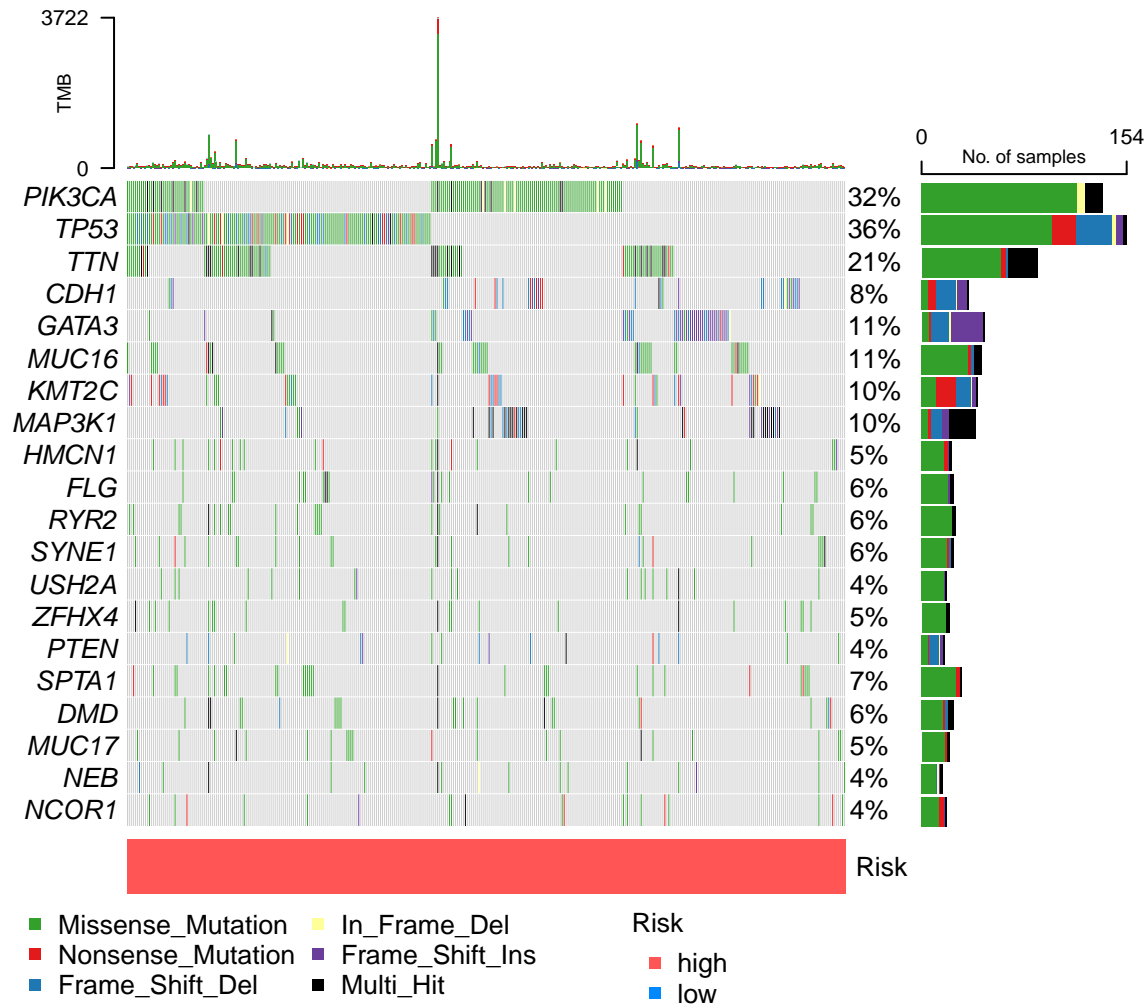

Supplement: Supplementary file 5 [file DataSheet_5.zip › 11.TUbian/high.pdf]

# Altered in 348 (85.29%) of 408 samples.

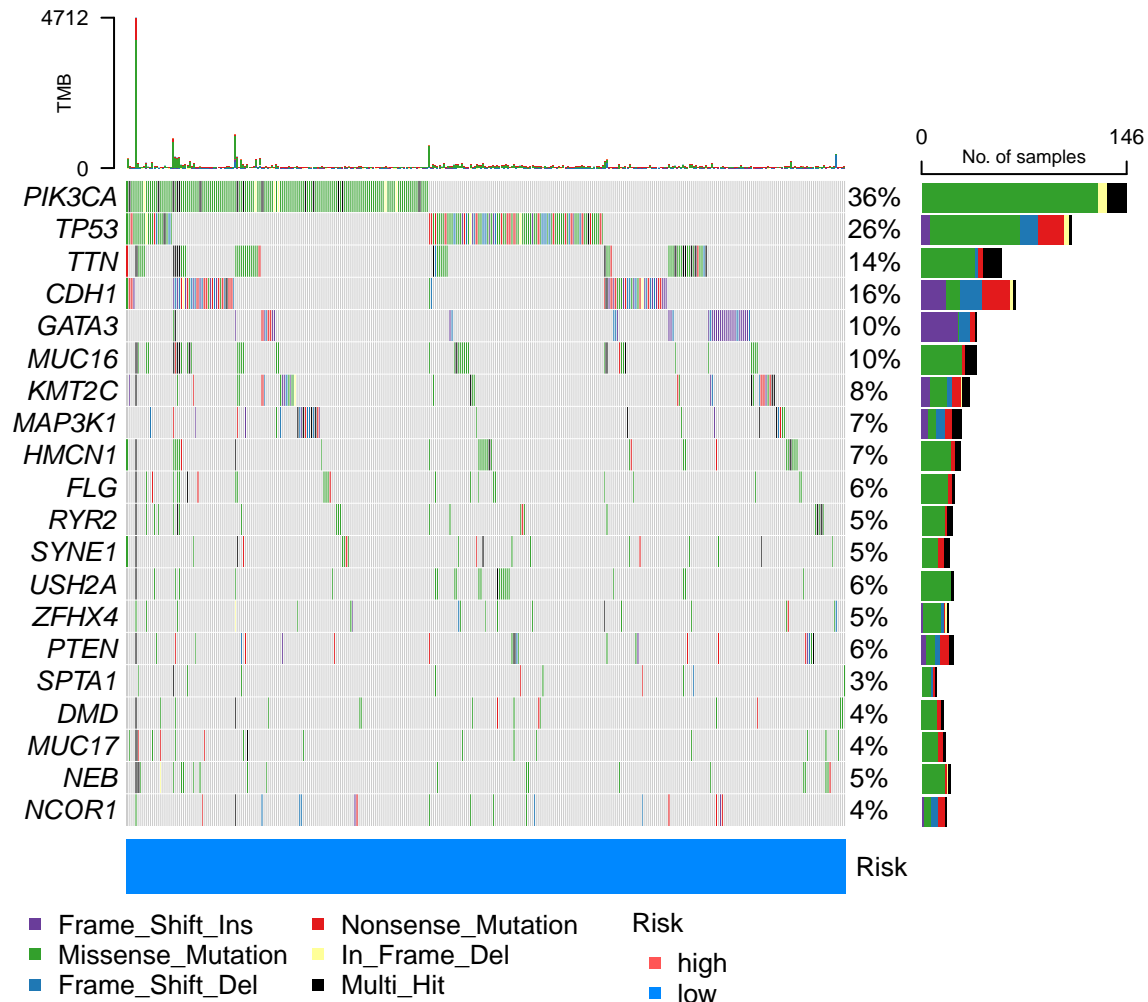

Supplement: Supplementary file 5 [file DataSheet_5.zip › 11.TUbian/low.pdf]

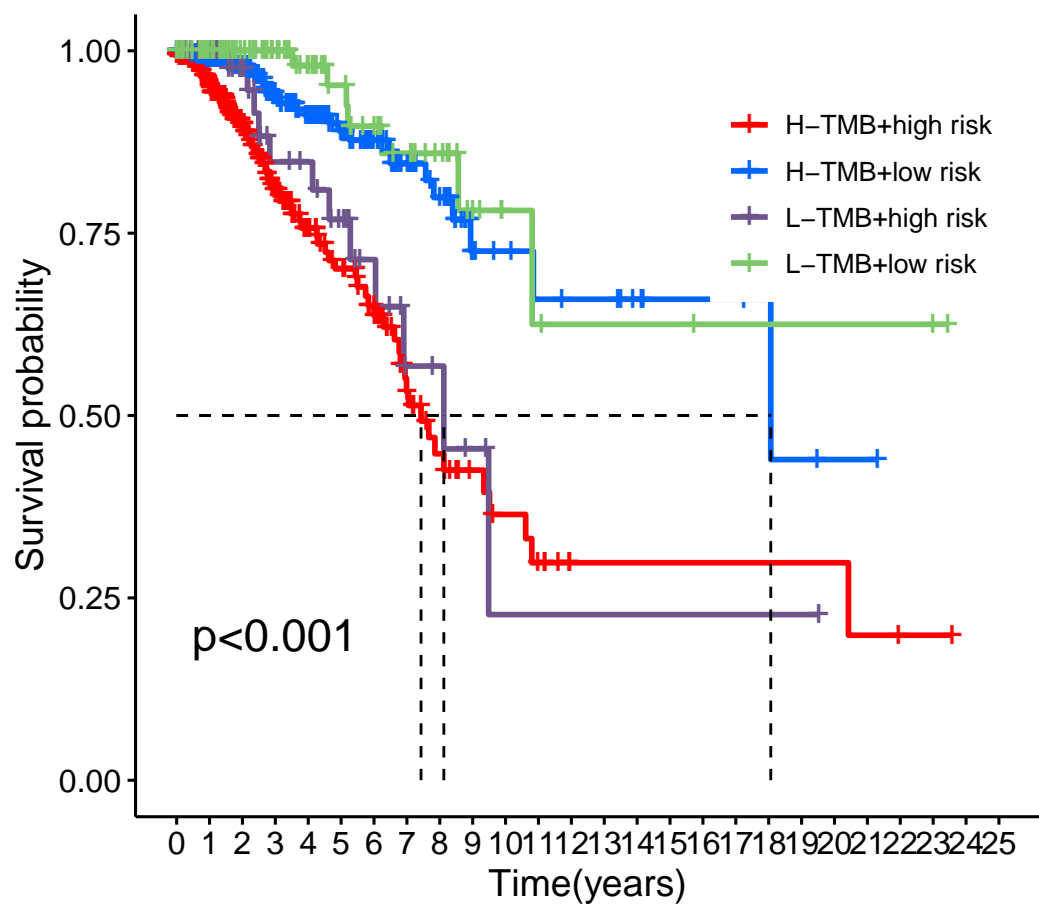

Supplement: Supplementary file 5 [file DataSheet_5.zip › 11.TUbian/TMB-risk.survival.pdf]

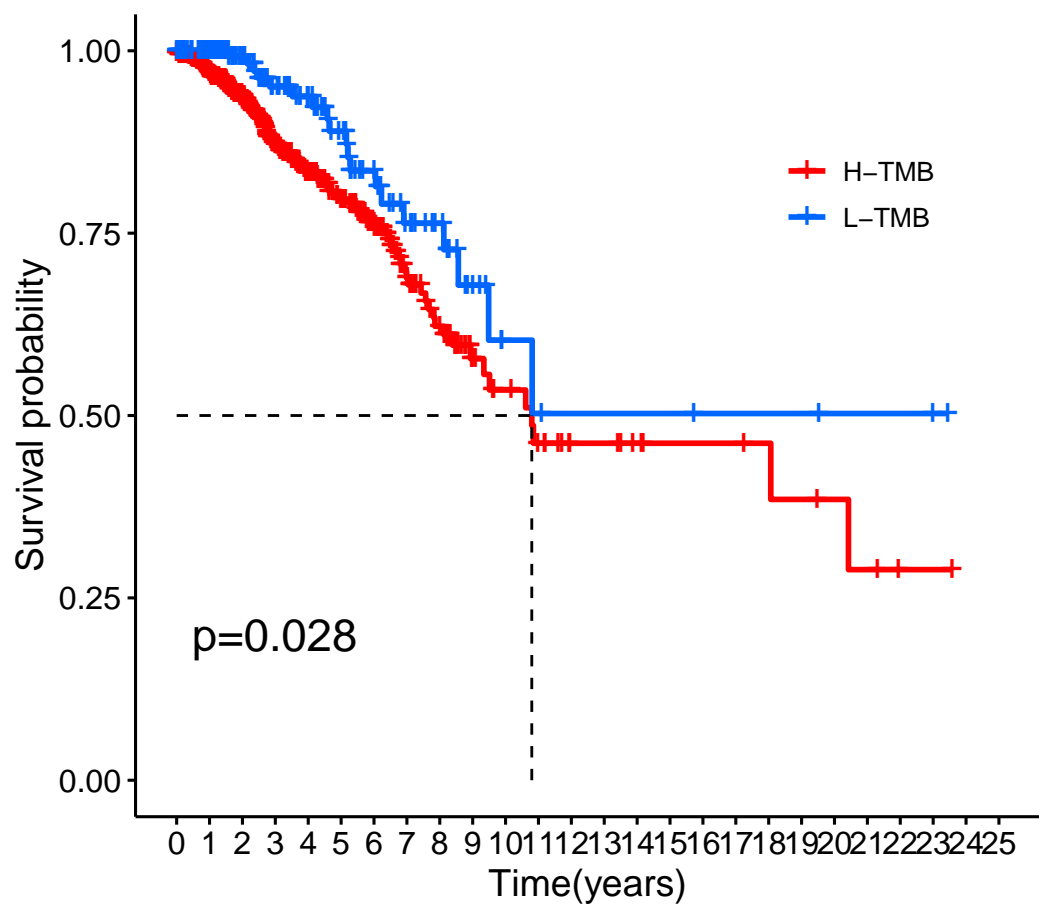

Supplement: Supplementary file 5 [file DataSheet_5.zip › 11.TUbian/TMB.survival.pdf]
