# Supplementary material for: Leveraging senescence-oxidative stress co-relation to predict prognosis and drug sensitivity in breast invasive carcinoma
Source: Front Endocrinol (Lausanne). 2023 Aug 4;14:1179050. doi: 10.3389/fendo.2023.1179050 (PMC10437062; doi:10.3389/fendo.2023.1179050)

***Supplementary Materials***

Primer pairs of the seven SOSCRGs:

ALOX15B:

Forward (5’-3’) CAGTGGAAGGCTTACAACCCA

Reverse (5’-3’) CAGTGCTCAAATGCGTGCT

ERRFI1:

Forward (5’-3’) CTGGAGCAGTCGCAGTGAG

Reverse (5’-3’) GCCATTCATCGGAGCAGATTTG

ETS1:

Forward (5’-3’) GATAGTTGTGATCGCCTCACC

Reverse (5’-3’) GTCCTCTGAGTCGAAGCTGTC

G6PD:

Forward (5’-3’) CGAGGCCGTCACCAAGAAC

Reverse (5’-3’) GTAGTGGTCGATGCGGTAGA

MAP2K6:

Forward (5’-3’) GAAGCATTTGAACAACCTCAGAC

Reverse (5’-3’) CCTGGCTATTTACTGTGGCTC

NDRG1:

Forward (5’-3’) CTCCTGCAAGAGTTTGATGTCC

Reverse (5’-3’) TCATGCCGATGTCATGGTAGG

ZMAT3:

Forward (5’-3’) AGAAGCCTTTTGGGCAGGAG

Reverse (5’-3’) TGCTGCATAGTAATTTCGGAGTT

**Supplementary Figure S1.** Protein-protein interaction (PPI) network of the SRGs and OSRGs. The blue bubbles represent senescence-related genes (SRGs) and the red bubbles represent oxidative stress-related genes (OSRGs).


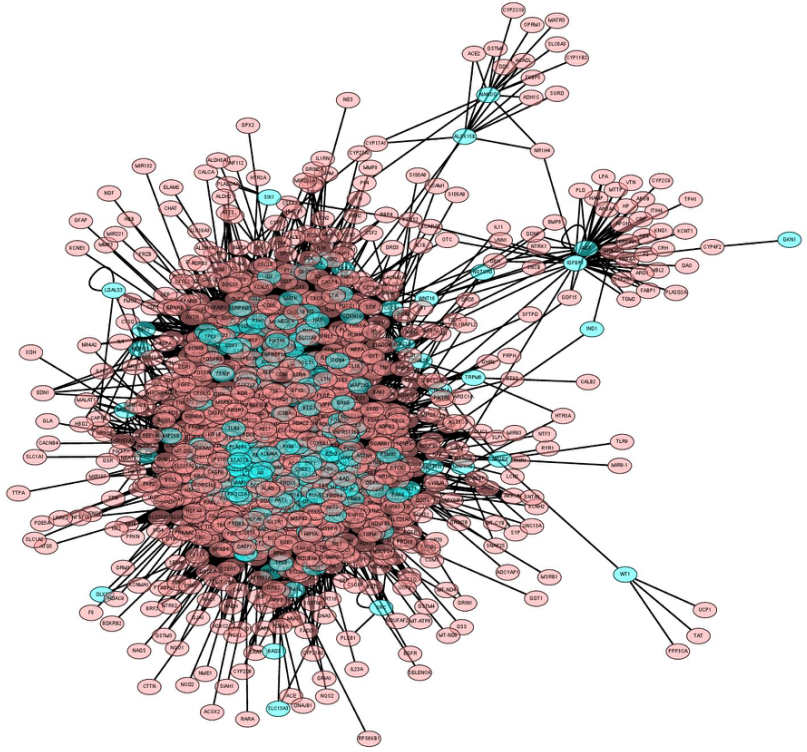


**Supplementary Figure S2.** ROC curves of the risk score and other clinicopathological features. **(A)** The training cohort. **(B)** The internal validation cohort. **(C)** The external validation cohort.


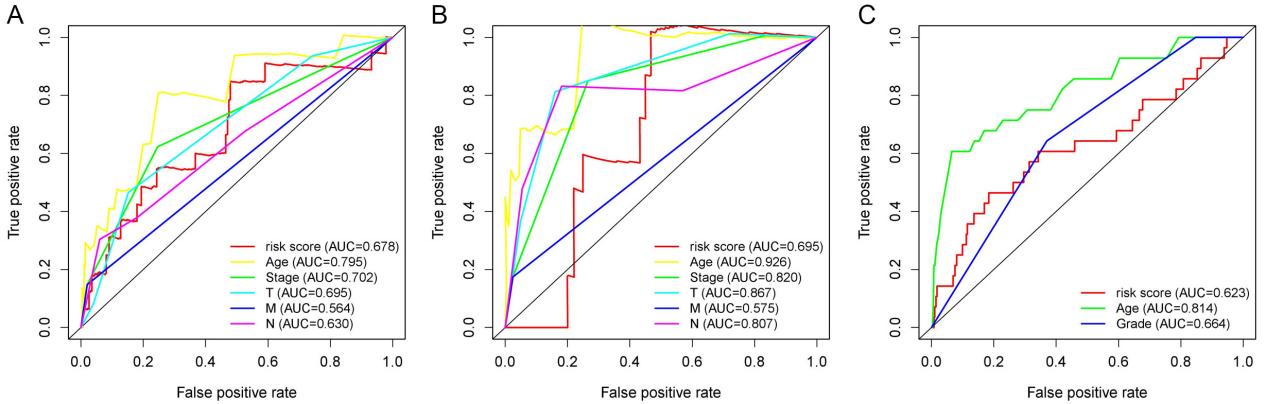


**Supplementary Figure S3.** Distribution of clinicopathological characteristics in the two risk groups in the training cohort.


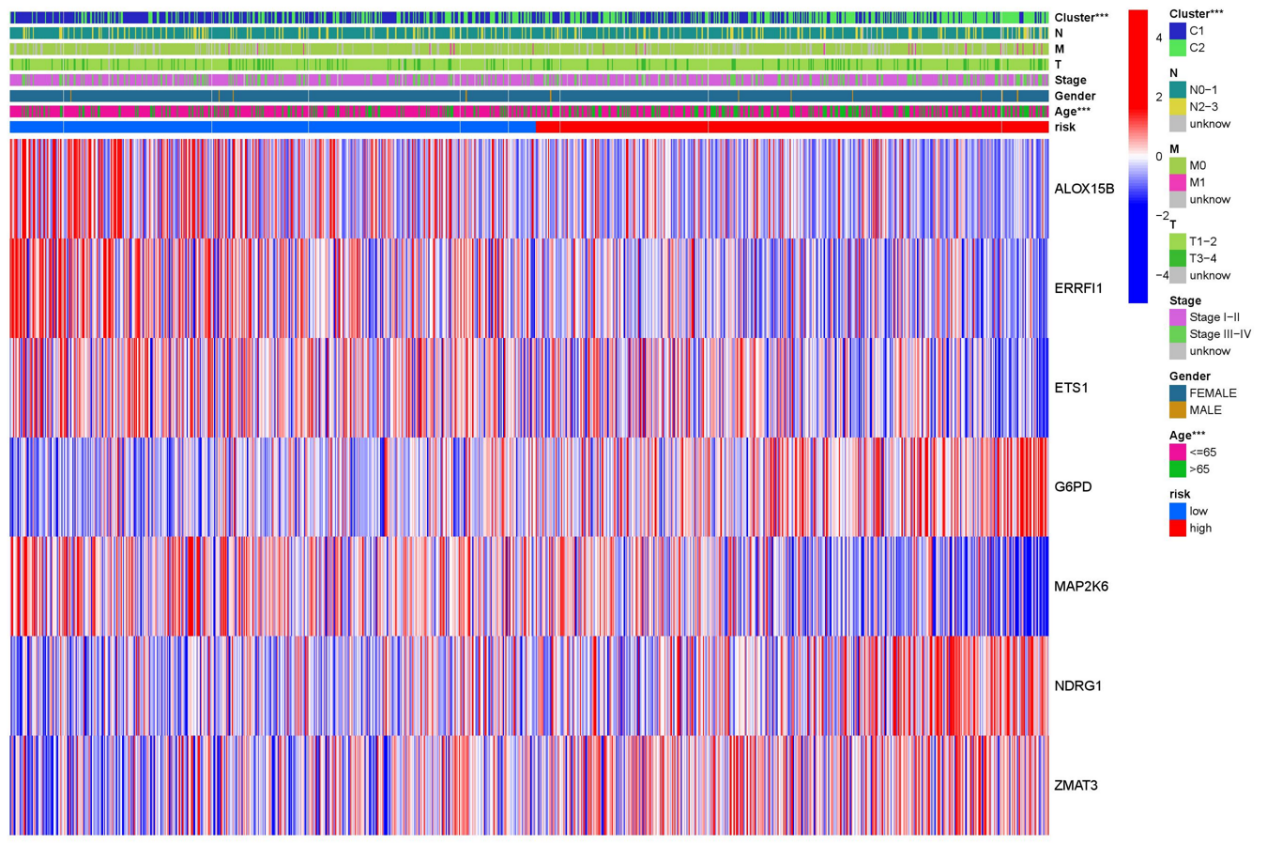


**Supplementary Figure S4.** Single-cell analysis.


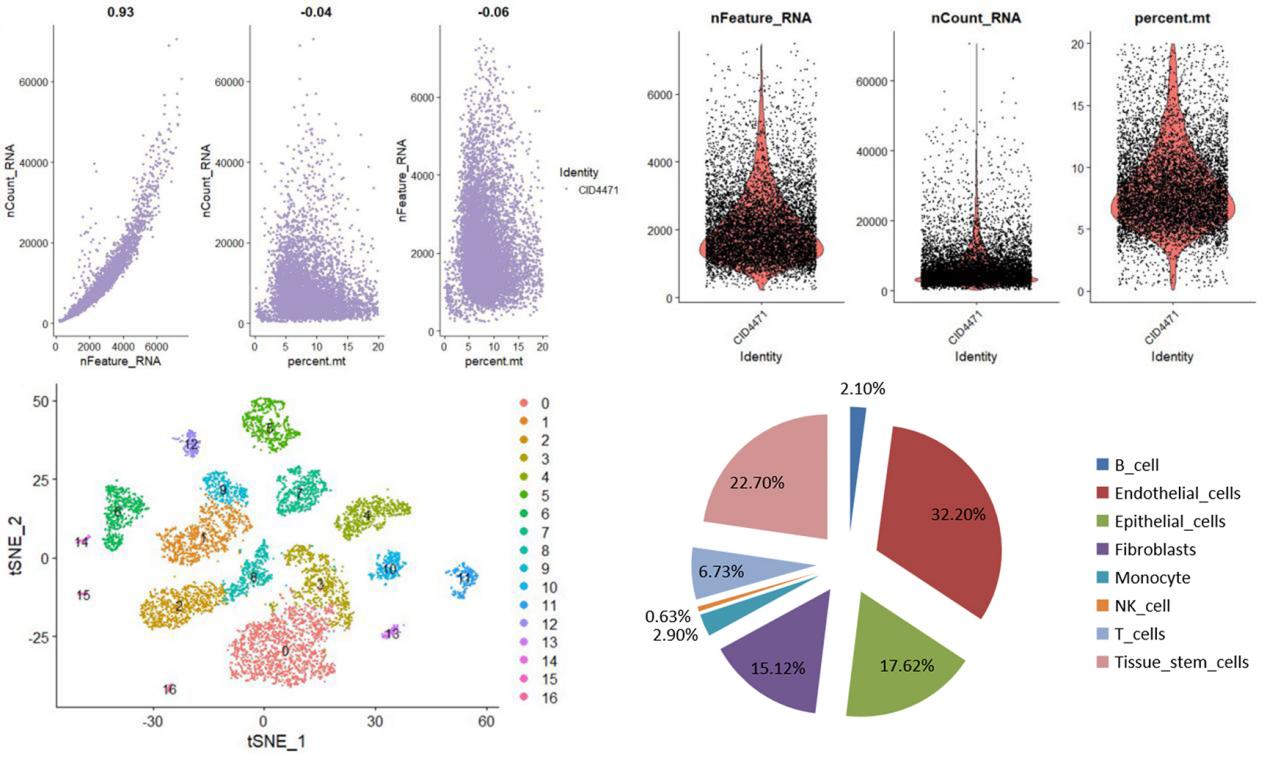

Supplement: Supplementary file 6 [file DataSheet_6.docx]
