# Supplementary material for: Leveraging senescence-oxidative stress co-relation to predict prognosis and drug sensitivity in breast invasive carcinoma
Source: Front Endocrinol (Lausanne). 2023 Aug 4;14:1179050. doi: 10.3389/fendo.2023.1179050 (PMC10437062; doi:10.3389/fendo.2023.1179050)
Supplement: Supplementary file 7 [file DataSheet_7.docx]

***Supplementary Materials***

1. **Primer design of RT-PCR：**

<https://www.ncbi.nlm.nih.gov/gene/>, obtaining Gene ID;

<https://pga.mgh.harvard.edu/primerbank/>, searching for Gene ID and acquiring primer sequences;

<https://www.ncbi.nlm.nih.gov/tools/primer-blast/index.cgi>, conducting Primer-BLAST of the acquired primer sequences, which were adopted if proper.

1. **Calculation formula:**

△CT=CT_target gene_-CT_CT average of corresponding internal reference gene_

△△CT=△CT (each group)-△CT (average of the control)

Fold gene expression=2^-△△CT^

1. **The usage of primers and qRT-PCR accorded with the ChamQ SYBR qPCR Master Mix (Low ROX Premixed) (vazyme) guideline. Reaction system and experimental setting were as follows:**

| Reaction system: | |
| --- | --- |
| 2 × ChamQ SYBR qPCR Master Mix (Low ROX Premixed) | 10.0 µl |
| Primer1 (10 µM) | 0.4 µl |
| Primer2 (10 µM) | 0.4 µl |
| Template DNA/cDNA | X µl |
| ddH2O | To20 µl |

| Experimental setting: | | | | |
| --- | --- | --- | --- | --- |
| Stage 1 | Denaturation | Rep: 1 | 95 ℃ | 30 sec |
| Stage 2 | Extension circulation | Reps: 40 | 95 ℃ | 10 sec |
|  |  |  | 60 ℃ | 30 sec |
| Stage 3 | Annealling | Reps: 1 | 95 ℃ | 15 sec |
|  |  |  | 60 ℃ | 60 sec |
|  |  |  | 95 ℃ | 15 sec |

Experimental optimization:

① The total amount of our RNA concentration reversal rate is 1 μg of RNA for reverse transcription, and it is diluted to 2-3 μl when the concentration of cDNA obtained after reversal is high.

② Our template is an undiluted cDNA stock solution, and the volume used is controlled as much as possible at 1/10 of the total volume of the qPCR reaction.

③ We adjusted the annealing temperature of stage 2 according to the primer TM value to achieve the best experimental results, and the primer TM value is as follows:

|  |  |  |  |  | TM |
| --- | --- | --- | --- | --- | --- |
| 247 | ALOX15B F | CAGTGGAAGGCTTACAACCCA | 21 |  | 60.2 |
|  | ALOX15B R | CAGTGCTCAAATGCGTGCT | 19 |  | 59.42 |
| 54206 | ERRFI1 F | CTGGAGCAGTCGCAGTGAG | 19 |  | 60.45 |
|  | ERRFI1 R | GCCATTCATCGGAGCAGATTTG | 22 |  | 60.29 |
| 2113 | ETS1 F | GATAGTTGTGATCGCCTCACC | 21 |  | 58.53 |
|  | ETS1 R | GTCCTCTGAGTCGAAGCTGTC | 22 |  | 60.14 |
| 2539 | G6PD F | CGAGGCCGTCACCAAGAAC | 19 |  | 61.03 |
|  | G6PD R | GTAGTGGTCGATGCGGTAGA | 20 |  | 58.98 |
| 5608 | MAP2K6 F | GAAGCATTTGAACAACCTCAGAC | 23 |  | 58.46 |
|  | MAP2K6 R | CCTGGCTATTTACTGTGGCTC | 21 |  | 58.43 |
| 10397 | NDRG1 F | CTCCTGCAAGAGTTTGATGTCC | 22 |  | 59.25 |
|  | NDRG1 R | TCATGCCGATGTCATGGTAGG | 21 |  | 59.93 |
| 64393 | ZMAT3 F | AGAAGCCTTTTGGGCAGGAG | 20 |  | 60.25 |
|  | ZMAT3 R | TGCTGCATAGTAATTTCGGAGTT | 23 |  | 58.49 |
|  |  |  |  |  | TM |
| 247 | ALOX15B F | CAGTGGAAGGCTTACAACCCA | 21 |  | 60.2 |
|  | ALOX15B R | CAGTGCTCAAATGCGTGCT | 19 |  | 59.42 |
| 54206 | ERRFI1 F | CTGGAGCAGTCGCAGTGAG | 19 |  | 60.45 |
|  | ERRFI1 R | GCCATTCATCGGAGCAGATTTG | 22 |  | 60.29 |
| 2113 | ETS1 F | GATAGTTGTGATCGCCTCACC | 21 |  | 58.53 |
|  | ETS1 R | GTCCTCTGAGTCGAAGCTGTC | 22 |  | 60.14 |
| 2539 | G6PD F | CGAGGCCGTCACCAAGAAC | 19 |  | 61.03 |
|  | G6PD R | GTAGTGGTCGATGCGGTAGA | 20 |  | 58.98 |
| 5608 | MAP2K6 F | GAAGCATTTGAACAACCTCAGAC | 23 |  | 58.46 |
|  | MAP2K6 R | CCTGGCTATTTACTGTGGCTC | 21 |  | 58.43 |
| 10397 | NDRG1 F | CTCCTGCAAGAGTTTGATGTCC | 22 |  | 59.25 |
|  | NDRG1 R | TCATGCCGATGTCATGGTAGG | 21 |  | 59.93 |
| 64393 | ZMAT3 F | AGAAGCCTTTTGGGCAGGAG | 20 |  | 60.25 |
|  | ZMAT3 R | TGCTGCATAGTAATTTCGGAGTT | 23 |  | 58.49 |
